# Supplementary material for: Single-cell transcriptome analysis of Physcomitrella leaf cells during reprogramming using microcapillary manipulation
Source: Nucleic Acids Res. 2019 Mar 15;47(9):4539–53. doi: 10.1093/nar/gkz181 (PMC6511839; doi:10.1093/nar/gkz181)
Supplement: Supplementary Data [file gkz181_supplemental_files.zip › Supplementary data_Kubo _revsion.pdf]

Supplementary Data

Supplementary Tables S1 to S14.

Supplementary Table S1. Oligo DNAs used in this work.

Supplementary Table S2. Statistics of pilot sequencing for 1cell-DGE.

Supplementary Table S3. NGS statistics of 1cell-DGE data derived from individual leaf cells at 0 h and 24 h after leaf excision.

Supplementary Table S4. DEGs in 24 h-high identified using 1cell-DGE categorized by the GO term "cell growth".

Supplementary Table S5. DEGs in 24 h-high identified using 1cell-DGE categorized by the GO term "cell cycle".

Supplementary Table S6. DEGs in 24 h-high identified using 1cell-DGE categorized by the GO term "cell differentiation".

Supplementary Table S7. DEGs in 24 h-high identified using 1cell-DGE categorized by the GO term "embryonic development".

Supplementary Table S8. DEGs in 24 h-high identified using 1cell-DGE categorized by the GO term "DNA metabolic process".

Supplementary Table S9. DEGs in 24 h-high identified using 1cell-DGE categorized by the GO term "protein metabolic process".

Supplementary Table S10. DEGs in 24 h-high identified using 1cell-DGE categorized by the GO term "biosynthetic process".

Supplementary Table S11. DEGs in 24 h-high identified using 1cell-DGE categorized by the GO term "translation".

Supplementary Table S12. DEGs in 24 h-high identified using 1cell-DGE categorized by the GO term "carbohydrate metabolic process".

Supplementary Table S13. DEGs in 24 h-high identified using 1cell-DGE categorized by the GO term "anatomical structure morphogenesis".

Supplementary Table S14. DEGs in 24 h-high identified using 1cell-DGE categorized by the GO term "cellular component organization".

Supplementary Figures S1 to S9.

Supplementary Figure S1. Extraction of the contents of an individual cell using microcapillary manipulation.

Supplementary Figure S2. Transcript levels of *NGG, PpEF1α, PpCYCD;1,* and *PpTUA1* in the contents of single cells.
Supplementary Figure S3. Abundance of *PpTUA1* cDNA in the samples reverse-transcribed from different concentrations of total RNA.
Supplementary Figure S4. Validation of 1cell-DGE by pilot sequencing.
Supplementary Figure S5. Statistics of 1cell-DGE performed on single leaf cells from Physcomitrella.
Supplementary Figure S6. Quality check of 1cell-DGE data.
Supplementary Figure S7. Estimation of sequencing coverage using 1cell-DGE.
Supplementary Figure S8. Independent component analyses of 1cell-DGE data.
Supplementary Figure S9. Correlation between pseudotime and time for sampling at 0 h.

Supplementary Movie

Supplementary Movie S1. Extraction of the contents of a single cell in an excised leaf of Physcomitrella using a microcapillary. GFP fluorescence was observed using the fluorescence filter unit (see Methods) under a mercury arc lamp and with dim bright-field illumination.

Supplementary Figure Legends

Supplementary Figure S1. Extraction of the contents of an individual cell using microcapillary manipulation. An arrow indicates the tip of a microcapillary extracting the contents of a cell facing the cut edge of an excised Physcomitrella leaf expressing GFP in its nuclei. Imaged using GFP fluorescence under dim bright-field illumination. Scale bar: 50 μm.

Supplementary Figure S2. Transcript levels of *NGG*, *PpEF1α*, *PpCYCD;1*, and *PpTUA1* in the contents of single cells. (a) Dot plots show the mean number of transcripts converted to cDNA using reverse transcription, derived from technical triplicates measured using qPCR. The numbers along the x axis represent 10 independent samples derived from the contents of single cells. Error bars indicate the standard deviation derived from technical triplicates. (b) Jitter plots show the mean Cp values of the transcripts. Each dot represents an independent single cell sample.

Supplementary Figure S3. Abundance of *PpTUA1* cDNA in the samples reverse-transcribed from different concentrations of total RNA. The jitter plot shows the Cp values as the abundance of *PpTUA1* cDNA derived from different concentrations of total RNA. The cDNA was generated with either poly(dA) tailing or template switching.

Supplementary Figure S4. Validation of 1cell-DGE by pilot sequencing. Scatter plots of the expression profiles of Physcomitrella genes (a, b) or the ERCC RNA spike-in mix (c–h) derived from 5 μg and 20 pg of total RNA. The 20 pg total RNA was diluted from 5 μg of total RNA including a 0.1-fold ERCC RNA spike-in mix with nuclease-free water. Illumina sequencing libraries of replicate 1 (Rep1) and replicate 2 (Rep2) were prepared from the same RNA stock solution. For the 5 μg of samples, the cDNA amplification step was skipped when generating the 1-cell cDNA libraries (see Supplementary Figure S4, Methods) was skipped. (a, b, c, d) Rep1 and Rep2 of total read (grey) and UMI counts (black) in which the unified duplicated reads with the same UMI mapped on the same gene locus were plotted. (e, f, g, h) UMI counts of the ERCC RNA spike-in mix in each replicate were plotted against the concentration of the ERCC RNA spike-in mix. R^2 indicates the determination coefficients.

Supplementary Figure S5. Statistics of 1cell-DGE performed on single leaf cells from Physcomitrella. Box and jitter plots indicate the statistics of the 1cell-DGE data generated for 32 and 34 individual cells sampled at 0 h and 24 h after leaf excision. These read counts include the reads mapped onto the Physcomitrella v.3.3 gene models [39] and the ERCC RNA spike-in mix before their unification using the UMIs. (a) Total read counts. (b) Mapped read counts. (c) Mapping rate (Supplementary Table S2).

Supplementary Figure S6. Quality check of 1cell-DGE data. Box and jitter plots show (a) the total number of UMI counts, (b) the UMI-unified rate, (c) the number of detected genes, and (d) the read complexity of the 32 and 34 individual cells sampled at 0 h and 24 h after leaf excision, respectively (Supplementary Table S2). These statistics were calculated using the SinQC package [41] and the following settings: Max

FPR: 0.05, TPM Cutoff: 1, Spearman’s test p-value: <0.001, and Pearson’s test p-value: <0.001.

Supplementary Figure S7. Estimation of sequencing coverage using 1cell-DGE. The relationships between the sequencing depth and (a, b) the number of detected genes and (c, d) the UMI-unified rate are presented. 0.1, 0.2, 0.5, 1.0, 2.0, and 5.0 million reads were randomly sampled from 1cell-DGE data derived from the individual leaf cells Pp0h07, Pp0h08, Pp0h42 at 0 h after excision (a, c) and Pp24h07, Pp24h08, Pp24h42 at 24 h after excision (b, d). These sample data were processed using the UMI_SC package (Nishiyama, 2016: https://github.com/tomoakin/UMI_SC) and were counted using the SinQC program [41]. Triangles represent Pp0h07 and Pp24h07 values with the index sequence ATACGTGC. Squares represent Pp0h08 and Pp24h08 values with the index sequence CGTGCATA. Circles represent Pp0h42 and Pp24h42 values with the index sequence ACTTCGGT.

Supplementary Figure S8. Independent component analyses of 1cell-DGE data. 1cell-DGE data were calculated to reduce the dimensions of the expression profiles using an independent component analysis and plotted using the monocle v1.4 package [43]. Each dot indicates independent cell samples which were colored by (a) their extracted nuclear condition, (b) their leaf excision date, (c) the byproduct contamination, and (d) the cDNA amounts. Circles represent cells sampled 0 h after leaf excision, triangles represent cells sampled 24 h after leaf excision.

Supplementary Figure S9. Correlation between pseudotime and time of sampling at 0 h. The pseudotime of the 1cell-DGE data were plotted against the times of sampling, which were the time intervals between the leaf cut to the extraction of the cell contents. Each dot indicates an independent cell sampled 0 h after leaf excision. ρ indicates the Spearman correlation coefficient.

Supplementary Table S1. Oligo DNAs used in this work.

Experiments	Oligo name	Sequence (5' ->3') *1	Modification	Purification	Purpose	Index sequence*2
qPCR	GUS-F1285	GCGGAAGCAACGCGTAAA			RT-qPCR	
	GUS-R1353	AGCGTCGCAGAACATTACATTG			RT-qPCR	
	CYCD1-F1387	AGAGCGCCAGCCTATAAAGTG			RT-qPCR	
	CYCD1-R1446	CAACGGAAACTGCCAGTGAA			RT-qPCR	
	EF1a-F1406	GGGTGTTATCAAGGCGGTAGAG			RT-qPCR	
	EF1a-R1475	CTTCTTGGCAGCGGCCTTAGT			RT-qPCR	
	TUA1-F1424	CGTAGGAGGGACCAGTTTGG			RT-qPCR	
	TUA1-R1485	TGCATTCATCCCCGAGTCA			RT-qPCR	
pilot sequencing	BPUIRO-01	ACGCGCAAGCAGAAGACGGCATACGAGATNNNUNNNNUNNNCGTGATGTGACTGGAGTTCAGACGTGTGCTCTTCCGATCTTTTTTTTTTTTTTTTTTTT	dU-inserted, Biotin-TEG-link at 5' end	HPLC	RT oligos	
	BPUIRO-02	ACGCGCAAGCAGAAGACGGCATACGAGATNNNUNNNNUNNNACATCGGTGACTGGAGTTCAGACGTGTGCTCTTCCGATCTTTTTTTTTTTTTTTTTTTT	dU-inserted, Biotin-TEG-link at 5' end	HPLC	RT oligos	
	PUIRO-03	ACGCGCAAGCAGAAGACGGCATACGAGATNNNUNNNNUNNNGCCTAAGTGACTGGAGTTCAGACGTGTGCTCTTCCGATCTTTTTTTTTTTTTTTTTTTT	dU-inserted	HPLC	RT oligos	
	PUIRO-04	ACGCGCAAGCAGAAGACGGCATACGAGATNNNUNNNNUNNNTGGTCAGTGACTGGAGTTCAGACGTGTGCTCTTCCGATCTTTTTTTTTTTTTTTTTTTT	dU-inserted	HPLC	RT oligos	
	SMART IV oligo	AAGCAGTGGTATCAACGCAGAGTGGCCATTACGGCCrGrGrG	rG:ribonucleotides, Amino-link at 5' end	HPLC	Template switching	
1cell-DGE	NUP3	AAGCAGTGGTATCAACGCAGAGTGGCTTTTTTTTTTTTTTTTTTTTTTTT	Amino-link at 5' end	HPLC	2nd strand synthesis for poly(dA) tailing	
	BTEP7v2	ACGCGCAAGCAGAAGACGGCATACGAGAT	Biotin-link at 5' end	HPLC	Amplification	
	EP7v2	ACGCGCAAGCAGAAGACGGCATACGAGAT		HPLC	NGS library enrichment	
	RP1 adaptor v2	GATCGGAAGAGCGTCGUACACTCTTTCCCTACACGACGCTCTTCCGATCT	dU-inserted	HPLC	Adaptor ligation	
	P5RP1	AATGATACGGCGACCACCGAGATCTACACTCTTTCCCTACACGACGCTCTTCCGATCT		HPLC	NGS library enrichment	
	NPUIRO-07	ACGCGCAAGCAGAAGACGGCATACGAGATNNNNNNNNNNGCACGTATGTGACTGGAGTTCAGACGTGTGCTCTTCCGATCTTTTTTTTTTTTTTTTTTTT		PAGE	RT oligos	ATACGTGC
	NPUIRO-08	ACGCGCAAGCAGAAGACGGCATACGAGATNNNNNNNNNNTATGCACGGTGACTGGAGTTCAGACGTGTGCTCTTCCGATCTTTTTTTTTTTTTTTTTTTT		PAGE	RT oligos	CGTGCATA
	NPUIRO-09	ACGCGCAAGCAGAAGACGGCATACGAGATNNNNNNNNNNAGCAGTTCGTGACTGGAGTTCAGACGTGTGCTCTTCCGATCTTTTTTTTTTTTTTTTTTTT		PAGE	RT oligos	GAACTGCT
	NPUIRO-10	ACGCGCAAGCAGAAGACGGCATACGAGATNNNNNNNNNNACGTTCCTGTGACTGGAGTTCAGACGTGTGCTCTTCCGATCTTTTTTTTTTTTTTTTTTTT		PAGE	RT oligos	AGGAACGT
	NPUIRO-11	ACGCGCAAGCAGAAGACGGCATACGAGATNNNNNNNNNNACTACGGAGTGACTGGAGTTCAGACGTGTGCTCTTCCGATCTTTTTTTTTTTTTTTTTTTT		PAGE	RT oligos	TCCGTAGT
	NPUIRO-12	ACGCGCAAGCAGAAGACGGCATACGAGATNNNNNNNNNNTTCGACTGGTGACTGGAGTTCAGACGTGTGCTCTTCCGATCTTTTTTTTTTTTTTTTTTTT		PAGE	RT oligos	CAGTCGAA
	NPUIRO-13	ACGCGCAAGCAGAAGACGGCATACGAGATNNNNNNNNNNTCCATGCTGTGACTGGAGTTCAGACGTGTGCTCTTCCGATCTTTTTTTTTTTTTTTTTTTT		PAGE	RT oligos	AGCATGGA
	NPUIRO-14	ACGCGCAAGCAGAAGACGGCATACGAGATNNNNNNNNNNCAATTCCGGTGACTGGAGTTCAGACGTGTGCTCTTCCGATCTTTTTTTTTTTTTTTTTTTT		PAGE	RT oligos	CGGAATTG
	NPUIRO-15	ACGCGCAAGCAGAAGACGGCATACGAGATNNNNNNNNNNGTGAAGGAGTGACTGGAGTTCAGACGTGTGCTCTTCCGATCTTTTTTTTTTTTTTTTTTTT		PAGE	RT oligos	TCCTTCAC
	NPUIRO-16	ACGCGCAAGCAGAAGACGGCATACGAGATNNNNNNNNNNGGTTACACGTGACTGGAGTTCAGACGTGTGCTCTTCCGATCTTTTTTTTTTTTTTTTTTTT		PAGE	RT oligos	GTGTAACC
	NPUIRO-17	ACGCGCAAGCAGAAGACGGCATACGAGATNNNNNNNNNNCCAGTCTTGTGACTGGAGTTCAGACGTGTGCTCTTCCGATCTTTTTTTTTTTTTTTTTTTT		PAGE	RT oligos	AAGACTGG
	NPUIRO-18	ACGCGCAAGCAGAAGACGGCATACGAGATNNNNNNNNNNCTTCCGTTGTGACTGGAGTTCAGACGTGTGCTCTTCCGATCTTTTTTTTTTTTTTTTTTTT		PAGE	RT oligos	AACGGAAG
	NPUIRO-19	ACGCGCAAGCAGAAGACGGCATACGAGATNNNNNNNNNNTCCTTCACGTGACTGGAGTTCAGACGTGTGCTCTTCCGATCTTTTTTTTTTTTTTTTTTTT		PAGE	RT oligos	GTGAAGGA
	NPUIRO-20	ACGCGCAAGCAGAAGACGGCATACGAGATNNNNNNNNNNTGTAGACCGTGACTGGAGTTCAGACGTGTGCTCTTCCGATCTTTTTTTTTTTTTTTTTTTT		PAGE	RT oligos	GGTCTACA
	NPUIRO-21	ACGCGCAAGCAGAAGACGGCATACGAGATNNNNNNNNNNCCATTGGAGTGACTGGAGTTCAGACGTGTGCTCTTCCGATCTTTTTTTTTTTTTTTTTTTT		PAGE	RT oligos	TCCAATGG
	NPUIRO-22	ACGCGCAAGCAGAAGACGGCATACGAGATNNNNNNNNNNACTCTCTCGTGACTGGAGTTCAGACGTGTGCTCTTCCGATCTTTTTTTTTTTTTTTTTTTT		PAGE	RT oligos	GAGAGAGT
	NPUIRO-23	ACGCGCAAGCAGAAGACGGCATACGAGATNNNNNNNNNNGGATATCGGTGACTGGAGTTCAGACGTGTGCTCTTCCGATCTTTTTTTTTTTTTTTTTTTT		PAGE	RT oligos	CGATATCC
	NPUIRO-24	ACGCGCAAGCAGAAGACGGCATACGAGATNNNNNNNNNNTAGGACCTGTGACTGGAGTTCAGACGTGTGCTCTTCCGATCTTTTTTTTTTTTTTTTTTTT		PAGE	RT oligos	AGGTCCTA
	NPUIRO-25	ACGCGCAAGCAGAAGACGGCATACGAGATNNNNNNNNNNTACATCGGGTGACTGGAGTTCAGACGTGTGCTCTTCCGATCTTTTTTTTTTTTTTTTTTTT		PAGE	RT oligos	CCGATGTA
	NPUIRO-26	ACGCGCAAGCAGAAGACGGCATACGAGATNNNNNNNNNNCGCGTTAAGTGACTGGAGTTCAGACGTGTGCTCTTCCGATCTTTTTTTTTTTTTTTTTTTT		PAGE	RT oligos	TTAACGCG
	NPUIRO-27	ACGCGCAAGCAGAAGACGGCATACGAGATNNNNNNNNNNATTCCAGGGTGACTGGAGTTCAGACGTGTGCTCTTCCGATCTTTTTTTTTTTTTTTTTTTT		PAGE	RT oligos	CCTGGAAT
	NPUIRO-28	ACGCGCAAGCAGAAGACGGCATACGAGATNNNNNNNNNNGAAGGAAGGTGACTGGAGTTCAGACGTGTGCTCTTCCGATCTTTTTTTTTTTTTTTTTTTT		PAGE	RT oligos	CTTCCTTC
	NPUIRO-29	ACGCGCAAGCAGAAGACGGCATACGAGATNNNNNNNNNNAGATTGCCGTGACTGGAGTTCAGACGTGTGCTCTTCCGATCTTTTTTTTTTTTTTTTTTTT		PAGE	RT oligos	GGCAATCT
	NPUIRO-30	ACGCGCAAGCAGAAGACGGCATACGAGATNNNNNNNNNNACGCATGAGTGACTGGAGTTCAGACGTGTGCTCTTCCGATCTTTTTTTTTTTTTTTTTTTT		PAGE	RT oligos	TCATGCGT
	NPUIRO-31	ACGCGCAAGCAGAAGACGGCATACGAGATNNNNNNNNNNGGATCCTTGTGACTGGAGTTCAGACGTGTGCTCTTCCGATCTTTTTTTTTTTTTTTTTTTT		PAGE	RT oligos	AAGGATCC
	NPUIRO-32	ACGCGCAAGCAGAAGACGGCATACGAGATNNNNNNNNNNAAGAGTCGGTGACTGGAGTTCAGACGTGTGCTCTTCCGATCTTTTTTTTTTTTTTTTTTTT		PAGE	RT oligos	CGACTCTT
	NPUIRO-33	ACGCGCAAGCAGAAGACGGCATACGAGATNNNNNNNNNNGAGTCATCGTGACTGGAGTTCAGACGTGTGCTCTTCCGATCTTTTTTTTTTTTTTTTTTTT		PAGE	RT oligos	GATGACTC
	NPUIRO-34	ACGCGCAAGCAGAAGACGGCATACGAGATNNNNNNNNNNATACGTCCGTGACTGGAGTTCAGACGTGTGCTCTTCCGATCTTTTTTTTTTTTTTTTTTTT		PAGE	RT oligos	GGACGTAT
	NPUIRO-35	ACGCGCAAGCAGAAGACGGCATACGAGATNNNNNNNNNNTCGTCAGTGTGACTGGAGTTCAGACGTGTGCTCTTCCGATCTTTTTTTTTTTTTTTTTTTT		PAGE	RT oligos	ACTGACGA
	NPUIRO-36	ACGCGCAAGCAGAAGACGGCATACGAGATNNNNNNNNNNATTGAGCCGTGACTGGAGTTCAGACGTGTGCTCTTCCGATCTTTTTTTTTTTTTTTTTTTT		PAGE	RT oligos	GGCTCAAT
	NPUIRO-37	ACGCGCAAGCAGAAGACGGCATACGAGATNNNNNNNNNNTTCCGCAAGTGACTGGAGTTCAGACGTGTGCTCTTCCGATCTTTTTTTTTTTTTTTTTTTT		PAGE	RT oligos	TTGCGGAA
	NPUIRO-38	ACGCGCAAGCAGAAGACGGCATACGAGATNNNNNNNNNNCAGTATGGGTGACTGGAGTTCAGACGTGTGCTCTTCCGATCTTTTTTTTTTTTTTTTTTTT		PAGE	RT oligos	CCATACTG
	NPUIRO-39	ACGCGCAAGCAGAAGACGGCATACGAGATNNNNNNNNNNCTTGGAACGTGACTGGAGTTCAGACGTGTGCTCTTCCGATCTTTTTTTTTTTTTTTTTTTT		PAGE	RT oligos	GTTCCAAG
	NPUIRO-40	ACGCGCAAGCAGAAGACGGCATACGAGATNNNNNNNNNNTCACCTTGGTGACTGGAGTTCAGACGTGTGCTCTTCCGATCTTTTTTTTTTTTTTTTTTTT		PAGE	RT oligos	CAAGGTGA
	NPUIRO-41	ACGCGCAAGCAGAAGACGGCATACGAGATNNNNNNNNNNGTTCTGACGTGACTGGAGTTCAGACGTGTGCTCTTCCGATCTTTTTTTTTTTTTTTTTTTT		PAGE	RT oligos	GTCAGAAC
	NPUIRO-42	ACGCGCAAGCAGAAGACGGCATACGAGATNNNNNNNNNNACCGAAGTGTGACTGGAGTTCAGACGTGTGCTCTTCCGATCTTTTTTTTTTTTTTTTTTTT		PAGE	RT oligos	ACTTCGGT
	NPUIRO-43	ACGCGCAAGCAGAAGACGGCATACGAGATNNNNNNNNNNCGTGATTCGTGACTGGAGTTCAGACGTGTGCTCTTCCGATCTTTTTTTTTTTTTTTTTTTT		PAGE	RT oligos	GAATCACG
	NPUIRO-44	ACGCGCAAGCAGAAGACGGCATACGAGATNNNNNNNNNNTCGAGAAGGTGACTGGAGTTCAGACGTGTGCTCTTCCGATCTTTTTTTTTTTTTTTTTTTT		PAGE	RT oligos	CTTCTCGA
	NPUIRO-45	ACGCGCAAGCAGAAGACGGCATACGAGATNNNNNNNNNNCTCAAGTCGTGACTGGAGTTCAGACGTGTGCTCTTCCGATCTTTTTTTTTTTTTTTTTTTT		PAGE	RT oligos	GACTTGAG
	NPUIRO-46	ACGCGCAAGCAGAAGACGGCATACGAGATNNNNNNNNNNACTGTGAGGTGACTGGAGTTCAGACGTGTGCTCTTCCGATCTTTTTTTTTTTTTTTTTTTT		PAGE	RT oligos	CTCACAGT
	NPUIRO-47	ACGCGCAAGCAGAAGACGGCATACGAGATNNNNNNNNNNGTACCACTGTGACTGGAGTTCAGACGTGTGCTCTTCCGATCTTTTTTTTTTTTTTTTTTTT		PAGE	RT oligos	AGTGGTAC
	NPUIRO-48	ACGCGCAAGCAGAAGACGGCATACGAGATNNNNNNNNNNCCTTACTGGTGACTGGAGTTCAGACGTGTGCTCTTCCGATCTTTTTTTTTTTTTTTTTTTT		PAGE	RT oligos	CAGTAAGG
	NPUIRO-49	ACGCGCAAGCAGAAGACGGCATACGAGATNNNNNNNNNNAAGCGGAAGTGACTGGAGTTCAGACGTGTGCTCTTCCGATCTTTTTTTTTTTTTTTTTTTT		PAGE	RT oligos	TTCCGCTT
	NPUIRO-50	ACGCGCAAGCAGAAGACGGCATACGAGATNNNNNNNNNNGTAACTGGGTGACTGGAGTTCAGACGTGTGCTCTTCCGATCTTTTTTTTTTTTTTTTTTTT		PAGE	RT oligos	CCAGTTAC
	NPUIRO-51	ACGCGCAAGCAGAAGACGGCATACGAGATNNNNNNNNNNCGGAACAAGTGACTGGAGTTCAGACGTGTGCTCTTCCGATCTTTTTTTTTTTTTTTTTTTT		PAGE	RT oligos	TTGTTCCG
	NPUIRO-52	ACGCGCAAGCAGAAGACGGCATACGAGATNNNNNNNNNNGCTATAGGGTGACTGGAGTTCAGACGTGTGCTCTTCCGATCTTTTTTTTTTTTTTTTTTTT		PAGE	RT oligos	CCTATAGC
	NPUIRO-53	ACGCGCAAGCAGAAGACGGCATACGAGATNNNNNNNNNNCGGTTGTTGTGACTGGAGTTCAGACGTGTGCTCTTCCGATCTTTTTTTTTTTTTTTTTTTT		PAGE	RT oligos	AACAACCG
	NPUIRO-54	ACGCGCAAGCAGAAGACGGCATACGAGATNNNNNNNNNNAGCACACAGTGACTGGAGTTCAGACGTGTGCTCTTCCGATCTTTTTTTTTTTTTTTTTTTT		PAGE	RT oligos	TGTGTGCT
	NPUIRO-55	ACGCGCAAGCAGAAGACGGCATACGAGATNNNNNNNNNNGTCGTGTTGTGACTGGAGTTCAGACGTGTGCTCTTCCGATCTTTTTTTTTTTTTTTTTTTT		PAGE	RT oligos	AACACGAC
	NPUIRO-56	ACGCGCAAGCAGAAGACGGCATACGAGATNNNNNNNNNNAAGACCACGTGACTGGAGTTCAGACGTGTGCTCTTCCGATCTTTTTTTTTTTTTTTTTTTT		PAGE	RT oligos	GTGGTCTT
	NPUIRO-57	ACGCGCAAGCAGAAGACGGCATACGAGATNNNNNNNNNNACCTGGTTGTGACTGGAGTTCAGACGTGTGCTCTTCCGATCTTTTTTTTTTTTTTTTTTTT		PAGE	RT oligos	AACCAGGT
	NPUIRO-58	ACGCGCAAGCAGAAGACGGCATACGAGATNNNNNNNNNNCACTGACAGTGACTGGAGTTCAGACGTGTGCTCTTCCGATCTTTTTTTTTTTTTTTTTTTT		PAGE	RT oligos	TGTCAGTG
	NPUIRO-59	ACGCGCAAGCAGAAGACGGCATACGAGATNNNNNNNNNNGAGAGGTTGTGACTGGAGTTCAGACGTGTGCTCTTCCGATCTTTTTTTTTTTTTTTTTTTT		PAGE	RT oligos	AACCTCTC
	NPUIRO-60	ACGCGCAAGCAGAAGACGGCATACGAGATNNNNNNNNNNGTGTTCTGGTGACTGGAGTTCAGACGTGTGCTCTTCCGATCTTTTTTTTTTTTTTTTTTTT		PAGE	RT oligos	CAGAACAC
	NPUIRO-61	ACGCGCAAGCAGAAGACGGCATACGAGATNNNNNNNNNNGTATGGTCGTGACTGGAGTTCAGACGTGTGCTCTTCCGATCTTTTTTTTTTTTTTTTTTTT		PAGE	RT oligos	GACCATAC
	NPUIRO-62	ACGCGCAAGCAGAAGACGGCATACGAGATNNNNNNNNNNTGAACCAGGTGACTGGAGTTCAGACGTGTGCTCTTCCGATCTTTTTTTTTTTTTTTTTTTT		PAGE	RT oligos	CTGGTTCA
	NPUIRO-63	ACGCGCAAGCAGAAGACGGCATACGAGATNNNNNNNNNNAACGCCTTGTGACTGGAGTTCAGACGTGTGCTCTTCCGATCTTTTTTTTTTTTTTTTTTTT		PAGE	RT oligos	AAGGCGTT
	NPUIRO-64	ACGCGCAAGCAGAAGACGGCATACGAGATNNNNNNNNNNAACCTACGGTGACTGGAGTTCAGACGTGTGCTCTTCCGATCTTTTTTTTTTTTTTTTTTTT		PAGE	RT oligos	CGTAGGTT
	NPUIRO-65	ACGCGCAAGCAGAAGACGGCATACGAGATNNNNNNNNNNGCTGCATTGTGACTGGAGTTCAGACGTGTGCTCTTCCGATCTTTTTTTTTTTTTTTTTTTT		PAGE	RT oligos	AATGCAGC
	NPUIRO-66	ACGCGCAAGCAGAAGACGGCATACGAGATNNNNNNNNNNCATAACGCGTGACTGGAGTTCAGACGTGTGCTCTTCCGATCTTTTTTTTTTTTTTTTTTTT		PAGE	RT oligos	GCGTTATG
	NPUIRO-67	ACGCGCAAGCAGAAGACGGCATACGAGATNNNNNNNNNNGGCATTGTGTGACTGGAGTTCAGACGTGTGCTCTTCCGATCTTTTTTTTTTTTTTTTTTTT		PAGE	RT oligos	ACAATGCC
	NPUIRO-68	ACGCGCAAGCAGAAGACGGCATACGAGATNNNNNNNNNNCCTCTTGTGTGACTGGAGTTCAGACGTGTGCTCTTCCGATCTTTTTTTTTTTTTTTTTTTT		PAGE	RT oligos	ACAAGAGG
	NPUIRO-69	ACGCGCAAGCAGAAGACGGCATACGAGATNNNNNNNNNNCATCCTCAGTGACTGGAGTTCAGACGTGTGCTCTTCCGATCTTTTTTTTTTTTTTTTTTTT		PAGE	RT oligos	TGAGGATG
	NPUIRO-70	ACGCGCAAGCAGAAGACGGCATACGAGATNNNNNNNNNNTGTGGCATGTGACTGGAGTTCAGACGTGTGCTCTTCCGATCTTTTTTTTTTTTTTTTTTTT		PAGE	RT oligos	ATGCCACA
	NPUIRO-71	ACGCGCAAGCAGAAGACGGCATACGAGATNNNNNNNNNNAGGTCTTGGTGACTGGAGTTCAGACGTGTGCTCTTCCGATCTTTTTTTTTTTTTTTTTTTT		PAGE	RT oligos	CAAGACCT
	NPUIRO-72	ACGCGCAAGCAGAAGACGGCATACGAGATNNNNNNNNNNAGACCGTAGTGACTGGAGTTCAGACGTGTGCTCTTCCGATCTTTTTTTTTTTTTTTTTTTT		PAGE	RT oligos	TACGGTCT
	NPUIRO-73	ACGCGCAAGCAGAAGACGGCATACGAGATNNNNNNNNNNTACGGTGTGTGACTGGAGTTCAGACGTGTGCTCTTCCGATCTTTTTTTTTTTTTTTTTTTT		PAGE	RT oligos	ACACCGTA
	NPUIRO-74	ACGCGCAAGCAGAAGACGGCATACGAGATNNNNNNNNNNTACCGATCGTGACTGGAGTTCAGACGTGTGCTCTTCCGATCTTTTTTTTTTTTTTTTTTTT		PAGE	RT oligos	GATCGGTA
	NPUIRO-75	ACGCGCAAGCAGAAGACGGCATACGAGATNNNNNNNNNNCTGAGTGTGTGACTGGAGTTCAGACGTGTGCTCTTCCGATCTTTTTTTTTTTTTTTTTTTT		PAGE	RT oligos	ACACTCAG
	NPUIRO-76	ACGCGCAAGCAGAAGACGGCATACGAGATNNNNNNNNNNAGTGCTGTGTGACTGGAGTTCAGACGTGTGCTCTTCCGATCTTTTTTTTTTTTTTTTTTTT		PAGE	RT oligos	ACAGCACT
	NPUIRO-77	ACGCGCAAGCAGAAGACGGCATACGAGATNNNNNNNNNNTCTTGTCGGTGACTGGAGTTCAGACGTGTGCTCTTCCGATCTTTTTTTTTTTTTTTTTTTT		PAGE	RT oligos	CGACAAGA
	NPUIRO-78	ACGCGCAAGCAGAAGACGGCATACGAGATNNNNNNNNNNGATTCGGTGTGACTGGAGTTCAGACGTGTGCTCTTCCGATCTTTTTTTTTTTTTTTTTTTT		PAGE	RT oligos	ACCGAATC
	NPUIRO-79	ACGCGCAAGCAGAAGACGGCATACGAGATNNNNNNNNNNTTGCTTGCGTGACTGGAGTTCAGACGTGTGCTCTTCCGATCTTTTTTTTTTTTTTTTTTTT		PAGE	RT oligos	GCAAGCAA
	NPUIRO-80	ACGCGCAAGCAGAAGACGGCATACGAGATNNNNNNNNNNCCACTAAGGTGACTGGAGTTCAGACGTGTGCTCTTCCGATCTTTTTTTTTTTTTTTTTTTT		PAGE	RT oligos	CTTAGTGG
	NPUIRO-81	ACGCGCAAGCAGAAGACGGCATACGAGATNNNNNNNNNNTGCTAGGTGTGACTGGAGTTCAGACGTGTGCTCTTCCGATCTTTTTTTTTTTTTTTTTTTT		PAGE	RT oligos	ACCTAGCA
	NPUIRO-82	ACGCGCAAGCAGAAGACGGCATACGAGATNNNNNNNNNNCAGCCAATGTGACTGGAGTTCAGACGTGTGCTCTTCCGATCTTTTTTTTTTTTTTTTTTTT		PAGE	RT oligos	ATTGGCTG
	NPUIRO-83	ACGCGCAAGCAGAAGACGGCATACGAGATNNNNNNNNNNGAATGCGAGTGACTGGAGTTCAGACGTGTGCTCTTCCGATCTTTTTTTTTTTTTTTTTTTT		PAGE	RT oligos	TCGCATTC
	NPUIRO-84	ACGCGCAAGCAGAAGACGGCATACGAGATNNNNNNNNNNCAACAGGTGTGACTGGAGTTCAGACGTGTGCTCTTCCGATCTTTTTTTTTTTTTTTTTTTT		PAGE	RT oligos	ACCTGTTG
	NPUIRO-85	ACGCGCAAGCAGAAGACGGCATACGAGATNNNNNNNNNNGGTAAGCTGTGACTGGAGTTCAGACGTGTGCTCTTCCGATCTTTTTTTTTTTTTTTTTTTT		PAGE	RT oligos	AGCTTACC
	NPUIRO-86	ACGCGCAAGCAGAAGACGGCATACGAGATNNNNNNNNNNACAAGCGTGTGACTGGAGTTCAGACGTGTGCTCTTCCGATCTTTTTTTTTTTTTTTTTTTT		PAGE	RT oligos	ACGCTTGT
	NPUIRO-87	ACGCGCAAGCAGAAGACGGCATACGAGATNNNNNNNNNNTGGTGTACGTGACTGGAGTTCAGACGTGTGCTCTTCCGATCTTTTTTTTTTTTTTTTTTTT		PAGE	RT oligos	GTACACCA
	NPUIRO-88	ACGCGCAAGCAGAAGACGGCATACGAGATNNNNNNNNNNCTAGGTTGGTGACTGGAGTTCAGACGTGTGCTCTTCCGATCTTTTTTTTTTTTTTTTTTTT		PAGE	RT oligos	CAACCTAG
	NPUIRO-89	ACGCGCAAGCAGAAGACGGCATACGAGATNNNNNNNNNNGTTGACGTGTGACTGGAGTTCAGACGTGTGCTCTTCCGATCTTTTTTTTTTTTTTTTTTTT		PAGE	RT oligos	ACGTCAAC
	NPUIRO-90	ACGCGCAAGCAGAAGACGGCATACGAGATNNNNNNNNNNCGAACTCTGTGACTGGAGTTCAGACGTGTGCTCTTCCGATCTTTTTTTTTTTTTTTTTTTT		PAGE	RT oligos	AGAGTTCG
	NPUIRO-91	ACGCGCAAGCAGAAGACGGCATACGAGATNNNNNNNNNNTGGAAGTGGTGACTGGAGTTCAGACGTGTGCTCTTCCGATCTTTTTTTTTTTTTTTTTTTT		PAGE	RT oligos	CACTTCCA
	NPUIRO-92	ACGCGCAAGCAGAAGACGGCATACGAGATNNNNNNNNNNGACAATCCGTGACTGGAGTTCAGACGTGTGCTCTTCCGATCTTTTTTTTTTTTTTTTTTTT		PAGE	RT oligos	GGATTGTC
	NPUIRO-93	ACGCGCAAGCAGAAGACGGCATACGAGATNNNNNNNNNNGAGCTTAGGTGACTGGAGTTCAGACGTGTGCTCTTCCGATCTTTTTTTTTTTTTTTTTTTT		PAGE	RT oligos	CTAAGCTC
	NPUIRO-94	ACGCGCAAGCAGAAGACGGCATACGAGATNNNNNNNNNNCTCTACCTGTGACTGGAGTTCAGACGTGTGCTCTTCCGATCTTTTTTTTTTTTTTTTTTTT		PAGE	RT oligos	AGGTAGAG
	NPUIRO-95	ACGCGCAAGCAGAAGACGGCATACGAGATNNNNNNNNNNTTAGCGGTGTGACTGGAGTTCAGACGTGTGCTCTTCCGATCTTTTTTTTTTTTTTTTTTTT		PAGE	RT oligos	ACCGCTAA
	NPUIRO-96	ACGCGCAAGCAGAAGACGGCATACGAGATNNNNNNNNNNCGAATAGCGTGACTGGAGTTCAGACGTGTGCTCTTCCGATCTTTTTTTTTTTTTTTTTTTT		PAGE	RT oligos	GCTATTCG
	NPUIRO-97	ACGCGCAAGCAGAAGACGGCATACGAGATNNNNNNNNNNGCCAACATGTGACTGGAGTTCAGACGTGTGCTCTTCCGATCTTTTTTTTTTTTTTTTTTTT		PAGE	RT oligos	ATGTTGGC
	NPUIRO-98	ACGCGCAAGCAGAAGACGGCATACGAGATNNNNNNNNNNTTAAGGCGGTGACTGGAGTTCAGACGTGTGCTCTTCCGATCTTTTTTTTTTTTTTTTTTTT		PAGE	RT oligos	CGCCTTAA
	NPUIRO-99	ACGCGCAAGCAGAAGACGGCATACGAGATNNNNNNNNNNTGGTTCGAGTGACTGGAGTTCAGACGTGTGCTCTTCCGATCTTTTTTTTTTTTTTTTTTTT		PAGE	RT oligos	TCGAACCA
	NPUIRO-100	ACGCGCAAGCAGAAGACGGCATACGAGATNNNNNNNNNNATCGTTGGGTGACTGGAGTTCAGACGTGTGCTCTTCCGATCTTTTTTTTTTTTTTTTTTTT		PAGE	RT oligos	CCAACGAT
	NPUIRO-101	ACGCGCAAGCAGAAGACGGCATACGAGATNNNNNNNNNNGACTAGTGGTGACTGGAGTTCAGACGTGTGCTCTTCCGATCTTTTTTTTTTTTTTTTTTTT		PAGE	RT oligos	CACTAGTC
	NPUIRO-102	ACGCGCAAGCAGAAGACGGCATACGAGATNNNNNNNNNNCTCCATAGGTGACTGGAGTTCAGACGTGTGCTCTTCCGATCTTTTTTTTTTTTTTTTTTTT		PAGE	RT oligos	CTATGGAG
	NPUIRO-103	ACGCGCAAGCAGAAGACGGCATACGAGATNNNNNNNNNNTGCCTCTTGTGACTGGAGTTCAGACGTGTGCTCTTCCGATCTTTTTTTTTTTTTTTTTTTT		PAGE	RT oligos	AAGAGGCA
	NPUIRO-104	ACGCGCAAGCAGAAGACGGCATACGAGATNNNNNNNNNNAGGCGATTGTGACTGGAGTTCAGACGTGTGCTCTTCCGATCTTTTTTTTTTTTTTTTTTTT		PAGE	RT oligos	AATCGCCT
	NPUIRO-105	ACGCGCAAGCAGAAGACGGCATACGAGATNNNNNNNNNNCCGGATATGTGACTGGAGTTCAGACGTGTGCTCTTCCGATCTTTTTTTTTTTTTTTTTTTT		PAGE	RT oligos	ATATCCGG
	NPUIRO-106	ACGCGCAAGCAGAAGACGGCATACGAGATNNNNNNNNNNATCACGAGGTGACTGGAGTTCAGACGTGTGCTCTTCCGATCTTTTTTTTTTTTTTTTTTTT		PAGE	RT oligos	CTCGTGAT

*1 Blue and red indicate the UMI (10 nt) and multiplex index (8 nt) regions, respectively.

*2 Index sequences indicate the sequences of the multiplex index (red) regions in the RT oligos NPUIROXX, which are reverse-complementally described in the RT oligos.

Supplementary Table S2. Statistics of pilot sequencing for 1cell-DGE.

Sample name	Total read counts	Mapped read counts	Mapping rate (%)*1	UMI counts	UMI-unified rate (%)*2	Read complexity	Number of detected genes
5 μg_a	811,701	731,872	90.2	680,993	6.95	0.5851034	15,559
5 μg_b	1,018,468	908,301	89.2	832,593	8.34	0.5479819	15,609
20 pg_c	686,473	520,680	75.8	82,229	84.21	0.6436713	7,760
20 pg_d	744,080	569,578	76.5	101,533	82.17	0.6467934	8,539

*1 Mapping rate (%) =Mapped read counts/Total read counts*100
*2 UMI-uniffied rate (%) =(1-UMI counts/Mapped read counts)*100

Supplementary Table S3. NGS statistics of 1cell-DGE data derived from individual leaf cells at 0 h and 24 h after leaf excision.

Sample name	Time after excision	Total read counts*1	Mapped read counts*1	Mapping rate (%)*2	UMI counts*1	UMI-unified rate (%)*3	Read complexity*1	Number of detected genes*1	MQS*1	WCQS*1	QC*1	Pseudotime *4	Time of sampling (min) *5
Pp0h07	0_h	5,954,822	5,352,911	89.89	100,716	98.12	0.2136	5,084	0.5000	0.2253	PASS	2.48447053	8
Pp0h08	0_h	7,407,268	6,735,197	90.93	94,890	98.59	0.1502	4,814	0.0313	-1.3726	PASS	2.74214655	15
Pp0h11	0_h	5,785,703	5,394,772	93.24	90,606	98.32	0.2049	5,119	0.2813	-0.6939	PASS	2.5444434	16
Pp0h15	0_h	7,772,103	7,331,723	94.33	128,778	98.24	0.1798	5,517	0.1875	0.8134	PASS	3.56734777	5
Pp0h16	0_h	5,948,917	5,570,477	93.64	106,549	98.09	0.2196	5,324	0.5938	0.6939	PASS	3.78880981	7
Pp0h17	0_h	4,351,177	3,903,907	89.72	75,954	98.05	0.1970	4,312	0.0625	-0.8004	PASS	0	13
Pp0h18	0_h	8,358,056	7,971,634	95.38	138,918	98.26	0.2042	6,119	0.4063	1.1402	PASS	5.16659546	25
Pp0h19	0_h	7,599,425	7,204,276	94.80	139,785	98.06	0.1534	5,100	0.0625	6.8238	PASS	1.10847502	35
Pp0h20	0_h	6,646,175	6,227,928	93.71	121,069	98.06	0.2449	6,322	0.8438	1.4283	PASS	7.84721727	10
Pp0h21	0_h	6,593,975	6,043,271	91.65	101,008	98.33	0.1712	5,038	0.1250	-0.4936	PASS	0.30883093	16
Pp0h22	0_h	7,434,514	6,522,729	87.74	102,971	98.42	0.1853	5,231	0.0625	-0.9171	PASS	1.52402136	10
Pp0h23	0_h	7,754,758	7,060,444	91.05	139,180	98.03	0.1539	5,273	0.0938	1.7941	PASS	0.99759627	15
Pp0h25	0_h	6,139,396	5,793,091	94.36	90,999	98.43	0.1767	4,673	0.0938	-1.2776	PASS	1.64859071	10
Pp0h26	0_h	5,679,409	5,318,467	93.64	112,453	97.89	0.2183	5,171	0.5313	1.8662	PASS	3.97365972	6
Pp0h27	0_h	5,418,549	5,016,331	92.58	81,728	98.37	0.2262	5,112	0.0938	-1.6461	PASS	2.09152675	5
Pp0h29	0_h	5,224,056	4,823,249	92.33	92,534	98.08	0.2302	5,100	0.4063	0.1779	PASS	3.16121858	10
Pp0h31	0_h	5,384,564	4,973,552	92.37	85,179	98.29	0.2423	5,268	0.1563	-0.9395	PASS	1.83612164	9
Pp0h32	0_h	5,980,255	5,197,727	86.91	91,303	98.24	0.2186	4,746	0.1875	-0.8526	PASS	0.51736406	15
Pp0h33	0_h	5,977,933	5,444,162	91.07	92,872	98.29	0.2089	4,764	0.2813	-0.5207	PASS	0.12829517	7
Pp0h35	0_h	4,448,860	3,702,867	83.23	72,508	98.04	0.1955	4,396	0.0313	-1.4849	PASS	1.46284217	5
Pp0h36	0_h	5,326,317	4,993,763	93.76	87,311	98.25	0.1900	4,269	0.2188	-0.6045	PASS	1.99624637	6
Pp0h37	0_h	6,620,571	6,224,418	94.02	108,166	98.26	0.2333	5,542	0.4375	0.2983	PASS	4.43122129	15
Pp0h38	0_h	5,719,150	5,108,156	89.32	107,241	97.90	0.2338	5,794	0.6875	1.2776	PASS	4.23069832	3
Pp0h40	0_h	6,585,847	6,042,093	91.74	101,351	98.32	0.1998	5,109	0.2500	-0.3657	PASS	1.3118313	7
Pp0h41	0_h	6,105,946	5,610,491	91.89	103,082	98.16	0.2616	6,123	0.5938	0.3930	PASS	4.33470401	20
Pp0h42	0_h	6,801,120	6,253,349	91.95	113,409	98.19	0.2506	6,156	0.5625	0.7385	PASS	4.54965935	10
Pp0h43	0_h	4,428,779	3,715,430	83.89	85,599	97.70	0.2449	5,215	0.1875	0.4575	PASS	3.32755617	20
Pp0h44	0_h	4,839,801	4,141,097	85.56	87,708	97.88	0.2380	5,383	0.2500	0.1504	PASS	2.9918295	13
Pp0h45	0_h	5,523,510	4,798,241	86.87	82,766	98.28	0.2201	4,777	0.1250	-1.6268	Artifact	NA*6	6
Pp0h46	0_h	5,819,209	5,351,508	91.96	91,017	98.30	0.2036	5,088	0.3125	-0.6301	PASS	0.69530128	4
Pp0h49	0_h	5,997,739	5,419,559	90.36	108,542	98.00	0.2529	5,637	0.7500	1.0259	PASS	2.37180176	11
Pp0h50	0_h	5,800,228	4,873,575	84.02	132,454	97.28	0.2766	7,293	0.9063	6.9424	PASS	5.72267048	10
Pp24h07	24_h	5,637,469	5,158,813	91.51	129,229	97.49	0.3737	10,169	0.9412	3.0852	PASS	17.6239062	NA
Pp24h08	24_h	5,412,246	5,137,579	94.93	103,992	97.98	0.3361	8,440	0.7059	1.1496	PASS	15.7930249	NA
Pp24h09	24_h	6,700,204	6,214,444	92.75	105,335	98.30	0.2426	7,737	0.1765	-0.4323	PASS	12.4691269	NA
Pp24h10	24_h	6,955,776	6,485,000	93.23	109,375	98.31	0.2693	8,317	0.3235	0.0770	PASS	11.3746733	NA
Pp24h11	24_h	3,536,190	3,322,301	93.95	85,305	97.43	0.4343	9,365	0.3529	9.5971	PASS	14.6051886	NA
Pp24h13	24_h	6,381,293	5,898,403	92.43	99,194	98.32	0.2340	7,329	0.1471	-0.7579	PASS	13.3059179	NA
Pp24h14	24_h	6,404,859	6,163,418	96.23	120,358	98.05	0.3204	8,709	0.6765	1.5308	PASS	16.9081286	NA
Pp24h15	24_h	6,139,061	5,680,420	92.53	117,363	97.93	0.2799	8,066	0.4118	1.0682	PASS	11.6626954	NA
Pp24h16	24_h	4,371,492	4,098,002	93.74	93,631	97.72	0.3579	7,944	0.5882	1.5944	PASS	14.9261596	NA
Pp24h19	24_h	5,940,847	5,395,415	90.82	96,442	98.21	0.1921	5,815	0.0294	-1.0469	PASS	15.5417205	NA
Pp24h20	24_h	5,167,780	4,684,846	90.65	88,824	98.10	0.2743	6,833	0.3529	-0.0451	PASS	13.1846612	NA
Pp24h22	24_h	6,900,372	6,628,448	96.06	106,528	98.39	0.3109	7,873	0.2059	0.0854	PASS	13.6321724	NA
Pp24h27	24_h	6,370,330	5,945,528	93.33	108,564	98.17	0.2949	8,754	0.5000	0.5789	PASS	15.4119804	NA
Pp24h28	24_h	6,064,551	5,632,967	92.88	105,262	98.13	0.2226	6,336	0.0882	-0.1994	PASS	11.6526744	NA
Pp24h29	24_h	4,627,333	4,337,578	93.74	73,001	98.32	0.3026	6,688	0.2059	-0.6492	PASS	12.695767	NA
Pp24h30	24_h	5,769,691	5,353,379	92.78	67,330	98.74	0.1993	5,694	0.0294	-2.7747	PASS	15.0677204	NA
Pp24h31	24_h	5,735,563	5,399,589	94.14	85,695	98.41	0.3165	7,612	0.1765	-0.4912	PASS	12.7990674	NA
Pp24h32	24_h	4,726,093	4,253,163	89.99	79,261	98.14	0.2653	6,304	0.2941	-0.6251	PASS	12.1373764	NA
Pp24h33	24_h	5,585,890	5,248,806	93.97	74,063	98.59	0.2641	6,297	0.0588	-1.6296	PASS	16.0629905	NA
Pp24h34	24_h	4,183,839	3,910,266	93.46	71,906	98.16	0.3422	6,900	0.1765	0.0090	PASS	13.0661535	NA
Pp24h35	24_h	7,228,019	6,922,708	95.78	150,351	97.83	0.3583	10,502	0.8824	6.3731	PASS	16.4137836	NA
Pp24h36	24_h	5,752,388	5,503,160	95.67	111,412	97.98	0.3497	8,353	0.8235	1.7578	PASS	11.3138157	NA
Pp24h37	24_h	5,307,976	5,046,770	95.08	73,129	98.55	0.2897	6,296	0.0882	-1.2824	PASS	15.3144368	NA
Pp24h38	24_h	3,830,515	3,576,402	93.37	87,735	97.55	0.3784	8,060	0.4412	1.9089	PASS	12.0447304	NA
Pp24h39	24_h	5,064,087	4,782,428	94.44	70,021	98.54	0.2428	5,192	0.1176	-1.8443	PASS	15.2567094	NA
Pp24h40	24_h	3,597,255	3,429,979	95.35	53,159	98.45	0.2925	4,842	0.0588	-1.5517	PASS	14.4899341	NA
Pp24h42	24_h	5,293,968	5,051,496	95.42	88,140	98.26	0.3144	7,025	0.4706	0.0876	PASS	10.8349512	NA
Pp24h44	24_h	5,256,026	4,629,337	88.08	85,905	98.14	0.2779	6,331	0.3824	-0.3870	PASS	10.2991898	NA
Pp24h45	24_h	5,797,433	5,270,294	90.91	93,454	98.23	0.2551	6,629	0.2353	-0.5039	PASS	13.8168923	NA
Pp24h46	24_h	6,196,766	5,056,623	81.60	96,521	98.09	0.2233	6,980	0.1176	-0.8304	PASS	11.1787643	NA
Pp24h47	24_h	5,386,771	4,953,570	91.96	92,026	98.14	0.3275	7,375	0.5294	0.4406	PASS	11.5058681	NA
Pp24h48	24_h	4,534,599	4,245,631	93.63	79,841	98.12	0.3482	7,451	0.3235	0.4739	PASS	13.9156131	NA
Pp24h49	24_h	2,738,547	2,207,352	80.60	49,092	97.78	0.3119	5,017	0.0294	-0.6496	PASS	15.6263512	NA
Pp24h50	24_h	3,471,852	3,226,300	92.93	71,489	97.78	0.3539	6,865	0.1471	0.6057	PASS	14.2214337	NA

*1 These statistics were calculated by SinQC.

*2 Mapping rate (%) =Mapped read counts/Total read counts*100

*3 UMI-uniffied rate (%) =(1-UMI counts/Mapped read counts)*100

*4 Pseudotime was caluculated using monocle package v1.4.0.

*5 Time of sampling indicates the time interval from the leaf cut to the extraction of the cell contents.

*6 Using SINQC program, Pp0h45 was discriminated as artifact and omitted to furthter analyses.

Supplementary Table S4. DEGs in 24 h-high identified using 1cell-DGE categorized by the GO term "cell growth".

Pp3.3 gene*	Best hit At gene*	At_symbol*	At_description*
Pp3c1_6150	AT2G35110	GRL.NAP1.NAPP	transcription activators
Pp3c1_37980	AT4G28250	ATEXPB3.ATHEXP BETA 1.6.EXPB3	expansin B3
Pp3c2_28200	AT4G26690	GPDL2.MRH5.SHV3	PLC-like phosphodiesterase family protein
Pp3c3_24130	AT5G55540	LOP1.TRN1	tornado 1
Pp3c3_31010	AT3G51460	RHD4	Phosphoinositide phosphatase family protein
Pp3c3_32980	AT3G07880	SCN1	Immunoglobulin E-set superfamily protein
Pp3c3_33440	AT5G09810	ACT7	actin 7
Pp3c4_1720	AT4G34490	ATCAP1.CAP 1.CAP1	cyclase associated protein 1
Pp3c4_6230	AT5G09810	ACT7	actin 7
Pp3c4_12350	AT1G75500	WAT1	Walls Are Thin 1
Pp3c5_2890	AT1G03060	SPI	Beige/BEACH domain ;WD domain. G-beta repeat protein
Pp3c5_6270	AT5G19530	ACL5	S-adenosyl-L-methionine-dependent methyltransferases superfamily protein
Pp3c6_5706	AT1G03060	SPI	Beige/BEACH domain ;WD domain. G-beta repeat protein
Pp3c6_6290	AT5G18580	EMB40.FASS.FASS 2.FS1.GDO.TON2	tonneau 2 (TON2)
Pp3c6_20680	AT4G38630	ATMCB1.MBP1.MCB1.RPN10	regulatory particle non-ATPase 10
Pp3c6_26100	AT1G03060	SPI	Beige/BEACH domain ;WD domain. G-beta repeat protein
Pp3c6_27380	AT5G19530	ACL5	S-adenosyl-L-methionine-dependent methyltransferases superfamily protein
Pp3c7_650	AT3G19820	CBB1.DIM.DIM1.DWF1.EVE1	cell elongation protein / DWARF1 / DIMINUTO (DIM)
Pp3c7_8710	AT5G04240	ELF6	Zinc finger (C2H2 type) family protein / transcription factor jumonji (jmj) family protein
Pp3c7_12810	AT5G56320	ATEXP14.ATEXPA14.ATHEXP ALPHA 1.5.EXP14.EXPA14	expansin A14
Pp3c7_12870	AT5G56320	ATEXP14.ATEXPA14.ATHEXP ALPHA 1.5.EXP14.EXPA14	expansin A14
Pp3c8_870	AT1G69530	AT-EXP1.ATEXP1.ATEXPA1.ATHEXP ALPHA 1.2.EXP1.EXPA1	expansin A1
Pp3c8_13450	AT1G69530	AT-EXP1.ATEXP1.ATEXPA1.ATHEXP ALPHA 1.2.EXP1.EXPA1	expansin A1
Pp3c8_13470	AT1G69530	AT-EXP1.ATEXP1.ATEXPA1.ATHEXP ALPHA 1.2.EXP1.EXPA1	expansin A1
Pp3c8_14900	AT5G56320	ATEXP14.ATEXPA14.ATHEXP ALPHA 1.5.EXP14.EXPA14	expansin A14
Pp3c8_25300	AT5G40770	ATPHB3.PHB3	prohibitin 3
Pp3c9_2830	AT5G26240	ATCLC-D.CLC-D	chloride channel D
Pp3c9_4143	AT2G13680	ATGSL02.CALS5.GLS2	callose synthase 5
Pp3c9_7720	AT2G06850	EXGT-A1.EXT.XTH4	xyloglucan endotransglucosylase/hydrolase 4
Pp3c10_5440	AT5G17020	ATCRM1.ATXPO1.HIT2.XPO1.XPO1A	exportin 1A
Pp3c9_11830	AT1G13980	EMB30.GN.VAN7	sec7 domain-containing protein
Pp3c10_17070	AT5G09810	ACT7	actin 7
Pp3c10_20720	AT5G55540	LOP1.TRN1	tornado 1
Pp3c11_1080	AT2G35110	GRL.NAP1.NAPP	transcription activators
Pp3c11_12000	AT5G56320	ATEXP14.ATEXPA14.ATHEXP ALPHA 1.5.EXP14.EXPA14	expansin A14
Pp3c11_26120	AT3G19820	CBB1.DIM.DIM1.DWF1.EVE1	cell elongation protein / DWARF1 / DIMINUTO (DIM)
Pp3c12_350	AT1G20330	CVP1.FRL1.SMT2	sterol methyltransferase 2
Pp3c12_4560	AT5G02260	ATEXP9.ATEXPA9.ATHEXP ALPHA 1.10.EXP9.EXPA9	expansin A9
Pp3c12_8200	AT3G54010	DEI1.PAS1	FKBP-type peptidyl-prolyl cis-trans isomerase family protein
Pp3c12_9050	AT3G62720	ATXT1.XT1.XXT1	xylosyltransferase 1
Pp3c12_23740	AT4G22910	CCS52A1.FZR2	FIZZY-related 2
Pp3c13_14220	AT1G12840	ATVHA-C.DET3	vacuolar ATP synthase subunit C (VATC) / V-ATPase C subunit (DET3)
Pp3c13_20360	AT5G02260	ATEXP9.ATEXPA9.ATHEXP ALPHA 1.10.EXP9.EXPA9	expansin A9
Pp3c14_1640	AT5G27540	emb2473.MIRO1	MIRO-related GTP-ase 1
Pp3c14_18440	AT5G56320	ATEXP14.ATEXPA14.ATHEXP ALPHA 1.5.EXP14.EXPA14	expansin A14
Pp3c14_18590	AT5G56320	ATEXP14.ATEXPA14.ATHEXP ALPHA 1.5.EXP14.EXPA14	expansin A14
Pp3c15_11320	AT1G13980	EMB30.GN.VAN7	sec7 domain-containing protein
Pp3c15_17790	AT3G57670	NTT.WIP2	C2H2-type zinc finger family protein
Pp3c16_16290	AT5G27540	emb2473.MIRO1	MIRO-related GTP-ase 1
Pp3c17_1680	AT1G20330	CVP1.FRL1.SMT2	sterol methyltransferase 2
Pp3c17_12980	AT4G28250	ATEXPB3.ATHEXP BETA 1.6.EXPB3	expansin B3
Pp3c17_20760	AT5G66410	PLP3b	phosducin-like protein 3 homolog
Pp3c18_19660	AT5G02260	ATEXP9.ATEXPA9.ATHEXP ALPHA 1.10.EXP9.EXPA9	expansin A9
Pp3c18_20160	AT5G23530	AtCXE18.CXE18	carboxyesterase 18
Pp3c18_20180	AT3G16640	TCTP	translationally controlled tumor protein
Pp3c19_4120	AT5G63840	PSL5.RSW3	Glycosyl hydrolases family 31 protein
Pp3c19_7110	AT3G61650	TUBG1	gamma-tubulin
Pp3c20_6790	AT4G27060	CN.SPR2.TOR1	ARM repeat superfamily protein
Pp3c20_15610	AT3G12160	ATRABA4D.RABA4D	RAB GTPase homolog A4D
Pp3c20_17290	AT5G54380	THE1	protein kinase family protein
Pp3c21_3680	AT3G16640	TCTP	translationally controlled tumor protein
Pp3c21_11720	AT3G13870	RHD3	Root hair defective 3 GTP-binding protein (RHD3)
Pp3c21_15710	AT1G74380	XXT5	xyloglucan xylosyltransferase 5
Pp3c22_630	AT4G28250	ATEXPB3.ATHEXP BETA 1.6.EXPB3	expansin B3
Pp3c22_2850	AT3G61650	TUBG1	gamma-tubulin
Pp3c22_23030	AT1G74380	XXT5	xyloglucan xylosyltransferase 5
Pp3c23_5320	AT5G26240	ATCLC-D.CLC-D	chloride channel D
Pp3c23_7840	AT3G12160	ATRABA4D.RABA4D	RAB GTPase homolog A4D
Pp3c24_6930	AT5G66680	DGL1	dolichyl-diphosphooligosaccharide-protein glycosyltransferase 48kDa subunit family protein
Pp3c24_11460	AT2G20190	ATCLASP.CLASP	CLIP-associated protein
Pp3c26_9700	AT1G49040	SCD1	stomatal cytokinesis defective / SCD1 protein (SCD1)
Pp3c26_14180	AT2G20370	KAM1.MUR3	Exostosin family protein
Pp3c27_1990	AT1G04950	ATTAF6.TAF6.TAFII59	TATA BOX ASSOCIATED FACTOR II 59
Pp3c27_3260	AT5G19530	ACL5	S-adenosyl-L-methionine-dependent methyltransferases superfamily protein
Pp3c27_8050	AT4G33880	RSL2	ROOT HAIR DEFECTIVE 6-LIKE 2

*Fifteen of 75 genes are colored in red as the common DEGs both 1cell-DGE and 5'DGE.

Supplementary Table S5. DEGs in 24 h-high identified using 1cell-DGE categorized by the GO term "cell cycle".

Pp3.3 gene*	Best hit At gene*	At_symbol*	At_description*
Pp3c1_6160	AT4G00100	ATRPS13A.PFL2.RPS13.RPS13A	ribosomal protein S13A
Pp3c2_6200	AT2G27970	CKS2	CDK-subunit 2
Pp3c2_13760	AT5G35520	ATMIS12.MIS12	minichromosome instability 12 (mis12)-like
Pp3c2_16690	AT5G18700	EMB3013.RUK	Protein kinase family protein with ARM repeat domain
Pp3c3_3110	AT5G05970	NEDD1	Transducin/WD40 repeat-like superfamily protein
Pp3c4_13590	AT5G03415	ATDPB.DPB	Transcription factor DP
Pp3c4_17856	AT3G07160	ATGSL10.CALS9.gsl10	glucan synthase-like 10
Pp3c4_25200	AT5G45610	SUV2	protein dimerizations
Pp3c6_3460	AT5G40820	ATATR.ATR.ATRAD3	Ataxia telangiectasia-mutated and RAD3-related
Pp3c6_6440	AT3G18730	BRU1.MGO3.TSK	tetratricopeptide repeat (TPR)-containing protein
Pp3c6_25960	AT1G50240	FU	Protein kinase family protein with ARM repeat domain
Pp3c7_9140	AT3G63130	ATRANGAP1.RANGAP1	RAN GTPase activating protein 1
Pp3c7_19554	AT4G14150	KINESIN-12A.PAKRP1	phragmoplast-associated kinesin-related protein 1
Pp3c7_26720	AT4G00100	ATRPS13A.PFL2.RPS13.RPS13A	ribosomal protein S13A
Pp3c9_2440	AT3G12280	ATRBR1.RB.RB1.RBR.RBR1	retinoblastoma-related 1
Pp3c9_4143	AT2G13680	ATGSL02.CALS5.GLS2	callose synthase 5
Pp3c9_8300	AT1G70210	ATCYCD1;1.CYCD1;1	CYCLIN D1;1
Pp3c9_11830	AT1G13980	EMB30.GN.VAN7	sec7 domain-containing protein
Pp3c9_15980	AT1G48270	GCR1	G-protein-coupled receptor 1
Pp3c10_19330	AT3G07160	ATGSL10.CALS9.gsl10	glucan synthase-like 10
Pp3c10_20867	AT3G07160	ATGSL10.CALS9.gsl10	glucan synthase-like 10
Pp3c11_1100	AT4G00100	ATRPS13A.PFL2.RPS13.RPS13A	ribosomal protein S13A
Pp3c11_16930	AT3G63130	ATRANGAP1.RANGAP1	RAN GTPase activating protein 1
Pp3c12_16340	AT5G07280	EMS1.EXS	Leucine-rich repeat transmembrane protein kinase
Pp3c12_23740	AT4G22910	CCS52A1.FZR2	FIZZY-related 2
Pp3c13_19570	AT1G77620	NaN	P-loop containing nucleoside triphosphate hydrolases superfamily protein
Pp3c14_1940	AT3G55340	PHIP1	phragmoplastin interacting protein 1
Pp3c14_3850	AT5G18700	EMB3013.RUK	Protein kinase family protein with ARM repeat domain
Pp3c14_18020	AT1G75950	ASK1.ATSKP1.SKP1.SKP1A.UIP1	S phase kinase-associated protein 1
Pp3c15_11320	AT1G13980	EMB30.GN.VAN7	sec7 domain-containing protein
Pp3c15_14600	AT5G15540	ATSCC2.EMB2773.SCC2	PHD finger family protein
Pp3c15_17470	AT1G70210	ATCYCD1;1.CYCD1;1	CYCLIN D1;1
Pp3c16_8790	AT1G78770	APC6	anaphase promoting complex 6
Pp3c16_15220	AT4G00100	ATRPS13A.PFL2.RPS13.RPS13A	ribosomal protein S13A
Pp3c19_7110	AT3G61650	TUBG1	gamma-tubulin
Pp3c21_1460	AT1G71230	AJH2.CSN5.CSN5B	COP9-signalosome 5B
Pp3c21_8640	AT2G40550	ETG1	E2F target gene 1
Pp3c22_2850	AT3G61650	TUBG1	gamma-tubulin
Pp3c24_4940	AT5G15920	SMC5	structural maintenance of chromosomes 5
Pp3c24_11460	AT2G20190	ATCLASP.CLASP	CLIP-associated protein
Pp3c26_9700	AT1G49040	SCD1	stomatal cytokinesis defective / SCD1 protein (SCD1)

*Two of 41 genes are colored in red as the common DEGs both 1cell-DGE and 5'DGE.

Supplementary Table S6. DEGs in 24 h-high identified using 1cell-DGE categorized by the GO term "cell differentiation".

Pp3.3 gene*	Best hit At gene*	At_symbol*	At_description*
Pp3c1_6150	AT2G35110	GRL.NAP1.NAPP	transcription activators
Pp3c1_6160	AT4G00100	ATRPS13A.PFL2.RPS13.RPS13A	ribosomal protein S13A
Pp3c1_6880	AT5G22220	ATE2FB.E2F1.E2FB	E2F transcription factor 1
Pp3c1_13010	AT5G45190	NaN	Cyclin family protein
Pp3c1_39840	AT2G38440	ATSCAR2.DIS3.ITB1.SCAR2.WAVE4	SCAR homolog 2
Pp3c2_5380	AT5G23630	MIA.PDR2	phosphate deficiency response 2
Pp3c2_8430	AT2G38440	ATSCAR2.DIS3.ITB1.SCAR2.WAVE4	SCAR homolog 2
Pp3c2_13410	AT5G10480	PAS2.PEP	Protein-tyrosine phosphatase-like. PTPLA
Pp3c2_28200	AT4G26690	GPDL2.MRH5.SHV3	PLC-like phosphodiesterase family protein
Pp3c2_34930	AT1G74030	ENO1	enolase 1
Pp3c3_31010	AT3G51460	RHD4	Phosphoinositide phosphatase family protein
Pp3c3_32980	AT3G07880	SCN1	Immunoglobulin E-set superfamily protein
Pp3c3_33440	AT5G09810	ACT7	actin 7
Pp3c4_6230	AT5G09810	ACT7	actin 7
Pp3c5_2890	AT1G03060	SPI	Beige/BEACH domain ;WD domain. G-beta repeat protein
Pp3c5_6270	AT5G19530	ACL5	S-adenosyl-L-methionine-dependent methyltransferases superfamily protein
Pp3c5_16690	AT2G01730	ATCPSF73-II.CPSF73-II.EDA26	cleavage and polyadenylation specificity factor 73 kDa subunit-II
Pp3c6_5706	AT1G03060	SPI	Beige/BEACH domain ;WD domain. G-beta repeat protein
Pp3c6_20680	AT4G38630	ATMCB1.MBP1.MCB1.RPN10	regulatory particle non-ATPase 10
Pp3c6_26100	AT1G03060	SPI	Beige/BEACH domain ;WD domain. G-beta repeat protein
Pp3c6_27380	AT5G19530	ACL5	S-adenosyl-L-methionine-dependent methyltransferases superfamily protein
Pp3c7_26720	AT4G00100	ATRPS13A.PFL2.RPS13.RPS13A	ribosomal protein S13A
Pp3c8_7540	AT1G72440	EDA25.SWA2	CCAAT-binding factor
Pp3c8_12030	AT5G02820	BIN5.RHL2	Spo11/DNA topoisomerase VI. subunit A protein
Pp3c9_2440	AT3G12280	ATRBR1.RB.RB1.RBR.RBR1	retinoblastoma-related 1
Pp3c9_4143	AT2G13680	ATGSL02.CALS5.GLS2	callose synthase 5
Pp3c9_11830	AT1G13980	EMB30.GN.VAN7	sec7 domain-containing protein
Pp3c9_23630	AT1G16890	UBC13B.UBC36	ubiquitin-conjugating enzyme 36
Pp3c10_4280	AT1G12860	ICE2.SCRM2	basic helix-loop-helix (bHLH) DNA-binding superfamily protein
Pp3c10_5440	AT5G17020	ATCRM1.ATXPO1.HIT2.XPO1.XPO1A	exportin 1A
Pp3c10_17070	AT5G09810	ACT7	actin 7
Pp3c11_1080	AT2G35110	GRL.NAP1.NAPP	transcription activators
Pp3c11_1100	AT4G00100	ATRPS13A.PFL2.RPS13.RPS13A	ribosomal protein S13A
Pp3c12_8200	AT3G54010	DEI1.PAS1	FKBP-type peptidyl-prolyl cis-trans isomerase family protein
Pp3c12_9050	AT3G62720	ATXT1.XT1.XXT1	xylosyltransferase 1
Pp3c12_16340	AT5G07280	EMS1.EXS	Leucine-rich repeat transmembrane protein kinase
Pp3c12_23740	AT4G22910	CCS52A1.FZR2	FIZZY-related 2
Pp3c13_13360	AT5G65700	BAM1	Leucine-rich receptor-like protein kinase family protein
Pp3c14_1640	AT5G27540	emb2473.MIRO1	MIRO-related GTP-ase 1
Pp3c14_15980	AT4G04890	PDF2	protodermal factor 2
Pp3c15_3730	AT5G65930	KCBP.PKCBP.ZWI	kinesin-like calmodulin-binding protein (ZWICHEL)
Pp3c15_11320	AT1G13980	EMB30.GN.VAN7	sec7 domain-containing protein
Pp3c15_17790	AT3G57670	NTT.WIP2	C2H2-type zinc finger family protein
Pp3c16_15220	AT4G00100	ATRPS13A.PFL2.RPS13.RPS13A	ribosomal protein S13A
Pp3c16_16290	AT5G27540	emb2473.MIRO1	MIRO-related GTP-ase 1
Pp3c17_16910	AT4G04890	PDF2	protodermal factor 2
Pp3c18_15140	AT4G24670	TAR2	tryptophan aminotransferase related 2
Pp3c18_19850	AT1G12860	ICE2.SCRM2	basic helix-loop-helix (bHLH) DNA-binding superfamily protein
Pp3c18_20160	AT5G23530	AtCXE18.CXE18	carboxyesterase 18
Pp3c18_20180	AT3G16640	TCTP	translationally controlled tumor protein
Pp3c19_4700	AT2G20490	EDA27.NOP10	nucleolar RNA-binding Nop10p family protein
Pp3c19_7110	AT3G61650	TUBG1	gamma-tubulin
Pp3c20_8320	AT5G48030	GFA2	gametophytic factor 2
Pp3c20_15610	AT3G12160	ATRABA4D.RABA4D	RAB GTPase homolog A4D
Pp3c21_530	AT5G64930	CPR5.HYS1	CPR5 protein. putative
Pp3c21_3680	AT3G16640	TCTP	translationally controlled tumor protein
Pp3c21_8020	AT5G18410	ATSRA1.KLK.PIR.PIR121.PIRP.SRA1	transcription activators
Pp3c21_11720	AT3G13870	RHD3	Root hair defective 3 GTP-binding protein (RHD3)
Pp3c21_15710	AT1G74380	XXT5	xyloglucan xylosyltransferase 5
Pp3c21_20720	AT4G13750	NOV	Histidine kinase-. DNA gyrase B-. and HSP90-like ATPase family protein
Pp3c22_2850	AT3G61650	TUBG1	gamma-tubulin
Pp3c22_5670	AT5G64930	CPR5.HYS1	CPR5 protein. putative
Pp3c22_5980	AT5G58230	ATMSI1.MEE70.MSI1	Transducin/WD40 repeat-like superfamily protein
Pp3c22_23030	AT1G74380	XXT5	xyloglucan xylosyltransferase 5
Pp3c23_7840	AT3G12160	ATRABA4D.RABA4D	RAB GTPase homolog A4D
Pp3c23_11160	AT2G19620	NDL3	N-MYC downregulated-like 3
Pp3c23_15370	AT5G48030	GFA2	gametophytic factor 2
Pp3c23_21700	AT2G19620	NDL3	N-MYC downregulated-like 3
Pp3c27_1990	AT1G04950	ATTAF6.TAF6.TAFII59	TATA BOX ASSOCIATED FACTOR II 59
Pp3c27_3260	AT5G19530	ACL5	S-adenosyl-L-methionine-dependent methyltransferases superfamily protein
Pp3c27_8050	AT4G33880	RSL2	ROOT HAIR DEFECTIVE 6-LIKE 2

*Three of 71 genes are colored in red as the common DEGs both 1cell-DGE and 5'DGE.

Supplementary Table S7. DEGs in 24 h-high identified using 1cell-DGE categorized by the GO term "embryonic development".

Pp3.3 gene*	Best hit At gene*	At_symbol*	At_description*
Pp3c1_4140	AT4G36800	RCE1	RUB1 conjugating enzyme 1
Pp3c1_7900	AT5G53400	BOB1	HSP20-like chaperones superfamily protein
Pp3c1_10040	AT4G01560	MEE49	Ribosomal RNA processing Brix domain protein
Pp3c1_19310	AT5G41480	ATDFA.DFA.EMB9.GLA1	Folylpolyglutamate synthetase family protein
Pp3c1_21660	AT5G09900	EMB2107.MSA.RPN5A	26S proteasome regulatory subunit. putative (RPN5)
Pp3c1_29840	AT5G22130	PNT1	mannosyltransferase family protein
Pp3c1_31160	AT2G26890	GRV2.KAM2	DNAJ heat shock N-terminal domain-containing protein
Pp3c1_32590	AT4G10760	EMB1706.MTA	mRNAadenosine methylase
Pp3c1_32740	AT5G53860	emb2737.EMB64	embryo defective 2737
Pp3c1_34100	AT5G37510	CI76.EMB1467	NADH-ubiquinone dehydrogenase. mitochondrial. putative
Pp3c1_34140	AT5G37510	CI76.EMB1467	NADH-ubiquinone dehydrogenase. mitochondrial. putative
Pp3c1_35400	AT5G27720	emb1644	Small nuclear ribonucleoprotein family protein
Pp3c1_35770	AT1G48410	AGO1	Stabilizer of iron transporter SufD / Polynucleotidyl transferase
Pp3c1_35880	AT1G20960	emb1507	U5 small nuclear ribonucleoprotein helicase. putative
Pp3c1_39460	AT4G04040	MEE51	Phosphofructokinase family protein
Pp3c1_40060	AT5G26742	emb1138	DEAD box RNA helicase (RH3)
Pp3c2_4060	AT1G48410	AGO1	Stabilizer of iron transporter SufD / Polynucleotidyl transferase
Pp3c2_9830	AT1G14610	TWN2.VALRS	valyl-tRNA synthetase / valine--tRNA ligase (VALRS)
Pp3c2_11980	AT4G04040	MEE51	Phosphofructokinase family protein
Pp3c2_13690	AT3G48930	EMB1080	Nucleic acid-binding. OB-fold-like protein
Pp3c2_16690	AT5G18700	EMB3013.RUK	Protein kinase family protein with ARM repeat domain
Pp3c2_18070	AT3G60740	CHO.EMB133.TFC D.TTN1	ARM repeat superfamily protein
Pp3c2_24840	AT4G31160	DCAF1	DDB1-CUL4 associated factor 1
Pp3c2_30250	AT3G55610	P5CS2	delta 1-pyrroline-5-carboxylate synthase 2
Pp3c3_270	AT5G57020	ATNMT1.NMT1	myristoyl-CoA:protein N-myristoyltransferase
Pp3c3_810	AT1G49770	RGE1.ZOU	basic helix-loop-helix (bHLH) DNA-binding superfamily protein
Pp3c3_5060	AT3G07060	emb1974	NHL domain-containing protein
Pp3c3_12210	AT2G18020	EMB2296	Ribosomal protein L2 family
Pp3c3_16200	AT2G26890	GRV2.KAM2	DNAJ heat shock N-terminal domain-containing protein
Pp3c3_25480	AT3G52590	EMB2167.ERD16.HAP4.UBQ1	ubiquitin extension protein 1
Pp3c3_29950	AT1G15220	ATCCMH.CCMH	cytochrome c biogenesis protein family
Pp3c3_37360	AT1G21690	EMB1968.RFC4	ATPase family associated with various cellular activities (AAA)
Pp3c4_7260	AT1G69780	ATHB13	Homeobox-leucine zipper protein family
Pp3c4_19910	AT2G47470	ATPDI11.ATPDIL2-1.MEE30.PDI11.UNE5	thioredoxin family protein
Pp3c4_20560	AT1G80410	EMB2753	tetratricopeptide repeat (TPR)-containing protein
Pp3c5_5900	AT4G09980	EMB1691	Methyltransferase MT-A70 family protein
Pp3c5_15520	AT4G15900	PRL1	pleiotropic regulatory locus 1
Pp3c5_20500	AT5G20920	EIF2 BETA.EMB1401	eukaryotic translation initiation factor 2 beta subunit
Pp3c5_22030	AT1G12980	DRN.ESR1	Integrase-type DNA-binding superfamily protein
Pp3c6_3890	AT4G26500	ATSUFE.CPSUFE.EMB1374.SUFE1	chloroplast sulfur E
Pp3c6_10010	AT3G46740	MAR1.TOC75-III	translocon at the outer envelope membrane of chloroplasts 75-III
Pp3c7_3950	AT2G43410	FPA	RNA binding
Pp3c7_4470	AT5G19820	emb2734	ARM repeat superfamily protein
Pp3c7_5560	AT2G39990	AteIF3f.EIF2.eIF3F	eukaryotic translation initiation factor 2
Pp3c7_5660	AT3G61140	ATFUS6.ATSK31.COP11.CSN1.EMB78.FUS6.SK31	26S proteasome. regulatory subunit Rpn7;Proteasome component (PCI) domain
Pp3c7_5780	AT5G10360	EMB3010.RPS6B	Ribosomal protein S6e
Pp3c7_18650	AT2G14680	MEE13	myosin heavy chain-related
Pp3c7_23430	AT3G46740	MAR1.TOC75-III	translocon at the outer envelope membrane of chloroplasts 75-III
Pp3c8_12060	AT2G18390	ARL2.ATARLC1.HAL.TTN5	ADP-ribosylation factor family protein
Pp3c8_13570	AT3G54420	ATCHITIV.ATEP3.CHIV.EP3	homolog of carrot EP3-3 chitinase
Pp3c8_15480	AT1G11680	CYP51.CYP51A2.CYP51G1.EMB1738	CYTOCHROME P450 51G1
Pp3c8_16130	AT3G04400	emb2171	Ribosomal protein L14p/L23e family protein
Pp3c8_16870	AT4G21800	QQT2	P-loop containing nucleoside triphosphate hydrolases superfamily protein
Pp3c8_21800	AT1G55900	emb1860.TIM50	Haloacid dehalogenase-like hydrolase (HAD) superfamily protein
Pp3c9_11830	AT1G13980	EMB30.GN.VAN7	sec7 domain-containing protein
Pp3c9_21250	AT1G80490	TPR1	TOPLESS-related 1
Pp3c9_21420	AT1G13120	emb1745	embryo defective 1745
Pp3c10_6570	AT4G23750	CRF2.TMO3	cytokinin response factor 2
Pp3c10_20750	AT1G80410	EMB2753	tetratricopeptide repeat (TPR)-containing protein
Pp3c10_25270	AT3G52590	EMB2167.ERD16.HAP4.UBQ1	ubiquitin extension protein 1
Pp3c11_1000	AT4G26300	emb1027	Arginyl-tRNA synthetase. class Ic
Pp3c11_19380	AT3G12080	emb2738	GTP-binding family protein
Pp3c11_21120	AT5G10360	EMB3010.RPS6B	Ribosomal protein S6e
Pp3c11_22120	AT5G19820	emb2734	ARM repeat superfamily protein
Pp3c12_7760	AT5G56680	EMB2755.SYNC1.SYNC1 ARATH	Class II aminoacyl-tRNA and biotin synthetases superfamily protein
Pp3c12_8200	AT3G54010	DEI1.PAS1	FKBP-type peptidyl-prolyl cis-trans isomerase family protein
Pp3c12_22440	AT2G04030	AtHsp90.5.CR88.EMB1956.Hsp88.1.HSP90.5	Chaperone protein htpG family protein
Pp3c13_1280	AT5G57020	ATNMT1.NMT1	myristoyl-CoA:protein N-myristoyltransferase
Pp3c13_15440	AT4G16280	FCA	RNA binding;abscisic acid binding
Pp3c13_20310	AT4G29660	EMB2752	embryo defective 2752
Pp3c13_24470	AT1G67730	ATKCR1.KCR1.YBR159	beta-ketoacyl reductase 1
Pp3c14_1640	AT5G27540	emb2473.MIRO1	MIRO-related GTP-ase 1
Pp3c14_3280	AT4G23750	CRF2.TMO3	cytokinin response factor 2
Pp3c14_3310	AT4G23750	CRF2.TMO3	cytokinin response factor 2
Pp3c14_3850	AT5G18700	EMB3013.RUK	Protein kinase family protein with ARM repeat domain
Pp3c14_9600	AT3G48930	EMB1080	Nucleic acid-binding. OB-fold-like protein
Pp3c14_11180	AT5G27720	emb1644	Small nuclear ribonucleoprotein family protein
Pp3c14_15980	AT4G04890	PDF2	protodermal factor 2
Pp3c14_21790	AT5G08170	ATAIH.EMB1873	porphyromonas-type peptidyl-arginine deiminase family protein
Pp3c15_8820	AT2G31530	EMB2289.SCY2	SecY protein transport family protein
Pp3c15_10750	AT2G45270	GCP1	glycoprotease 1
Pp3c15_11320	AT1G13980	EMB30.GN.VAN7	sec7 domain-containing protein
Pp3c15_14600	AT5G15540	ATSCC2.EMB2773.SCC2	PHD finger family protein
Pp3c15_25430	AT5G10360	EMB3010.RPS6B	Ribosomal protein S6e
Pp3c15_25780	AT5G10360	EMB3010.RPS6B	Ribosomal protein S6e
Pp3c16_13640	AT3G04400	emb2171	Ribosomal protein L14p/L23e family protein
Pp3c16_16290	AT5G27540	emb2473.MIRO1	MIRO-related GTP-ase 1
Pp3c16_16500	AT5G13480	FY	Transducin/WD40 repeat-like superfamily protein

Pp3.3 gene*	Best hit At gene*	At_symbol*	At_description*
Pp3c16_16780	AT2G31305	INH3	inhibitor-3
Pp3c17_13460	AT4G23660	AtPPT1.PPT1	polyprenyltransferase 1
Pp3c17_16910	AT4G04890	PDF2	protodermal factor 2
Pp3c17_17770	AT5G55940	emb2731	Uncharacterised protein family (UPF0172)
Pp3c17_20370	AT3G16290	EMB2083	AAA-type ATPase family protein
Pp3c18_11480	AT3G01610	CDC48C.emb1354	cell division cycle 48C
Pp3c18_12190	AT3G60830	ARP7.ATARP7	actin-related protein 7
Pp3c18_15140	AT4G24670	TAR2	tryptophan aminotransferase related 2
Pp3c18_20140	AT5G60540	ATPDX2.EMB2407.PDX2	pyridoxine biosynthesis 2
Pp3c19_9420	AT5G10330	ATHPA1.EMB2196.HISN6A.HPA1	histidinol phosphate aminotransferase 1
Pp3c19_12460	AT5G16750	TOZ	Transducin family protein / WD-40 repeat family protein
Pp3c19_14950	AT1G24590	DRN-LIKE.DRNL.ESR2.SOB2	DORNROSCHEN-like
Pp3c19_15000	AT2G04030	AtHsp90.5.CR88.EMB1956.Hsp88.1.HSP90.5	Chaperone protein htpG family protein
Pp3c19_17130	AT3G20630	ATUBP14.PER1.TTN6.UBP14	ubiquitin-specific protease 14
Pp3c19_19270	AT5G37510	CI76.EMB1467	NADH-ubiquinone dehydrogenase. mitochondrial. putative
Pp3c20_2050	AT1G07890	APX1.ATAPX01.ATAPX1.CS1.MEE6	ascorbate peroxidase 1
Pp3c20_6200	AT3G46560	emb2474.TIM9	Tim10/DDP family zinc finger protein
Pp3c20_9520	AT5G62440	NaN	Protein of unknown function (DUF3223)
Pp3c20_10110	AT1G55900	emb1860.TIM50	Haloacid dehalogenase-like hydrolase (HAD) superfamily protein
Pp3c20_12860	AT4G29860	EMB2757.TAN	Transducin/WD40 repeat-like superfamily protein
Pp3c20_23170	AT3G52590	EMB2167.ERD16.HAP4.UBQ1	ubiquitin extension protein 1
Pp3c21_1420	AT4G00231	MEE50	ARM repeat superfamily protein
Pp3c21_20720	AT4G13750	NOV	Histidine kinase-. DNA gyrase B-. and HSP90-like ATPase family protein
Pp3c22_5980	AT5G58230	ATMSI1.MEE70.MSI1	Transducin/WD40 repeat-like superfamily protein
Pp3c22_13190	AT1G01470	LEA14.LSR3	Late embryogenesis abundant protein
Pp3c23_4520	AT3G04400	emb2171	Ribosomal protein L14p/L23e family protein
Pp3c23_8340	AT1G63700	EMB71.MAPKKK4.YDA	Protein kinase superfamily protein
Pp3c23_13490	AT1G30610	EMB2279.EMB88	pentatricopeptide (PPR) repeat-containing protein
Pp3c23_17430	AT3G55620	emb1624	Translation initiation factor IF6
Pp3c24_430	AT3G52590	EMB2167.ERD16.HAP4.UBQ1	ubiquitin extension protein 1
Pp3c24_1830	AT2G26830	emb1187	Protein kinase superfamily protein
Pp3c24_6080	AT1G63700	EMB71.MAPKKK4.YDA	Protein kinase superfamily protein
Pp3c24_11510	AT1G49510	emb1273	embryo defective 1273
Pp3c24_12180	AT2G28000	CH-CPN60A.CPN60A.SLP	chaperonin-60alpha
Pp3c24_13910	AT3G55620	emb1624	Translation initiation factor IF6
Pp3c24_15930	AT3G46560	emb2474.TIM9	Tim10/DDP family zinc finger protein
Pp3c24_17310	AT5G24400	EMB2024.PGL3	NagB/RpiA/CoA transferase-like superfamily protein
Pp3c25_4250	AT1G12980	DRN.ESR1	Integrase-type DNA-binding superfamily protein
Pp3c25_5630	AT5G27740	EMB161.EMB251.EMB2775.RFC3	ATPase family associated with various cellular activities (AAA)
Pp3c26_2070	AT2G28880	ADCS.emb1997	para-aminobenzoate (PABA) synthase family protein
Pp3c27_190	AT5G20920	EIF2 BETA.EMB1401	eukaryotic translation initiation factor 2 beta subunit
Pp3c27_6340	AT1G06220	CLO.GFA1.MEE5	Ribosomal protein S5/Elongation factor G/III/V family protein
Pp3s116_30	AT3G48930	EMB1080	Nucleic acid-binding. OB-fold-like protein

*Nine of 131 genes are colored in red as the common DEGs both 1cell-DGE and 5'DGE.

Supplementary Table S8. DEGs in 24 h-high identified using 1cell-DGE categorized by the GO term "DNA metabolic process".

Pp3.3 gene*	Best hit At gene*	At_symbol*	At_description*
Pp3c1_23680	AT1G12244	NaN	Polynucleotidyl transferase. ribonuclease H-like superfamily protein
Pp3c1_32870	AT2G07690	MCM5	Minichromosome maintenance (MCM2/3/5) family protein
Pp3c1_34050	AT1G12400	NaN	Nucleotide excision repair. TFIIH. subunit TTDA
Pp3c1_36240	AT2G16390	CHR35.DMS1.DRD1	SNF2 domain-containing protein / helicase domain-containing protein
Pp3c1_36770	AT5G50340	NaN	ATP-dependent peptidases;nucleotide binding;serine-type endopeptidases;DNA helicases
Pp3c2_1760	AT1G60930	ATRECQ4B.RECQ4B.RECQL4B	RECQ helicase L4B
Pp3c2_11270	AT1G05180	AXR1	NAD(P)-binding Rossmann-fold superfamily protein
Pp3c2_14050	AT1G07270	NaN	Cell division control. Cdc6
Pp3c2_36940	AT3G47830	NaN	DNA glycosylase superfamily protein
Pp3c3_5880	AT2G19480	NAP1;2.NFA02.NFA2	nucleosome assembly protein 1;2
Pp3c4_13590	AT5G03415	ATDPB.DPB	Transcription factor DP
Pp3c4_16040	AT5G57970	NaN	DNA glycosylase superfamily protein
Pp3c5_3310	AT2G22140	ATEME1B.EME1B	essential meiotic endonuclease 1B
Pp3c5_3400	AT1G12370	PHR1.UVR2	photolyase 1
Pp3c5_4530	AT3G23580	RNR2.RNR2A	ribonucleotide reductase 2A
Pp3c5_12730	AT3G19210	ATRAD54.CHR25.RAD54	homolog of RAD54
Pp3c5_18670	AT3G26680	ATSNM1.SNM1	DNA repair metallo-beta-lactamase family protein
Pp3c6_3460	AT5G40820	ATATR.ATR.ATRAD3	Ataxia telangiectasia-mutated and RAD3-related
Pp3c6_16050	AT3G12380	ARP5.ATARP5	actin-related protein 5
Pp3c7_7920	AT5G20850	ATRAD51.RAD51	RAS associated with diabetes protein 51
Pp3c8_12030	AT5G02820	BIN5.RHL2	Spo11/DNA topoisomerase VI. subunit A protein
Pp3c8_16660	AT5G58760	DDB2	damaged DNA binding 2
Pp3c8_17910	AT3G19210	ATRAD54.CHR25.RAD54	homolog of RAD54
Pp3c8_18800	AT3G10690	GYRA	DNA GYRASE A
Pp3c10_8490	AT1G79650	RAD23.RAD23B	Rad23 UV excision repair protein family
Pp3c10_16570	AT1G03190	ATXPD.UVH6	RAD3-like DNA-binding helicase protein
Pp3c11_22000	AT5G20850	ATRAD51.RAD51	RAS associated with diabetes protein 51
Pp3c12_1610	AT1G31360	ATRECQ2.MED34.RECQL2	RECQ helicase L2
Pp3c12_2410	AT5G25480	AtDNMT2.DNMT2	DNA methyltransferase-2
Pp3c12_3560	AT4G09680	ATCTC1.CTC1	conserved telomere maintenance component 1
Pp3c12_4780	AT5G57970	NaN	DNA glycosylase superfamily protein
Pp3c12_23740	AT4G22910	CCS52A1.FZR2	FIZZY-related 2
Pp3c13_19570	AT1G77620	NaN	P-loop containing nucleoside triphosphate hydrolases superfamily protein
Pp3c13_20209	AT1G08130	ATLIG1.LIG1	DNA ligase 1
Pp3c14_7440	AT1G05180	AXR1	NAD(P)-binding Rossmann-fold superfamily protein
Pp3c15_140	AT5G18070	DRT101	phosphoglucosamine mutase-related
Pp3c15_7530	AT1G21710	ATOGG1.OGG1	8-oxoguanine-DNA glycosylase 1
Pp3c15_23590	AT2G18760	CHR8	chromatin remodeling 8
Pp3c16_6690	AT3G19210	ATRAD54.CHR25.RAD54	homolog of RAD54
Pp3c16_21800	AT1G63160	RFC2	replication factor C 2
Pp3c17_13600	AT4G29910	ATORC5.ORC5	origin recognition complex protein 5
Pp3c17_13784	AT1G65070	NaN	DNA mismatch repair protein MutS. type 2
Pp3c17_14880	AT2G19490	NaN	recA DNA recombination family protein
Pp3c17_16180	AT1G67320	NaN	DNA primase. large subunit family
Pp3c18_14120	AT4G02070	ATMSH6.MSH6.MSH6-1	MUTS homolog 6
Pp3c18_14740	AT2G37560	ATORC2.ORC2	origin recognition complex second largest subunit 2
Pp3c19_5540	AT4G10180	ATDET1.DET1.FUS2	light-mediated development protein 1 / deetiolated1 (DET1)
Pp3c19_21980	AT2G01440	NaN	DEAD/DEAH box RNA helicase family protein
Pp3c20_10450	AT5G45400	ATRPA70C.RPA70C	Replication factor-A protein 1-related
Pp3c20_17250	AT1G80190	PSF1	partner of SLD five 1
Pp3c21_7810	AT5G24850	CRY3	cryptochrome 3
Pp3c21_8640	AT2G40550	ETG1	E2F target gene 1
Pp3c21_8660	AT1G52500	ATFPG-1.ATFPG-2.ATMMH-1.ATMMH-2.FPG-1.FPG-2.MMH-1.MMH-2	MUTM homolog-1
Pp3c22_11100	AT1G48050	ATKU80.KU80	Ku80 family protein
Pp3c23_390	AT5G44635	MCM6	minichromosome maintenance (MCM2/3/5) family protein
Pp3c23_8000	AT1G29630	NaN	5'-3' exonuclease family protein
Pp3c24_2350	AT5G54260	ATMRE11.MRE11	DNA repair and meiosis protein (Mre11)
Pp3c25_4540	AT2G19480	NAP1;2.NFA02.NFA2	nucleosome assembly protein 1;2
Pp3c24_16390	AT3G20540	POLGAMMA1	polymerase gamma 1
Pp3c24_17350	AT3G52905	NaN	Polynucleotidyl transferase. ribonuclease H-like superfamily protein
Pp3c26_6700	AT4G18820	NaN	AAA-type ATPase family protein
Pp3c26_11300	AT1G30010	NaN	Intron maturase. type II family protein
Pp3c26_14310	AT2G24490	ATRPA2.ATRPA32A.ROR1.RPA2.RPA32A	replicon protein A2

*Four of 63 genes are colored in red as the common DEGs both 1cell-DGE and 5'DGE.

Supplementary Table S9. DEGs in 24 h-high identified using 1cell-DGE categorized by the GO term "protein metabolic process".

Pp3.3 gene*	Best hit At gene*	At_symbol*	At_description*
Pp3c1_420	AT5G56670	NaN	Ribosomal protein S30 family protein
Pp3c1_900	AT3G20050	ATTCP-1.TCP-1	T-complex protein 1 alpha subunit
Pp3c1_1530	AT3G10950	NaN	Zinc-binding ribosomal protein family protein
Pp3c1_1800	AT1G26880	NaN	Ribosomal protein L34e superfamily protein
Pp3c1_2390	AT1G07510	ftsh10	FTSH protease 10
Pp3c1_3170	AT1G26910	RPL10B	Ribosomal protein L16p/L10e family protein
Pp3c1_3480	AT3G05590	RPL18	ribosomal protein L18
Pp3c1_4030	AT1G41880	NaN	Ribosomal protein L35Ae family protein
Pp3c1_4290	AT5G59850	NaN	Ribosomal protein S8 family protein
Pp3c1_5320	AT3G44890	RPL9	ribosomal protein L9
Pp3c1_5330	AT3G05590	RPL18	ribosomal protein L18
Pp3c1_6160	AT4G00100	ATRPS13A.PFL2.RPS13.RPS13A	ribosomal protein S13A
Pp3c1_6650	AT1G67430	NaN	Ribosomal protein L22p/L17e family protein
Pp3c1_6950	AT5G43100	NaN	Eukaryotic aspartyl protease family protein
Pp3c1_7160	AT5G39740	OLI7.RPL5B	ribosomal protein L5 B
Pp3c1_7830	AT5G56940	NaN	Ribosomal protein S16 family protein
Pp3c1_7900	AT5G53400	BOB1	HSP20-like chaperones superfamily protein
Pp3c1_8280	AT2G40010	NaN	Ribosomal protein L10 family protein
Pp3c1_8290	AT5G58420	NaN	Ribosomal protein S4 (RPS4A) family protein
Pp3c1_10010	AT5G64050	ATERS.ERS.OVA3	glutamate tRNA synthetase
Pp3c1_10400	AT5G36210	NaN	alpha/beta-Hydrolases superfamily protein
Pp3c1_15830	AT5G48760	NaN	Ribosomal protein L13 family protein
Pp3c1_16170	AT2G36620	RPL24A	ribosomal protein L24
Pp3c1_16810	AT2G31010	NaN	Protein kinase superfamily protein
Pp3c1_18050	AT2G44860	NaN	Ribosomal protein L24e family protein
Pp3c1_18280	AT5G56890	NaN	Protein kinase superfamily protein
Pp3c1_18390	AT3G22750	NaN	Protein kinase superfamily protein
Pp3c1_18400	AT4G25740	NaN	RNA binding Plectin/S10 domain-containing protein
Pp3c1_19680	AT1G25350	OVA9	glutamine-tRNA ligase. putative/ glutaminyl-tRNA synthetase. putative/ GlnRS. putative
Pp3c1_20880	AT3G60210	NaN	GroES-like family protein
Pp3c1_21660	AT5G09900	EMB2107.MSA.RPN5A	26S proteasome regulatory subunit. putative (RPN5)
Pp3c1_21860	AT3G45030	NaN	Ribosomal protein S10p/S20e family protein
Pp3c1_21950	AT5G19990	ATSUG1.RPT6A	regulatory particle triple-A ATPase 6A
Pp3c1_22100	AT3G13690	NaN	Protein kinase protein with adenine nucleotide alpha hydrolases-like domain
Pp3c1_22820	AT5G27820	NaN	Ribosomal L18p/L5e family protein
Pp3c1_23220	AT5G09590	HSC70-5.MTHSC70-2	mitochondrial HSO70 2
Pp3c1_24120	AT1G72370	AP40.P40.RP40.RPSAA	40s ribosomal protein SA
Pp3c1_24300	AT1G74050	NaN	Ribosomal protein L6 family protein
Pp3c1_26280	AT3G49010	ATBBC1.BBC1.RSU2	breast basic conserved 1
Pp3c1_27970	AT4G06599	NaN	ubiquitin family protein
Pp3c1_28770	AT3G10090	NaN	Nucleic acid-binding. OB-fold-like protein
Pp3c1_29390	AT4G26310	NaN	elongation factor P (EF-P) family protein
Pp3c1_29840	AT5G22130	PNT1	mannosyltransferase family protein
Pp3c1_31160	AT2G26890	GRV2.KAM2	DNAJ heat shock N-terminal domain-containing protein
Pp3c1_32090	AT5G58270	ATATM3.ATM3.STA1	ABC transporter of the mitochondrion 3
Pp3c1_32320	AT5G23290	PFD5	prefoldin 5
Pp3c1_32410	AT1G26740	NaN	Ribosomal L32p protein family
Pp3c1_32490	AT4G18100	NaN	Ribosomal protein L32e
Pp3c1_32520	AT1G17110	UBP15	ubiquitin-specific protease 15
Pp3c1_34390	AT2G19740	NaN	Ribosomal protein L31e family protein
Pp3c1_36090	AT1G20140	ASK4.SK4	SKP1-like 4
Pp3c1_36220	AT5G24510	NaN	60S acidic ribosomal protein family
Pp3c1_37420	AT4G16720	NaN	Ribosomal protein L23/L15e family protein
Pp3c1_38560	AT5G58290	RPT3	regulatory particle triple-A ATPase 3
Pp3c1_39350	AT4G32830	AtAUR1.AUR1	ataurora1
Pp3c1_39640	AT2G19170	SLP3	subtilisin-like serine protease 3
Pp3c1_39880	AT5G62620	NaN	Galactosyltransferase family protein
Pp3c1_40490	AT5G46160	NaN	Ribosomal protein L14p/L23e family protein
Pp3c1_41710	AT5G57500	NaN	Galactosyltransferase family protein
Pp3c2_660	AT4G04860	DER2.2	DERLIN-2.2
Pp3c2_1120	AT1G64300	NaN	Protein kinase family protein
Pp3c2_2220	AT1G77280	NaN	Protein kinase protein with adenine nucleotide alpha hydrolases-like domain
Pp3c2_3850	AT3G62120	NaN	Class II aaRS and biotin synthetases superfamily protein
Pp3c2_5060	AT5G35590	PAA1	proteasome alpha subunit A1
Pp3c2_5260	AT2G19170	SLP3	subtilisin-like serine protease 3
Pp3c2_5780	AT4G16720	NaN	Ribosomal protein L23/L15e family protein
Pp3c2_6730	AT3G44010	NaN	Ribosomal protein S14p/S29e family protein
Pp3c2_9490	AT5G05920	DHS.EDA22	deoxyhypusine synthase
Pp3c2_9590	AT4G18100	NaN	Ribosomal protein L32e
Pp3c2_9650	AT3G53740	NaN	Ribosomal protein L36e family protein
Pp3c2_9790	AT5G03160	ATP58IPK.P58IPK	homolog of mamallian P58IPK
Pp3c2_9830	AT1G14610	TWN2.VALRS	valyl-tRNA synthetase / valine--tRNA ligase (VALRS)
Pp3c2_10060	AT3G02080	NaN	Ribosomal protein S19e family protein
Pp3c2_11270	AT1G05180	AXR1	NAD(P)-binding Rossmann-fold superfamily protein
Pp3c2_11670	AT5G23535	NaN	KOW domain-containing protein
Pp3c2_11870	AT2G25880	AtAUR2.AUR2	ataurora2
Pp3c2_12240	AT5G09590	HSC70-5.MTHSC70-2	mitochondrial HSO70 2
Pp3c2_13300	AT1G74060	NaN	Ribosomal protein L6 family protein
Pp3c2_13690	AT3G48930	EMB1080	Nucleic acid-binding. OB-fold-like protein
Pp3c2_13870	AT5G05080	ATUBC22.UBC22	ubiquitin-conjugating enzyme 22
Pp3c2_14800	AT4G39200	NaN	Ribosomal protein S25 family protein
Pp3c2_16490	AT2G19740	NaN	Ribosomal protein L31e family protein
Pp3c2_16540	AT2G22740	SDG23.SUVH6	SU(VAR)3-9 homolog 6
Pp3c2_16770	AT4G39280	NaN	phenylalanyl-tRNA synthetase. putative / phenylalanine--tRNA ligase. putative
Pp3c2_18070	AT3G60740	CHO.EMB133.TFC D.TTN1	ARM repeat superfamily protein
Pp3c2_18620	AT4G25740	NaN	RNA binding Plectin/S10 domain-containing protein
Pp3c2_19170	AT3G60210	NaN	GroES-like family protein
Pp3c2_24130	AT5G02960	NaN	Ribosomal protein S12/S23 family protein

Pp3.3 gene*	Best hit At gene*	At_symbol*	At_description*
Pp3c2_24640	AT3G51800	ATEBP1.ATG2.EBP1	metallopeptidase M24 family protein
Pp3c2_24930	AT2G17360	NaN	Ribosomal protein S4 (RPS4A) family protein
Pp3c2_27110	AT1G15390	ATDEF1.PDF1A	peptide deformylase 1A
Pp3c2_28870	AT5G56670	NaN	Ribosomal protein S30 family protein
Pp3c2_29600	AT1G26910	RPL10B	Ribosomal protein L16p/L10e family protein
Pp3c2_29840	AT1G67430	NaN	Ribosomal protein L22p/L17e family protein
Pp3c2_30830	AT2G22740	SDG23.SUVH6	SU(VAR)3-9 homolog 6
Pp3c2_31540	AT5G39740	OLI7.RPL5B	ribosomal protein L5 B
Pp3c2_32050	AT5G59850	NaN	Ribosomal protein S8 family protein
Pp3c2_32310	AT4G18040	AT.EIF4E1.CUM1.EIF4E.eIF4E1	eukaryotic translation initiation factor 4E
Pp3c2_32510	AT3G05590	RPL18	ribosomal protein L18
Pp3c2_33280	AT4G18730	RPL16B	ribosomal protein L16B
Pp3c2_35700	AT4G31460	NaN	Ribosomal L28 family
Pp3c2_38140	AT5G56940	NaN	Ribosomal protein S16 family protein
Pp3c3_270	AT5G57020	ATNMT1.NMT1	myristoyl-CoA:protein N-myristoyltransferase
Pp3c3_1140	AT1G07770	RPS15A	ribosomal protein S15A
Pp3c3_1240	AT1G29150	ATS9.RPN6	non-ATPase subunit 9
Pp3c3_1620	AT3G10950	NaN	Zinc-binding ribosomal protein family protein
Pp3c3_2100	AT4G15420	NaN	Ubiquitin fusion degradation UFD1 family protein
Pp3c3_2800	AT5G02610	NaN	Ribosomal L29 family protein
Pp3c3_3090	AT3G22300	RPS10	ribosomal protein S10
Pp3c3_3400	AT1G27600	I9H.IRX9-L	Nucleotide-diphospho-sugar transferases superfamily protein
Pp3c3_3480	AT1G74270	NaN	Ribosomal protein L35Ae family protein
Pp3c3_5550	AT1G57660	NaN	Translation protein SH3-like family protein
Pp3c3_5570	AT3G29160	AKIN11.ATKIN11.KIN11.SNRK1.2	SNF1 kinase homolog 11
Pp3c3_5690	AT4G30930	NFD1	Ribosomal protein L21
Pp3c3_8500	AT3G56150	ATEIF3C-1.ATTIF3C1.EIF3C.EIF3C-1.TIF3C1	eukaryotic translation initiation factor 3C
Pp3c3_8580	AT2G39940	COI1	RNI-like superfamily protein
Pp3c3_8930	AT3G53020	RPL24.RPL24B.STV1	Ribosomal protein L24e family protein
Pp3c3_10260	AT4G14320	NaN	Zinc-binding ribosomal protein family protein
Pp3c3_10840	AT2G36620	RPL24A	ribosomal protein L24
Pp3c3_11250	AT2G34480	NaN	Ribosomal protein L18ae/LX family protein
Pp3c3_11990	AT1G51980	NaN	Insulinase (Peptidase family M16) protein
Pp3c3_12210	AT2G18020	EMB2296	Ribosomal protein L2 family
Pp3c3_12560	AT5G63930	NaN	Leucine-rich repeat protein kinase family protein
Pp3c3_14880	AT5G04800	NaN	Ribosomal S17 family protein
Pp3c3_15970	AT5G61790	ATCNX1.CNX1	calnexin 1
Pp3c3_15980	AT5G61790	ATCNX1.CNX1	calnexin 1
Pp3c3_16200	AT2G26890	GRV2.KAM2	DNAJ heat shock N-terminal domain-containing protein
Pp3c3_18921	AT3G17750	NaN	Protein kinase superfamily protein
Pp3c3_19700	AT5G28060	NaN	Ribosomal protein S24e family protein
Pp3c3_21130	AT3G49010	ATBBC1.BBC1.RSU2	breast basic conserved 1
Pp3c3_23160	AT1G06200	NaN	Peptidase S24/S26A/S26B/S26C family protein
Pp3c3_23540	AT3G58700	NaN	Ribosomal L5P family protein
Pp3c3_23720	AT5G15200	NaN	Ribosomal protein S4
Pp3c3_25300	AT2G18110	NaN	Translation elongation factor EF1B/ribosomal protein S6 family protein
Pp3c3_25480	AT3G52590	EMB2167.ERD16.HAP4.UBQ1	ubiquitin extension protein 1
Pp3c3_25640	AT2G43460	NaN	Ribosomal L38e protein family
Pp3c3_26590	AT5G02500	AT-HSC70-1.HSC70.HSC70-1.HSP70-1	heat shock cognate protein 70-1
Pp3c3_26860	AT4G36420	NaN	Ribosomal protein L12 family protein
Pp3c3_28940	AT5G36950	DegP10	DegP protease 10
Pp3c3_32490	AT1G49780	PUB26	plant U-box 26
Pp3c3_35340	AT3G61790	NaN	Protein with RING/U-box and TRAF-like domains
Pp3c3_35430	AT5G08335	ATICMTB.ATSTE14B.ICMTB	Isoprenylcysteine carboxyl methyltransferase (ICMT) family
Pp3c3_35760	AT5G47320	RPS19	ribosomal protein S19
Pp3c3_36280	AT1G53880	NaN	Eukaryotic translation initiation factor 2B (eIF-2B) family protein
Pp3c3_37810	AT2G39630	NaN	Nucleotide-diphospho-sugar transferases superfamily protein
Pp3c3_37890	AT5G12180	CPK17	calcium-dependent protein kinase 17
Pp3c4_1120	AT5G06460	ATUBA2.UBA 2	ubiquitin activating enzyme 2
Pp3c4_1480	AT3G24550	ATPERK1.PERK1	proline extensin-like receptor kinase 1
Pp3c4_3100	AT1G23100	NaN	GroES-like family protein
Pp3c4_3220	AT5G27700	NaN	Ribosomal protein S21e
Pp3c4_3300	AT4G01320	ATSTE24.STE24	Peptidase family M48 family protein
Pp3c4_6350	AT3G49080	NaN	Ribosomal protein S5 domain 2-like superfamily protein
Pp3c4_6500	AT3G52450	PUB22	plant U-box 22
Pp3c4_6722	AT2G17200	DSK2	ubiquitin family protein
Pp3c4_7510	AT3G11710	ATKRS-1	lysyl-tRNA synthetase 1
Pp3c4_8620	AT3G55280	RPL23AB	ribosomal protein L23AB
Pp3c4_11370	AT5G59240	NaN	Ribosomal protein S8e family protein
Pp3c4_11930	AT2G36170	NaN	Ubiquitin supergroup;Ribosomal protein L40e
Pp3c4_12690	AT4G36130	NaN	Ribosomal protein L2 family
Pp3c4_13370	AT3G58700	NaN	Ribosomal L5P family protein
Pp3c4_13380	AT3G11710	ATKRS-1	lysyl-tRNA synthetase 1
Pp3c4_13680	AT1G51980	NaN	Insulinase (Peptidase family M16) protein
Pp3c4_14160	AT4G34670	NaN	Ribosomal protein S3Ae
Pp3c4_15290	AT5G15200	NaN	Ribosomal protein S4
Pp3c4_15480	AT1G77940	NaN	Ribosomal protein L7Ae/L30e/S12e/Gadd45 family protein
Pp3c4_17860	AT2G44900	ARABIDILLO-1	ARABIDILLO-1
Pp3c4_18360	AT2G37050	NaN	Leucine-rich repeat protein kinase family protein
Pp3c4_19000	AT2G47110	UBQ6	ubiquitin 6
Pp3c4_19980	AT5G26360	NaN	TCP-1/cpn60 chaperonin family protein
Pp3c4_21130	AT3G62600	ATERDJ3B.ERDJ3B	DNAJ heat shock family protein
Pp3c4_21520	AT1G57860	NaN	Translation protein SH3-like family protein
Pp3c4_24414	AT3G56150	ATEIF3C-1.ATTIF3C1.EIF3C.EIF3C-1.TIF3C1	eukaryotic translation initiation factor 3C
Pp3c4_25010	AT3G50530	CRK	CDPK-related kinase
Pp3c4_26450	AT4G11420	ATEIF3A-1.ATTIF3A1.EIF3A.EIF3A-1.TIF3A1	eukaryotic translation initiation factor 3A
Pp3c4_27540	AT5G59850	NaN	Ribosomal protein S8 family protein
Pp3c4_28230	AT4G02010	NaN	Protein kinase superfamily protein
Pp3c4_29560	AT3G13882	NaN	Ribosomal protein L34

Pp3.3 gene*	Best hit At gene*	At_symbol*	At_description*
Pp3c4_29970	AT1G74270	NaN	Ribosomal protein L35Ae family protein
Pp3c5_1590	AT2G27020	PAG1	20S proteasome alpha subunit G1
Pp3c5_2800	AT1G34030	NaN	Ribosomal protein S13/S18 family
Pp3c5_3870	AT3G58700	NaN	Ribosomal L5P family protein
Pp3c5_4330	AT1G77940	NaN	Ribosomal protein L7Ae/L30e/S12e/Gadd45 family protein
Pp3c5_5330	AT5G35530	NaN	Ribosomal protein S3 family protein
Pp3c5_5750	AT1G07770	RPS15A	ribosomal protein S15A
Pp3c5_6000	AT5G03850	NaN	Nucleic acid-binding. OB-fold-like protein
Pp3c5_6600	AT2G13540	ABH1.ATCBP80.CBP80.ENS	ARM repeat superfamily protein
Pp3c5_6960	AT1G79620	NaN	Leucine-rich repeat protein kinase family protein
Pp3c5_8950	AT2G41790	NaN	Insulinase (Peptidase family M16) family protein
Pp3c5_9890	AT2G33840	NaN	Tyrosyl-tRNA synthetase. class Ib. bacterial/mitochondrial
Pp3c5_10680	AT3G20230	NaN	Ribosomal L18p/L5e family protein
Pp3c5_10720	AT1G32930	NaN	Galactosyltransferase family protein
Pp3c5_12940	AT4G35490	MRPL11	mitochondrial ribosomal protein L11
Pp3c5_13770	AT3G12580	ATHSP70.HSP70	heat shock protein 70
Pp3c5_15390	AT1G02305	NaN	Cysteine proteinases superfamily protein
Pp3c5_15520	AT4G15900	PRL1	pleiotropic regulatory locus 1
Pp3c5_15740	AT3G58140	NaN	phenylalanyl-tRNA synthetase class IIc family protein
Pp3c5_16070	AT2G40510	NaN	Ribosomal protein S26e family protein
Pp3c5_16830	AT5G20890	NaN	TCP-1/cpn60 chaperonin family protein
Pp3c5_19540	AT1G49340	ATPI4K ALPHA	Phosphatidylinositol 3- and 4-kinase family protein
Pp3c5_19870	AT1G60220	OTS1.ULP1D	UB-like protease 1D
Pp3c5_20500	AT5G20920	EIF2 BETA.EMB1401	eukaryotic translation initiation factor 2 beta subunit
Pp3c5_21920	AT3G05530	ATS6A.2.RPT5A	regulatory particle triple-A ATPase 5A
Pp3c5_25290	AT2G39550	ATGGT-IB.GGB.PGGT-I	Prenyltransferase family protein
Pp3c5_26860	AT1G70600	NaN	Ribosomal protein L18e/L15 superfamily protein
Pp3c5_27000	AT1G61580	ARP2.RPL3B	R-protein L3 B
Pp3c5_27030	AT1G61580	ARP2.RPL3B	R-protein L3 B
Pp3c5_28210	AT3G13920	EIF4A1.RH4.TIF4A1	eukaryotic translation initiation factor 4A1
Pp3c6_610	AT4G31985	NaN	Ribosomal protein L39 family protein
Pp3c6_1080	AT3G13920	EIF4A1.RH4.TIF4A1	eukaryotic translation initiation factor 4A1
Pp3c6_1920	AT4G11160	NaN	Translation initiation factor 2. small GTP-binding protein
Pp3c6_3470	AT1G70600	NaN	Ribosomal protein L18e/L15 superfamily protein
Pp3c6_6410	AT5G27660	NaN	Trypsin family protein with PDZ domain
Pp3c6_7730	AT5G07090	NaN	Ribosomal protein S4 (RPS4A) family protein
Pp3c6_8910	AT2G40590	NaN	Ribosomal protein S26e family protein
Pp3c6_14960	AT1G12460	NaN	Leucine-rich repeat protein kinase family protein
Pp3c6_15940	AT5G20890	NaN	TCP-1/cpn60 chaperonin family protein
Pp3c6_17230	AT1G10840	TIF3H1	translation initiation factor 3 subunit H1
Pp3c6_17680	AT5G15200	NaN	Ribosomal protein S4
Pp3c6_17840	AT2G47390	NaN	Prolyl oligopeptidase family protein
Pp3c6_19330	AT3G10950	NaN	Zinc-binding ribosomal protein family protein
Pp3c6_19470	AT2G39630	NaN	Nucleotide-diphospho-sugar transferases superfamily protein
Pp3c6_19730	AT2G16250	NaN	Leucine-rich repeat protein kinase family protein
Pp3c6_19930	AT2G33840	NaN	Tyrosyl-tRNA synthetase. class Ib. bacterial/mitochondrial
Pp3c6_19960	AT1G77550	NaN	tubulin-tyrosine ligases;tubulin-tyrosine ligases
Pp3c6_20680	AT4G38630	ATMCB1.MBP1.MCB1.RPN10	regulatory particle non-ATPase 10
Pp3c6_21770	AT1G34030	NaN	Ribosomal protein S13/S18 family
Pp3c6_21830	AT1G34030	NaN	Ribosomal protein S13/S18 family
Pp3c6_23490	AT3G53090	UPL7	ubiquitin-protein ligase 7
Pp3c6_26460	AT3G48330	ATPIMT1.PIMT1	protein-l-isoaspartate methyltransferase 1
Pp3c7_370	AT5G25780	ATEIF3B-2.EIF3B.EIF3B-2	eukaryotic translation initiation factor 3B-2
Pp3c7_720	AT1G17720	ATB BETA	Protein phosphatase 2A. regulatory subunit PR55
Pp3c7_1440	AT5G55830	NaN	Concanavalin A-like lectin protein kinase family protein
Pp3c7_2620	AT5G35530	NaN	Ribosomal protein S3 family protein
Pp3c7_3770	AT3G13920	EIF4A1.RH4.TIF4A1	eukaryotic translation initiation factor 4A1
Pp3c7_3920	AT4G31670	UBP18	ubiquitin-specific protease 18
Pp3c7_4430	AT4G18950	NaN	Integrin-linked protein kinase family
Pp3c7_5560	AT2G39990	AteIF3f.EIF2.eIF3F	eukaryotic translation initiation factor 2
Pp3c7_5660	AT3G61140	ATFUS6.ATSK31.COP11.CSN1.EMB78.FUS6.SK31	26S proteasome. regulatory subunit Rpn7;Proteasome component (PCI) domain
Pp3c7_5780	AT5G10360	EMB3010.RPS6B	Ribosomal protein S6e
Pp3c7_7460	AT1G51710	ATUBP6.UBP6	ubiquitin-specific protease 6
Pp3c7_8500	AT3G56070	ROC2	rotamase cyclophilin 2
Pp3c7_8710	AT5G04240	ELF6	Zinc finger (C2H2 type) family protein / transcription factor jumonji (jmj) family protein
Pp3c7_9780	AT4G07990	NaN	Chaperone DnaJ-domain superfamily protein
Pp3c7_12270	AT5G48760	NaN	Ribosomal protein L13 family protein
Pp3c7_14130	AT2G31010	NaN	Protein kinase superfamily protein
Pp3c7_14200	AT5G02960	NaN	Ribosomal protein S12/S23 family protein
Pp3c7_15200	AT5G49910	CPHSC70-2.HEAT SHOCK PROTEIN 70-2.HSC70-7	chloroplast heat shock protein 70-2
Pp3c7_15950	AT4G23160	CRK8	cysteine-rich RLK (RECEPTOR-like protein kinase) 8
Pp3c7_20040	AT3G57340	NaN	Heat shock protein DnaJ. N-terminal with domain of unknown function (DUF1977)
Pp3c7_20120	AT4G14320	NaN	Zinc-binding ribosomal protein family protein
Pp3c7_20160	AT2G42740	RPL16A	ribosomal protein large subunit 16A
Pp3c7_21070	AT5G04800	NaN	Ribosomal S17 family protein
Pp3c7_21140	AT5G04800	NaN	Ribosomal S17 family protein
Pp3c7_21380	AT1G26880	NaN	Ribosomal protein L34e superfamily protein
Pp3c7_21930	AT1G69620	RPL34	ribosomal protein L34
Pp3c7_22030	AT3G05590	RPL18	ribosomal protein L18
Pp3c7_22170	AT5G02500	AT-HSC70-1.HSC70.HSC70-1.HSP70-1	heat shock cognate protein 70-1
Pp3c7_22400	AT1G26910	RPL10B	Ribosomal protein L16p/L10e family protein
Pp3c7_22710	AT5G12480	CPK7	calmodulin-domain protein kinase 7
Pp3c7_23150	AT4G30610	BRS1.SCPL24	alpha/beta-Hydrolases superfamily protein
Pp3c7_23800	AT5G07090	NaN	Ribosomal protein S4 (RPS4A) family protein
Pp3c7_24050	AT3G46100	ATHRS1.HRS1	Histidyl-tRNA synthetase 1
Pp3c7_24370	AT3G17000	UBC32	ubiquitin-conjugating enzyme 32
Pp3c7_25220	AT1G67430	NaN	Ribosomal protein L22p/L17e family protein
Pp3c7_25240	AT1G67430	NaN	Ribosomal protein L22p/L17e family protein
Pp3c7_25660	AT5G39740	OLI7.RPL5B	ribosomal protein L5 B

Pp3.3 gene*	Best hit At gene*	At_symbol*	At_description*
Pp3c7_26650	AT5G56670	NaN	Ribosomal protein S30 family protein
Pp3c7_26720	AT4G00100	ATRPS13A.PFL2.RPS13.RPS13A	ribosomal protein S13A
Pp3c7_26760	AT5G02500	AT-HSC70-1.HSC70.HSC70-1.HSP70-1	heat shock cognate protein 70-1
Pp3c8_1610	AT3G58660	NaN	Ribosomal protein L1p/L10e family
Pp3c8_2540	AT2G24230	NaN	Leucine-rich repeat protein kinase family protein
Pp3c8_5640	AT3G57490	NaN	Ribosomal protein S5 family protein
Pp3c8_5770	AT1G70600	NaN	Ribosomal protein L18e/L15 superfamily protein
Pp3c8_6120	AT5G54590	CRLK1	Protein kinase superfamily protein
Pp3c8_7210	AT1G23100	NaN	GroES-like family protein
Pp3c8_9480	AT5G24260	NaN	prolyl oligopeptidase family protein
Pp3c8_12080	AT1G04480	NaN	Ribosomal protein L14p/L23e family protein
Pp3c8_13880	AT2G34480	NaN	Ribosomal protein L18ae/LX family protein
Pp3c8_14230	AT2G47110	UBQ6	ubiquitin 6
Pp3c8_16130	AT3G04400	emb2171	Ribosomal protein L14p/L23e family protein
Pp3c8_16210	AT4G28360	NaN	Ribosomal protein L22p/L17e family protein
Pp3c8_16820	AT2G37270	ATRPS5B.RPS5B	ribosomal protein 5B
Pp3c8_16860	AT2G37270	ATRPS5B.RPS5B	ribosomal protein 5B
Pp3c8_17390	AT2G29960	ATCYP5.CYP19-4.CYP5	cyclophilin 5
Pp3c8_18130	AT5G49510	PFD3	prefoldin 3
Pp3c8_19600	AT4G22720	NaN	Actin-like ATPase superfamily protein
Pp3c8_22360	AT4G16720	NaN	Ribosomal protein L23/L15e family protein
Pp3c8_22380	AT5G24510	NaN	60S acidic ribosomal protein family
Pp3c8_22480	AT1G48830	NaN	Ribosomal protein S7e family protein
Pp3c8_24770	AT5G35590	PAA1	proteasome alpha subunit A1
Pp3c8_24950	AT2G39960	NaN	Microsomal signal peptidase 25 kDa subunit (SPC25)
Pp3c8_25050	AT2G20450	NaN	Ribosomal protein L14
Pp3c9_380	AT3G62870	NaN	Ribosomal protein L7Ae/L30e/S12e/Gadd45 family protein
Pp3c9_950	AT3G26618	ERF1-3	eukaryotic release factor 1-3
Pp3c9_1560	AT1G04870	ATPRMT10.PRMT10	protein arginine methyltransferase 10
Pp3c9_1670	AT1G07210	NaN	Ribosomal protein S18
Pp3c9_3330	AT4G29330	DER1	DERLIN-1
Pp3c9_5860	AT2G17890	CPK16	calcium-dependent protein kinase 16
Pp3c9_6640	AT5G56000	AtHsp90.4.Hsp81.4	HEAT SHOCK PROTEIN 81.4
Pp3c9_6690	AT5G56000	AtHsp90.4.Hsp81.4	HEAT SHOCK PROTEIN 81.4
Pp3c9_7700	AT2G31170	SYCO ARATH	Cysteinyl-tRNA synthetase. class Ia family protein
Pp3c9_8500	AT1G52300	NaN	Zinc-binding ribosomal protein family protein
Pp3c9_11840	AT3G08760	ATSIK	Protein kinase superfamily protein
Pp3c9_14000	AT5G53070	NaN	Ribosomal protein L9/RNase H1
Pp3c9_14720	AT3G16780	NaN	Ribosomal protein L19e family protein
Pp3c9_18040	AT5G48580	FKBP15-2	FK506- and rapamycin-binding protein 15 kD-2
Pp3c9_18560	AT1G02090	ATCSN7.COP15.CSN7.FUS5	Proteasome component (PCI) domain protein
Pp3c9_18780	AT4G31985	NaN	Ribosomal protein L39 family protein
Pp3c9_21480	AT3G11510	NaN	Ribosomal protein S11 family protein
Pp3c9_23630	AT1G16890	UBC13B.UBC36	ubiquitin-conjugating enzyme 36
Pp3c9_23730	AT1G08750	NaN	Peptidase C13 family
Pp3c9_24220	AT3G16560	NaN	Protein phosphatase 2C family protein
Pp3c9_24880	AT4G19006	NaN	Proteasome component (PCI) domain protein
Pp3c9_25280	AT5G57610	NaN	Protein kinase superfamily protein with octicosapeptide/Phox/Bem1p domain
Pp3c9_26010	AT3G08980	NaN	Peptidase S24/S26A/S26B/S26C family protein
Pp3c10_990	AT4G25740	NaN	RNA binding Plectin/S10 domain-containing protein
Pp3c10_2110	AT4G13670	PTAC5	plastid transcriptionally active 5
Pp3c10_2600	AT5G19990	ATSUG1.RPT6A	regulatory particle triple-A ATPase 6A
Pp3c10_4970	AT4G34110	ATPAB2.PAB2.PABP2	poly(A) binding protein 2
Pp3c10_7370	AT3G49010	ATBBC1.BBC1.RSU2	breast basic conserved 1
Pp3c10_7980	AT3G43980	NaN	Ribosomal protein S14p/S29e family protein
Pp3c10_8090	AT2G19740	NaN	Ribosomal protein L31e family protein
Pp3c10_8490	AT1G79650	RAD23.RAD23B	Rad23 UV excision repair protein family
Pp3c10_8510	AT2G47760	ALG3.AtALG3	asparagine-linked glycosylation 3
Pp3c10_9620	AT4G11120	NaN	translation elongation factor Ts (EF-Ts). putative
Pp3c10_9960	AT1G30000	MNS3	alpha-mannosidase 3
Pp3c10_12320	AT5G11900	NaN	Translation initiation factor SUI1 family protein
Pp3c10_13240	AT5G26360	NaN	TCP-1/cpn60 chaperonin family protein
Pp3c10_14440	AT4G40042	NaN	Microsomal signal peptidase 12 kDa subunit (SPC12)
Pp3c10_14850	AT5G06460	ATUBA2.UBA 2	ubiquitin activating enzyme 2
Pp3c10_15140	AT2G18110	NaN	Translation elongation factor EF1B/ribosomal protein S6 family protein
Pp3c10_15500	AT1G70460	RHS10	root hair specific 10
Pp3c10_20240	AT3G59540	NaN	Ribosomal L38e protein family
Pp3c10_21170	AT4G25740	NaN	RNA binding Plectin/S10 domain-containing protein
Pp3c10_25270	AT3G52590	EMB2167.ERD16.HAP4.UBQ1	ubiquitin extension protein 1
Pp3c10_25460	AT1G61580	ARP2.RPL3B	R-protein L3 B
Pp3c11_1000	AT4G26300	emb1027	Arginyl-tRNA synthetase. class Ic
Pp3c11_1010	AT5G02500	AT-HSC70-1.HSC70.HSC70-1.HSP70-1	heat shock cognate protein 70-1
Pp3c11_1100	AT4G00100	ATRPS13A.PFL2.RPS13.RPS13A	ribosomal protein S13A
Pp3c11_2110	AT3G20050	ATTCP-1.TCP-1	T-complex protein 1 alpha subunit
Pp3c11_2940	AT1G26880	NaN	Ribosomal protein L34e superfamily protein
Pp3c11_3090	AT1G26880	NaN	Ribosomal protein L34e superfamily protein
Pp3c11_3690	AT2G17360	NaN	Ribosomal protein S4 (RPS4A) family protein
Pp3c11_3700	AT2G40010	NaN	Ribosomal protein L10 family protein
Pp3c11_4130	AT1G26910	RPL10B	Ribosomal protein L16p/L10e family protein
Pp3c11_5840	AT1G67430	NaN	Ribosomal protein L22p/L17e family protein
Pp3c11_6420	AT5G39740	OLI7.RPL5B	ribosomal protein L5 B
Pp3c11_6620	AT5G02500	AT-HSC70-1.HSC70.HSC70-1.HSP70-1	heat shock cognate protein 70-1
Pp3c11_9630	AT1G74270	NaN	Ribosomal protein L35Ae family protein
Pp3c11_10490	AT5G59850	NaN	Ribosomal protein S8 family protein
Pp3c11_10500	AT2G16250	NaN	Leucine-rich repeat protein kinase family protein
Pp3c11_11070	AT5G02960	NaN	Ribosomal protein S12/S23 family protein
Pp3c11_12320	AT2G42640	NaN	Mitogen activated protein kinase kinase kinase-related
Pp3c11_17230	AT4G34670	NaN	Ribosomal protein S3Ae
Pp3c11_17260	AT4G34670	NaN	Ribosomal protein S3Ae

Pp3.3 gene*	Best hit At gene*	At_symbol*	At_description*
Pp3c11_17280	AT5G48760	NaN	Ribosomal protein L13 family protein
Pp3c11_18690	AT4G18330	NaN	Translation elongation factor EF1A/initiation factor IF2gamma family protein
Pp3c11_18980	AT4G36180	NaN	Leucine-rich receptor-like protein kinase family protein
Pp3c11_19460	AT5G05560	EMB2771	E3 ubiquitin ligase. putative
Pp3c11_19990	AT2G39260	NaN	binding;RNA binding
Pp3c11_20050	AT1G01230	NaN	ORMDL family protein
Pp3c11_21120	AT5G10360	EMB3010.RPS6B	Ribosomal protein S6e
Pp3c11_21850	AT1G69220	SIK1	Protein kinase superfamily protein
Pp3c11_22640	AT5G35530	NaN	Ribosomal protein S3 family protein
Pp3c11_22980	AT3G13920	EIF4A1.RH4.TIF4A1	eukaryotic translation initiation factor 4A1
Pp3c11_23980	AT5G55830	NaN	Concanavalin A-like lectin protein kinase family protein
Pp3c11_24300	AT3G10940	NaN	dual specificity protein phosphatase (DsPTP1) family protein
Pp3c11_24610	AT1G67840	CSK	chloroplast sensor kinase
Pp3c11_25130	AT4G18100	NaN	Ribosomal protein L32e
Pp3c11_25960	AT5G25780	ATEIF3B-2.EIF3B.EIF3B-2	eukaryotic translation initiation factor 3B-2
Pp3c11_26050	AT3G12170	NaN	Chaperone DnaJ-domain superfamily protein
Pp3c12_3210	AT2G32060	NaN	Ribosomal protein L7Ae/L30e/S12e/Gadd45 family protein
Pp3c12_5640	AT2G47110	UBQ6	ubiquitin 6
Pp3c12_7550	AT5G26360	NaN	TCP-1/cpn60 chaperonin family protein
Pp3c12_7770	AT2G34480	NaN	Ribosomal protein L18ae/LX family protein
Pp3c12_8050	AT5G67510	NaN	Translation protein SH3-like family protein
Pp3c12_9180	AT5G09500	NaN	Ribosomal protein S19 family protein
Pp3c12_9190	AT5G15200	NaN	Ribosomal protein S4
Pp3c12_9320	AT1G57860	NaN	Translation protein SH3-like family protein
Pp3c12_10200	AT4G36130	NaN	Ribosomal protein L2 family
Pp3c12_10760	AT5G28060	NaN	Ribosomal protein S24e family protein
Pp3c12_10870	AT5G28060	NaN	Ribosomal protein S24e family protein
Pp3c12_14600	AT4G24820	NaN	26S proteasome. regulatory subunit Rpn7;Proteasome component (PCI) domain
Pp3c12_17640	AT4G36420	NaN	Ribosomal protein L12 family protein
Pp3c12_17990	AT3G59540	NaN	Ribosomal L38e protein family
Pp3c12_18200	AT4G05320	UBQ10	polyubiquitin 10
Pp3c12_18260	AT5G59240	NaN	Ribosomal protein S8e family protein
Pp3c12_18499	AT5G26710	NaN	Glutamyl/glutaminyl-tRNA synthetase. class Ic
Pp3c12_18590	AT4G34670	NaN	Ribosomal protein S3Ae
Pp3c12_19360	AT3G55280	RPL23AB	ribosomal protein L23AB
Pp3c12_20520	AT3G11710	ATKRS-1	lysyl-tRNA synthetase 1
Pp3c12_21080	AT4G14570	NaN	acylaminoacyl-peptidase-related
Pp3c12_21880	AT5G12180	CPK17	calcium-dependent protein kinase 17
Pp3c12_22440	AT2G04030	AtHsp90.5.CR88.EMB1956.Hsp88.1.HSP90.5	Chaperone protein htpG family protein
Pp3c12_23350	AT5G05230	NaN	RING/U-box superfamily protein
Pp3c12_25120	AT5G02960	NaN	Ribosomal protein S12/S23 family protein
Pp3c12_25160	AT1G23100	NaN	GroES-like family protein
Pp3c12_25650	AT5G55830	NaN	Concanavalin A-like lectin protein kinase family protein
Pp3c13_1020	AT5G40200	DegP9	DegP protease 9
Pp3c13_1280	AT5G57020	ATNMT1.NMT1	myristoyl-CoA:protein N-myristoyltransferase
Pp3c13_2360	AT1G57660	NaN	Translation protein SH3-like family protein
Pp3c13_4100	AT3G50530	CRK	CDPK-related kinase
Pp3c13_5300	AT5G13120	ATCYP20-2.CYP20-2	cyclophilin 20-2
Pp3c13_5760	AT5G67320	HOS15	WD-40 repeat family protein
Pp3c13_5790	AT4G26100	CK1.CKL1	casein kinase 1
Pp3c13_8360	AT1G41880	NaN	Ribosomal protein L35Ae family protein
Pp3c13_10770	AT2G36620	RPL24A	ribosomal protein L24
Pp3c13_10890	AT3G56150	ATEIF3C-1.ATTIF3C1.EIF3C.EIF3C-1.TIF3C1	eukaryotic translation initiation factor 3C
Pp3c13_12320	AT2G34480	NaN	Ribosomal protein L18ae/LX family protein
Pp3c13_13360	AT5G65700	BAM1	Leucine-rich receptor-like protein kinase family protein
Pp3c13_13363	AT2G04520	NaN	Nucleic acid-binding. OB-fold-like protein
Pp3c13_13620	AT2G04520	NaN	Nucleic acid-binding. OB-fold-like protein
Pp3c13_13900	AT5G26860	LON1.LON_ARA_ARA	lon protease 1
Pp3c13_14270	AT5G61790	ATCNX1.CNX1	calnexin 1
Pp3c13_14280	AT5G61790	ATCNX1.CNX1	calnexin 1
Pp3c13_15330	AT2G20060	NaN	Ribosomal protein L4/L1 family
Pp3c13_15620	AT1G69410	ATELF5A-3.ELF5A-3	eukaryotic elongation factor 5A-3
Pp3c13_15900	AT3G49010	ATBBC1.BBC1.RSU2	breast basic conserved 1
Pp3c13_15920	AT3G55280	RPL23AB	ribosomal protein L23AB
Pp3c13_19780	AT5G15200	NaN	Ribosomal protein S4
Pp3c13_20020	AT5G59240	NaN	Ribosomal protein S8e family protein
Pp3c13_20230	AT4G38130	ATHD1.ATHDA19.HD1.HDA1.HDA19.RPD3A	histone deacetylase 1
Pp3c13_21610	AT1G51980	NaN	Insulinase (Peptidase family M16) protein
Pp3c13_21760	AT4G36130	NaN	Ribosomal protein L2 family
Pp3c13_22220	AT5G28060	NaN	Ribosomal protein S24e family protein
Pp3c13_23560	AT3G14400	UBP25	ubiquitin-specific protease 25
Pp3c13_23630	AT4G36130	NaN	Ribosomal protein L2 family
Pp3c13_24420	AT3G62870	NaN	Ribosomal protein L7Ae/L30e/S12e/Gadd45 family protein
Pp3c13_24450	AT3G62870	NaN	Ribosomal protein L7Ae/L30e/S12e/Gadd45 family protein
Pp3c14_1610	AT5G56890	NaN	Protein kinase superfamily protein
Pp3c14_1810	AT4G25740	NaN	RNA binding Plectin/S10 domain-containing protein
Pp3c14_3350	AT2G16920	PFU2.UBC23	ubiquitin-conjugating enzyme 23
Pp3c14_3360	AT4G24190	AtHsp90-7.AtHsp90.7.HSP90.7.SHD	Chaperone protein htpG family protein
Pp3c14_3490	AT3G49010	ATBBC1.BBC1.RSU2	breast basic conserved 1
Pp3c14_4100	AT1G74060	NaN	Ribosomal protein L6 family protein
Pp3c14_4160	AT1G74060	NaN	Ribosomal protein L6 family protein
Pp3c14_7180	AT3G45030	NaN	Ribosomal protein S10p/S20e family protein
Pp3c14_7440	AT1G05180	AXR1	NAD(P)-binding Rossmann-fold superfamily protein
Pp3c14_7550	AT2G19740	NaN	Ribosomal protein L31e family protein
Pp3c14_8600	AT1G33140	PGY2	Ribosomal protein L6 family
Pp3c14_9600	AT3G48930	EMB1080	Nucleic acid-binding. OB-fold-like protein
Pp3c14_10580	AT4G18100	NaN	Ribosomal protein L32e
Pp3c14_12590	AT5G58290	RPT3	regulatory particle triple-A ATPase 3
Pp3c14_13580	AT4G39200	NaN	Ribosomal protein S25 family protein

Pp3.3 gene*	Best hit At gene*	At_symbol*	At_description*
Pp3c14_17270	AT1G52600	NaN	Peptidase S24/S26A/S26B/S26C family protein
Pp3c14_18020	AT1G75950	ASK1.ATSKP1.SKP1.SKP1A.UIP1	S phase kinase-associated protein 1
Pp3c14_19610	AT5G03850	NaN	Nucleic acid-binding. OB-fold-like protein
Pp3c14_19640	AT3G02080	NaN	Ribosomal protein S19e family protein
Pp3c14_20220	AT5G57870	eIFiso4G1	MIF4G domain-containing protein / MA3 domain-containing protein
Pp3c14_20670	AT2G26100	NaN	Galactosyltransferase family protein
Pp3c14_21510	AT5G24510	NaN	60S acidic ribosomal protein family
Pp3c14_22830	AT5G56280	CSN6A	COP9 signalosome subunit 6A
Pp3c14_22920	AT4G16720	NaN	Ribosomal protein L23/L15e family protein
Pp3c14_24450	AT5G53300	UBC10	ubiquitin-conjugating enzyme 10
Pp3c14_26580	AT5G55500	ATXYLT.XYLT	beta-1.2-xylosyltransferase
Pp3c15_290	AT3G62870	NaN	Ribosomal protein L7Ae/L30e/S12e/Gadd45 family protein
Pp3c15_2200	AT2G26990	ATCSN2.COP12.CSN2.FUS12	proteasome family protein
Pp3c15_2560	AT1G04870	ATPRMT10.PRMT10	protein arginine methyltransferase 10
Pp3c15_4020	AT2G31170	SYCO ARATH	Cysteinyl-tRNA synthetase. class Ia family protein
Pp3c15_4270	AT5G56000	AtHsp90.4.Hsp81.4	HEAT SHOCK PROTEIN 81.4
Pp3c15_4370	AT1G15250	NaN	Zinc-binding ribosomal protein family protein
Pp3c15_5130	AT3G16780	NaN	Ribosomal protein L19e family protein
Pp3c15_5860	AT4G27090	NaN	Ribosomal protein L14
Pp3c15_10750	AT2G45270	GCP1	glycoprotease 1
Pp3c15_10960	AT4G17910	NaN	transferases. transferring acyl groups
Pp3c15_12260	AT1G61770	NaN	Chaperone DnaJ-domain superfamily protein
Pp3c15_12700	AT2G16485	NaN	nucleic acid binding;zinc ion binding;DNA binding
Pp3c15_13410	AT3G03960	NaN	TCP-1/cpn60 chaperonin family protein
Pp3c15_14410	AT3G53430	NaN	Ribosomal protein L11 family protein
Pp3c15_14860	AT4G31985	NaN	Ribosomal protein L39 family protein
Pp3c15_19180	AT1G71810	NaN	Protein kinase superfamily protein
Pp3c15_19200	AT5G56740	HAC07.HAC7.HAG02.HAG2	histone acetyltransferase of the GNAT family 2
Pp3c15_20280	AT5G47750	D6PKL2.PK5	D6 protein kinase like 2
Pp3c15_21360	AT5G35530	NaN	Ribosomal protein S3 family protein
Pp3c15_21510	AT3G45030	NaN	Ribosomal protein S10p/S20e family protein
Pp3c15_22100	AT1G02090	ATCSN7.COP15.CSN7.FUS5	Proteasome component (PCI) domain protein
Pp3c15_22240	AT3G11510	NaN	Ribosomal protein S11 family protein
Pp3c15_24030	AT3G16560	NaN	Protein phosphatase 2C family protein
Pp3c15_24250	AT5G57610	NaN	Protein kinase superfamily protein with octicosapeptide/Phox/Bem1p domain
Pp3c15_25430	AT5G10360	EMB3010.RPS6B	Ribosomal protein S6e
Pp3c15_25780	AT5G10360	EMB3010.RPS6B	Ribosomal protein S6e
Pp3c15_25810	AT4G31700	RPS6.RPS6A	ribosomal protein S6
Pp3c16_410	AT3G16780	NaN	Ribosomal protein L19e family protein
Pp3c16_2090	AT1G34030	NaN	Ribosomal protein S13/S18 family
Pp3c16_3030	AT3G47890	NaN	Ubiquitin carboxyl-terminal hydrolase-related protein
Pp3c16_3760	AT5G35530	NaN	Ribosomal protein S3 family protein
Pp3c16_4180	AT4G30890	UBP24	ubiquitin-specific protease 24
Pp3c16_7870	AT4G32300	SD2-5	S-domain-2 5
Pp3c16_7980	AT2G32060	NaN	Ribosomal protein L7Ae/L30e/S12e/Gadd45 family protein
Pp3c16_8860	AT1G14000	VIK	VH1-interacting kinase
Pp3c16_9210	AT2G40510	NaN	Ribosomal protein S26e family protein
Pp3c16_9340	AT5G06410	NaN	DNAJ heat shock N-terminal domain-containing protein
Pp3c16_10400	AT1G77940	NaN	Ribosomal protein L7Ae/L30e/S12e/Gadd45 family protein
Pp3c16_10880	AT3G45100	SETH2	UDP-Glycosyltransferase superfamily protein
Pp3c16_12160	AT5G09500	NaN	Ribosomal protein S19 family protein
Pp3c16_13640	AT3G04400	emb2171	Ribosomal protein L14p/L23e family protein
Pp3c16_15220	AT4G00100	ATRPS13A.PFL2.RPS13.RPS13A	ribosomal protein S13A
Pp3c16_16110	AT5G05780	AE3.ATHMOV34.RPN8A	RP non-ATPase subunit 8A
Pp3c16_16670	AT3G25660	NaN	Amidase family protein
Pp3c16_16960	AT3G60820	PBF1	N-terminal nucleophile aminohydrolases (Ntn hydrolases) superfamily protein
Pp3c16_17660	AT3G02090	MPPBETA	Insulinase (Peptidase family M16) protein
Pp3c16_18820	AT2G22360	NaN	DNAJ heat shock family protein
Pp3c16_18880	AT5G58420	NaN	Ribosomal protein S4 (RPS4A) family protein
Pp3c16_20070	AT4G10450	NaN	Ribosomal protein L6 family
Pp3c16_22430	AT1G70600	NaN	Ribosomal protein L18e/L15 superfamily protein
Pp3c16_23890	AT1G47250	PAF2	20S proteasome alpha subunit F2
Pp3c17_1180	AT2G42620	MAX2.ORE9.PPS	RNI-like superfamily protein
Pp3c17_2160	AT5G39850	NaN	Ribosomal protein S4
Pp3c17_2480	AT3G10660	ATCPK2.CPK2	calmodulin-domain protein kinase cdpk isoform 2
Pp3c17_2560	AT3G53150	NaN	DNAJ heat shock N-terminal domain-containing protein
Pp3c17_3630	AT2G32060	NaN	Ribosomal protein L7Ae/L30e/S12e/Gadd45 family protein
Pp3c17_4670	AT4G39200	NaN	Ribosomal protein S25 family protein
Pp3c17_5990	AT5G49030	OVA2	tRNA synthetase class I (I. L. M and V) family protein
Pp3c17_7960	AT4G37040	MAP1D	methionine aminopeptidase 1D
Pp3c17_7990	AT4G30610	BRS1.SCPL24	alpha/beta-Hydrolases superfamily protein
Pp3c17_12020	AT3G26618	ERF1-3	eukaryotic release factor 1-3
Pp3c17_13270	AT1G05170	NaN	Galactosyltransferase family protein
Pp3c17_13300	AT5G03850	NaN	Nucleic acid-binding. OB-fold-like protein
Pp3c17_13610	AT1G34130	STT3B	staurosporin and temperature sensitive 3-like b
Pp3c17_15200	AT3G02080	NaN	Ribosomal protein S19e family protein
Pp3c17_18440	AT5G57870	eIFiso4G1	MIF4G domain-containing protein / MA3 domain-containing protein
Pp3c17_18640	AT5G57870	eIFiso4G1	MIF4G domain-containing protein / MA3 domain-containing protein
Pp3c17_18740	AT5G57870	eIFiso4G1	MIF4G domain-containing protein / MA3 domain-containing protein
Pp3c17_20640	AT3G14290	PAE2	20S proteasome alpha subunit E2
Pp3c17_22180	AT4G16720	NaN	Ribosomal protein L23/L15e family protein
Pp3c17_22190	AT5G24510	NaN	60S acidic ribosomal protein family
Pp3c17_23040	AT5G59140	NaN	BTB/POZ domain-containing protein
Pp3c18_1350	AT5G18380	NaN	Ribosomal protein S5 domain 2-like superfamily protein
Pp3c18_2090	AT4G34670	NaN	Ribosomal protein S3Ae
Pp3c18_2250	AT3G59990	MAP2B	methionine aminopeptidase 2B
Pp3c18_2370	AT3G59990	MAP2B	methionine aminopeptidase 2B
Pp3c18_3220	AT5G22060	ATJ2.J2	DNAJ homologue 2
Pp3c18_9650	AT5G66140	PAD2	proteasome alpha subunit D2

Pp3.3 gene*	Best hit At gene*	At_symbol*	At_description*
Pp3c18_13160	AT1G15250	NaN	Zinc-binding ribosomal protein family protein
Pp3c18_13650	AT1G56450	PBG1	20S proteasome beta subunit G1
Pp3c18_13860	AT2G43460	NaN	Ribosomal L38e protein family
Pp3c18_14410	AT2G26730	NaN	Leucine-rich repeat protein kinase family protein
Pp3c18_14440	AT3G16780	NaN	Ribosomal protein L19e family protein
Pp3c18_14720	AT4G33250	ATTIF3K1.EIF3K.TIF3K1	eukaryotic translation initiation factor 3K
Pp3c18_20060	AT1G09640	NaN	Translation elongation factor EF1B. gamma chain
Pp3c18_21660	AT5G67530	ATPUB49.PUB49	plant U-box 49
Pp3c18_21730	AT1G67510	NaN	Leucine-rich repeat protein kinase family protein
Pp3c19_1620	AT4G01037	AtWTF1.WTF1	Ubiquitin carboxyl-terminal hydrolase family protein
Pp3c19_4350	AT2G07340	PFD1	PREFOLDIN 1
Pp3c19_5590	AT5G19180	ECR1	E1 C-terminal related 1
Pp3c19_5720	AT3G13490	ATKRS-2.OVA5	Lysyl-tRNA synthetase. class II
Pp3c19_6490	AT5G59240	NaN	Ribosomal protein S8e family protein
Pp3c19_7230	AT4G25890	NaN	60S acidic ribosomal protein family
Pp3c19_10310	AT3G04840	NaN	Ribosomal protein S3Ae
Pp3c19_10330	AT5G48760	NaN	Ribosomal protein L13 family protein
Pp3c19_12590	AT1G17720	ATB BETA	Protein phosphatase 2A. regulatory subunit PR55
Pp3c19_13120	AT1G72550	NaN	tRNA synthetase beta subunit family protein
Pp3c19_15000	AT2G04030	AtHsp90.5.CR88.EMB1956.Hsp88.1.HSP90.5	Chaperone protein htpG family protein
Pp3c19_17130	AT3G20630	ATUBP14.PER1.TTN6.UBP14	ubiquitin-specific protease 14
Pp3c19_17550	AT1G09640	NaN	Translation elongation factor EF1B. gamma chain
Pp3c19_18770	AT5G67360	ARA12	Subtilase family protein
Pp3c19_20260	AT2G20580	ATRPN1A.RPN1A	26S proteasome regulatory subunit S2 1A
Pp3c19_21200	AT1G70190	NaN	Ribosomal protein L7/L12. C-terminal/adaptor protein ClpS-like
Pp3c19_22410	AT5G18380	NaN	Ribosomal protein S5 domain 2-like superfamily protein
Pp3c20_1360	AT1G48830	NaN	Ribosomal protein S7e family protein
Pp3c20_1830	AT1G16740	NaN	Ribosomal protein L20
Pp3c20_2550	AT1G52300	NaN	Zinc-binding ribosomal protein family protein
Pp3c20_2940	AT5G53340	NaN	Galactosyltransferase family protein
Pp3c20_5690	AT3G57290	ATEIF3E-1.ATINT6.EIF3E.INT-6.INT6.TIF3E1	eukaryotic translation initiation factor 3E
Pp3c20_8320	AT5G48030	GFA2	gametophytic factor 2
Pp3c20_10410	AT5G35980	YAK1	yeast YAK1-related gene 1
Pp3c20_12680	AT2G42710	NaN	Ribosomal protein L1p/L10e family
Pp3c20_15150	AT2G34480	NaN	Ribosomal protein L18ae/LX family protein
Pp3c20_17290	AT5G54380	THE1	protein kinase family protein
Pp3c20_18180	AT3G22630	PBD1.PRCGB	20S proteasome beta subunit D1
Pp3c20_18740	AT3G57490	NaN	Ribosomal protein S5 family protein
Pp3c20_18830	AT1G70600	NaN	Ribosomal protein L18e/L15 superfamily protein
Pp3c20_18970	AT2G37270	ATRPS5B.RPS5B	ribosomal protein 5B
Pp3c20_19020	AT5G26830	NaN	Threonyl-tRNA synthetase
Pp3c20_20510	AT1G79930	HSP91	heat shock protein 91
Pp3c20_22360	AT3G51270	NaN	protein serine/threonine kinases;ATP binding;catalytics
Pp3c20_22800	AT1G79940	ATERDJ2A	DnaJ / Sec63 Brl domains-containing protein
Pp3c20_23170	AT3G52590	EMB2167.ERD16.HAP4.UBQ1	ubiquitin extension protein 1
Pp3c20_23570	AT1G24510	NaN	TCP-1/cpn60 chaperonin family protein
Pp3c21_580	AT5G52520	OVA6.PRORS1	Class II aaRS and biotin synthetases superfamily protein
Pp3c21_1460	AT1G71230	AJH2.CSN5.CSN5B	COP9-signalosome 5B
Pp3c21_5070	AT3G24190	NaN	Protein kinase superfamily protein
Pp3c21_6620	AT5G59240	NaN	Ribosomal protein S8e family protein
Pp3c21_8410	AT3G50110	ATPEN3.PEN3	PTEN 3
Pp3c21_8670	AT1G48520	GATB	GLU-ADT subunit B
Pp3c21_8680	AT2G24090	NaN	Ribosomal protein L35
Pp3c21_10350	AT5G19180	ECR1	E1 C-terminal related 1
Pp3c21_10940	AT1G56350	NaN	Peptide chain release factor 2
Pp3c21_11200	AT2G26730	NaN	Leucine-rich repeat protein kinase family protein
Pp3c21_11230	AT3G16780	NaN	Ribosomal protein L19e family protein
Pp3c21_14550	AT1G09640	NaN	Translation elongation factor EF1B. gamma chain
Pp3c21_16160	AT4G25890	NaN	60S acidic ribosomal protein family
Pp3c21_20630	AT5G18380	NaN	Ribosomal protein S5 domain 2-like superfamily protein
Pp3c21_21510	AT4G34670	NaN	Ribosomal protein S3Ae
Pp3c21_21540	AT3G61110	ARS27A.RS27A	ribosomal protein S27
Pp3c21_21800	AT4G26100	CK1.CKL1	casein kinase 1
Pp3c21_22170	AT2G45240	MAP1A	methionine aminopeptidase 1A
Pp3c21_22630	AT2G45240	MAP1A	methionine aminopeptidase 1A
Pp3c22_1770	AT1G29990	PFD6	prefoldin 6
Pp3c22_1860	AT5G59240	NaN	Ribosomal protein S8e family protein
Pp3c22_2590	AT3G04240	SEC	Tetratricopeptide repeat (TPR)-like superfamily protein
Pp3c22_4190	AT5G51880	NaN	2-oxoglutarate (2OG) and Fe(II)-dependent oxygenase superfamily protein
Pp3c22_5470	AT5G16710	DHAR3	dehydroascorbate reductase 1
Pp3c22_12710	AT4G23570	SGT1A	phosphatase-related
Pp3c22_15010	AT1G09640	NaN	Translation elongation factor EF1B. gamma chain
Pp3c22_15340	AT1G72550	NaN	tRNA synthetase beta subunit family protein
Pp3c22_15620	AT5G53920	NaN	ribosomal protein L11 methyltransferase-related
Pp3c22_17210	AT5G22060	ATJ2.J2	DNAJ homologue 2
Pp3c22_18300	AT4G34670	NaN	Ribosomal protein S3Ae
Pp3c22_19100	AT5G50430	UBC33	ubiquitin-conjugating enzyme 33
Pp3c22_21090	AT5G18380	NaN	Ribosomal protein S5 domain 2-like superfamily protein
Pp3c22_22010	AT1G66580	RPL10C.SAG24	senescence associated gene 24
Pp3c23_2850	AT5G13120	ATCYP20-2.CYP20-2	cyclophilin 20-2
Pp3c23_2910	AT3G18210	NaN	2-oxoglutarate (2OG) and Fe(II)-dependent oxygenase superfamily protein
Pp3c23_3680	AT1G51590	MANIB.MNS1	alpha-mannosidase 1
Pp3c23_4120	AT1G07320	RPL4	ribosomal protein L4
Pp3c23_4520	AT3G04400	emb2171	Ribosomal protein L14p/L23e family protein
Pp3c23_6150	AT2G37270	ATRPS5B.RPS5B	ribosomal protein 5B
Pp3c23_8220	AT1G23290	RPL27A.RPL27AB	Ribosomal protein L18e/L15 superfamily protein
Pp3c23_8410	AT3G57490	NaN	Ribosomal protein S5 family protein
Pp3c23_10040	AT5G27700	NaN	Ribosomal protein S21e
Pp3c23_12730	AT1G08360	NaN	Ribosomal protein L1p/L10e family

Pp3.3 gene*	Best hit At gene*	At_symbol*	At_description*
Pp3c23_14790	AT3G24590	PLSP1	plastidic type i signal peptidase 1
Pp3c23_15260	AT1G14650	NaN	SWAP (Suppressor-of-White-APricot)/surp domain-containing protein/ ubiquitin family protein
Pp3c23_15370	AT5G48030	GFA2	gametophytic factor 2
Pp3c23_15390	AT5G26710	NaN	Glutamyl/glutaminyl-tRNA synthetase. class Ic
Pp3c23_17260	AT1G52300	NaN	Zinc-binding ribosomal protein family protein
Pp3c23_17430	AT3G55620	emb1624	Translation initiation factor IF6
Pp3c23_18100	AT5G24510	NaN	60S acidic ribosomal protein family
Pp3c23_18210	AT3G17465	RPL3P	ribosomal protein L3 plastid
Pp3c23_18280	AT1G48830	NaN	Ribosomal protein S7e family protein
Pp3c23_18930	AT5G04870	ATCPK1.CPK1	calcium dependent protein kinase 1
Pp3c23_22060	AT2G20450	NaN	Ribosomal protein L14
Pp3c24_20	AT5G14660	ATDEF2.DEF2.PDF1B	peptide deformylase 1B
Pp3c24_430	AT3G52590	EMB2167.ERD16.HAP4.UBQ1	ubiquitin extension protein 1
Pp3c24_1760	AT1G79930	HSP91	heat shock protein 91
Pp3c24_2340	AT4G25740	NaN	RNA binding Plectin/S10 domain-containing protein
Pp3c24_3690	AT1G08360	NaN	Ribosomal protein L1p/L10e family
Pp3c24_4740	AT3G46510	ATPUB13.PUB13	plant U-box 13
Pp3c24_5500	AT3G57490	NaN	Ribosomal protein S5 family protein
Pp3c24_5780	AT3G57490	NaN	Ribosomal protein S5 family protein
Pp3c24_6930	AT5G66680	DGL1	dolichyl-diphosphooligosaccharide-protein glycosyltransferase 48kDa subunit family protein
Pp3c24_8160	AT5G27700	NaN	Ribosomal protein S21e
Pp3c24_9660	AT5G13120	ATCYP20-2.CYP20-2	cyclophilin 20-2
Pp3c24_12180	AT2G28000	CH-CPN60A.CPN60A.SLP	chaperonin-60alpha
Pp3c24_12530	AT4G11630	NaN	Ribosomal protein L19 family protein
Pp3c24_13910	AT3G55620	emb1624	Translation initiation factor IF6
Pp3c24_15540	AT4G23620	NaN	Ribosomal protein L25/Gln-tRNA synthetase. anti-codon-binding domain
Pp3c24_16310	AT3G17850	NaN	Protein kinase superfamily protein
Pp3c24_16740	AT2G29960	ATCYP5.CYP19-4.CYP5	cyclophilin 5
Pp3c24_18220	AT5G16130	NaN	Ribosomal protein S7e family protein
Pp3c24_18680	AT3G01800	NaN	Ribosome recycling factor
Pp3c24_18790	AT2G34480	NaN	Ribosomal protein L18ae/LX family protein
Pp3c24_18880	AT2G37270	ATRPS5B.RPS5B	ribosomal protein 5B
Pp3c24_20890	AT4G27090	NaN	Ribosomal protein L14
Pp3c24_20920	AT5G42400	ATXR7.SDG25	SET domain protein 25
Pp3c25_230	AT5G18790	NaN	Ribosomal protein L33 family protein
Pp3c25_2440	AT1G10840	TIF3H1	translation initiation factor 3 subunit H1
Pp3c25_2670	AT1G10840	TIF3H1	translation initiation factor 3 subunit H1
Pp3c25_3200	AT1G09730	NaN	Cysteine proteinases superfamily protein
Pp3c25_3730	AT5G58420	NaN	Ribosomal protein S4 (RPS4A) family protein
Pp3c25_3736	AT5G58420	NaN	Ribosomal protein S4 (RPS4A) family protein
Pp3c25_4190	AT3G05530	ATS6A.2.RPT5A	regulatory particle triple-A ATPase 5A
Pp3c25_5290	AT4G32300	SD2-5	S-domain-2 5
Pp3c25_8110	AT4G08350	GTA02.GTA2	global transcription factor group A2
Pp3c25_9320	AT1G77940	NaN	Ribosomal protein L7Ae/L30e/S12e/Gadd45 family protein
Pp3c25_9390	AT3G22630	PBD1.PRCGB	20S proteasome beta subunit D1
Pp3c25_10250	AT1G06840	NaN	Leucine-rich repeat protein kinase family protein
Pp3c25_11980	AT1G26880	NaN	Ribosomal protein L34e superfamily protein
Pp3c25_13260	AT1G70600	NaN	Ribosomal protein L18e/L15 superfamily protein
Pp3c26_330	AT3G62870	NaN	Ribosomal protein L7Ae/L30e/S12e/Gadd45 family protein
Pp3c26_350	AT2G47610	NaN	Ribosomal protein L7Ae/L30e/S12e/Gadd45 family protein
Pp3c26_360	AT3G62870	NaN	Ribosomal protein L7Ae/L30e/S12e/Gadd45 family protein
Pp3c26_2200	AT5G55140	NaN	ribosomal protein L30 family protein
Pp3c26_3000	AT5G27700	NaN	Ribosomal protein S21e
Pp3c26_4540	AT3G50530	CRK	CDPK-related kinase
Pp3c26_8120	AT5G62710	NaN	Leucine-rich repeat protein kinase family protein
Pp3c26_9050	AT5G10080	NaN	Eukaryotic aspartyl protease family protein
Pp3c26_9350	AT5G10080	NaN	Eukaryotic aspartyl protease family protein
Pp3c26_10900	AT5G18110	NCBP	novel cap-binding protein
Pp3c26_11220	AT4G14320	NaN	Zinc-binding ribosomal protein family protein
Pp3c26_11290	AT4G11420	ATEIF3A-1.ATTIF3A1.EIF3A.EIF3A-1.TIF3A1	eukaryotic translation initiation factor 3A
Pp3c26_13720	AT5G02610	NaN	Ribosomal L29 family protein
Pp3c26_15390	AT3G10950	NaN	Zinc-binding ribosomal protein family protein
Pp3c27_190	AT5G20920	EIF2 BETA.EMB1401	eukaryotic translation initiation factor 2 beta subunit
Pp3c27_1320	AT5G35530	NaN	Ribosomal protein S3 family protein
Pp3c27_1920	AT5G15200	NaN	Ribosomal protein S4
Pp3c27_1950	AT3G25660	NaN	Amidase family protein
Pp3c27_2260	AT5G45775	NaN	Ribosomal L5P family protein
Pp3c27_2780	AT3G02080	NaN	Ribosomal protein S19e family protein
Pp3c27_2790	AT1G34360	NaN	translation initiation factor 3 (IF-3) family protein
Pp3c27_5110	AT1G55490	CPN60B.LEN1	chaperonin 60 beta
Pp3c27_7320	AT1G50200	ACD.ALATS	Alanyl-tRNA synthetase
Pp3c27_7820	AT5G09500	NaN	Ribosomal protein S19 family protein
Pp3c27_8190	AT1G33140	PGY2	Ribosomal protein L6 family
Pp3s93_30	AT3G13920	EIF4A1.RH4.TIF4A1	eukaryotic translation initiation factor 4A1
Pp3s116_30	AT3G48930	EMB1080	Nucleic acid-binding. OB-fold-like protein

*Fourty-six of 697 genes are colored in red as the common DEGs both 1cell-DGE and 5'DGE.

Supplementary Table S10. DEGs in 24 h-high identified using 1cell-DGE categorized by the GO term "biosynthetic process".

Pp3.3 gene*	Best hit At gene*	At_symbol*	At_description*
Pp3c1_420	AT5G56670	NaN	Ribosomal protein S30 family protein
Pp3c1_1000	AT1G21600	PTAC6	plastid transcriptionally active 6
Pp3c1_1260	AT1G19910	ATVHA-C2.AVA-2PE.AVA-P2	ATPase. F0/V0 complex. subunit C protein
Pp3c1_1530	AT3G10950	NaN	Zinc-binding ribosomal protein family protein
Pp3c1_1800	AT1G26880	NaN	Ribosomal protein L34e superfamily protein
Pp3c1_3170	AT1G26910	RPL10B	Ribosomal protein L16p/L10e family protein
Pp3c1_3480	AT3G05590	RPL18	ribosomal protein L18
Pp3c1_4030	AT1G41880	NaN	Ribosomal protein L35Ae family protein
Pp3c1_4290	AT5G59850	NaN	Ribosomal protein S8 family protein
Pp3c1_5320	AT3G44890	RPL9	ribosomal protein L9
Pp3c1_5330	AT3G05590	RPL18	ribosomal protein L18
Pp3c1_6160	AT4G00100	ATRPS13A.PFL2.RPS13.RPS13A	ribosomal protein S13A
Pp3c1_6650	AT1G67430	NaN	Ribosomal protein L22p/L17e family protein
Pp3c1_7091	AT2G30390	ATFC-II.FC-II.FC2	ferrochelatase 2
Pp3c1_7160	AT5G39740	OLI7.RPL5B	ribosomal protein L5 B
Pp3c1_7500	AT3G48680	GAMMA CAL2	gamma carbonic anhydrase-like 2
Pp3c1_7810	AT3G20330	PYRB	PYRIMIDINE B
Pp3c1_7830	AT5G56940	NaN	Ribosomal protein S16 family protein
Pp3c1_8280	AT2G40010	NaN	Ribosomal protein L10 family protein
Pp3c1_8290	AT5G58420	NaN	Ribosomal protein S4 (RPS4A) family protein
Pp3c1_9420	AT4G04320	NaN	malonyl-CoA decarboxylase family protein
Pp3c1_10000	AT1G76490	HMG1.HMGR1	hydroxy methylglutaryl CoA reductase 1
Pp3c1_10010	AT5G64050	ATERS.ERS.OVA3	glutamate tRNA synthetase
Pp3c1_12120	AT1G51650	NaN	ATP synthase epsilon chain. mitochondrial
Pp3c1_12610	AT5G64300	ATGCH.ATRIBA1.GCH.RFD1	GTP cyclohydrolase II
Pp3c1_15830	AT5G48760	NaN	Ribosomal protein L13 family protein
Pp3c1_16170	AT2G36620	RPL24A	ribosomal protein L24
Pp3c1_16250	AT2G43360	BIO2.BIOB	Radical SAM superfamily protein
Pp3c1_16850	AT1G77670	NaN	Pyridoxal phosphate (PLP)-dependent transferases superfamily protein
Pp3c1_18050	AT2G44860	NaN	Ribosomal protein L24e family protein
Pp3c1_18250	AT1G29900	CARB	carbamoyl phosphate synthetase B
Pp3c1_18400	AT4G25740	NaN	RNA binding Plectin/S10 domain-containing protein
Pp3c1_19680	AT1G25350	OVA9	glutamine-tRNA ligase. putative / glutaminyl-tRNA synthetase. putative / GlnRS. putative
Pp3c1_20280	AT4G26900	AT-HF.HISN4	HIS HF
Pp3c1_20870	AT4G34840	ATMTAN2.ATMTN2.MTAN2.MTN2	Phosphorylase superfamily protein
Pp3c1_21860	AT3G45030	NaN	Ribosomal protein S10p/S20e family protein
Pp3c1_22510	AT2G02990	ATRNS1.RNS1	ribonuclease 1
Pp3c1_22820	AT5G27820	NaN	Ribosomal L18p/L5e family protein
Pp3c1_24120	AT1G72370	AP40.P40.RP40.RPSAA	40s ribosomal protein SA
Pp3c1_24300	AT1G74050	NaN	Ribosomal protein L6 family protein
Pp3c1_26280	AT3G49010	ATBBC1.BBC1.RSU2	breast basic conserved 1
Pp3c1_28770	AT3G10090	NaN	Nucleic acid-binding. OB-fold-like protein
Pp3c1_29390	AT4G26310	NaN	elongation factor P (EF-P) family protein
Pp3c1_29840	AT5G22130	PNT1	mannosyltransferase family protein
Pp3c1_30660	AT5G28840	GME	GDP-D-mannose 3',5'-epimerase
Pp3c1_31540	AT5G19220	ADG2.APL1	ADP glucose pyrophosphorylase large subunit 1
Pp3c1_32030	AT1G23820	SPDS1	spermidine synthase 1
Pp3c1_32090	AT5G58270	ATATM3.ATM3.STA1	ABC transporter of the mitochondrion 3
Pp3c1_32300	AT4G35800	NRPB1.RNA_POL_II_LS.RPB1	RNA polymerase II large subunit
Pp3c1_32410	AT1G26740	NaN	Ribosomal L32p protein family
Pp3c1_32490	AT4G18100	NaN	Ribosomal protein L32e
Pp3c1_32870	AT2G07690	MCM5	Minichromosome maintenance (MCM2/3/5) family protein
Pp3c1_33150	AT4G24830	NaN	arginosuccinate synthase family
Pp3c1_34100	AT5G37510	CI76.EMB1467	NADH-ubiquinone dehydrogenase. mitochondrial. putative
Pp3c1_34140	AT5G37510	CI76.EMB1467	NADH-ubiquinone dehydrogenase. mitochondrial. putative
Pp3c1_34390	AT2G19740	NaN	Ribosomal protein L31e family protein
Pp3c1_36220	AT5G24510	NaN	60S acidic ribosomal protein family
Pp3c1_37420	AT4G16720	NaN	Ribosomal protein L23/L15e family protein
Pp3c1_39880	AT5G62620	NaN	Galactosyltransferase family protein
Pp3c1_40490	AT5G46160	NaN	Ribosomal protein L14p/L23e family protein
Pp3c1_41090	AT3G23510	NaN	Cyclopropane-fatty-acyl-phospholipid synthase
Pp3c1_41250	AT3G03050	ATCSLD3.CSLD3.KJK	cellulose synthase-like D3
Pp3c1_41400	AT3G03050	ATCSLD3.CSLD3.KJK	cellulose synthase-like D3
Pp3c1_41710	AT5G57500	NaN	Galactosyltransferase family protein
Pp3c2_1270	AT4G23100	ATECS1.CAD2.GSH1.GSHA.PAD2.RML1	glutamate-cysteine ligase
Pp3c2_1330	AT3G03050	ATCSLD3.CSLD3.KJK	cellulose synthase-like D3
Pp3c2_3200	AT5G58770	NaN	Undecaprenyl pyrophosphate synthetase family protein
Pp3c2_3850	AT3G62120	NaN	Class II aaRS and biotin synthetases superfamily protein
Pp3c2_5780	AT4G16720	NaN	Ribosomal protein L23/L15e family protein
Pp3c2_6730	AT3G44010	NaN	Ribosomal protein S14p/S29e family protein
Pp3c2_7940	AT5G28840	GME	GDP-D-mannose 3',5'-epimerase
Pp3c2_8420	AT5G61670	NaN	NaN
Pp3c2_9590	AT4G18100	NaN	Ribosomal protein L32e
Pp3c2_9650	AT3G53740	NaN	Ribosomal protein L36e family protein
Pp3c2_9830	AT1G14610	TWN2.VALRS	valyl-tRNA synthetase / valine--tRNA ligase (VALRS)
Pp3c2_10060	AT3G02080	NaN	Ribosomal protein S19e family protein
Pp3c2_11670	AT5G23535	NaN	KOW domain-containing protein
Pp3c2_13300	AT1G74060	NaN	Ribosomal protein L6 family protein
Pp3c2_13690	AT3G48930	EMB1080	Nucleic acid-binding. OB-fold-like protein
Pp3c2_14050	AT1G07270	NaN	Cell division control. Cdc6
Pp3c2_14410	AT1G54250	ATRPABC16.5.NRPB8A.NRPE8A	RNA polymerase Rpb8
Pp3c2_14800	AT4G39200	NaN	Ribosomal protein S25 family protein
Pp3c2_16490	AT2G19740	NaN	Ribosomal protein L31e family protein
Pp3c2_16770	AT4G39280	NaN	phenylalanyl-tRNA synthetase. putative / phenylalanine--tRNA ligase. putative
Pp3c2_18380	AT3G57220	NaN	Glycosyl transferase family 4 protein
Pp3c2_18620	AT4G25740	NaN	RNA binding Plectin/S10 domain-containing protein
Pp3c2_19430	AT1G29900	CARB	carbamoyl phosphate synthetase B
Pp3c2_22010	AT5G23450	ATLCBK1.LCBK1	long-chain base (LCB) kinase 1
Pp3c2_23140	AT1G77670	NaN	Pyridoxal phosphate (PLP)-dependent transferases superfamily protein
Pp3c2_24130	AT5G02960	NaN	Ribosomal protein S12/S23 family protein
Pp3c2_24870	AT1G51650	NaN	ATP synthase epsilon chain. mitochondrial
Pp3c2_24930	AT2G17360	NaN	Ribosomal protein S4 (RPS4A) family protein
Pp3c2_27110	AT1G15390	ATDEF1.PDF1A	peptide deformylase 1A
Pp3c2_28870	AT5G56670	NaN	Ribosomal protein S30 family protein
Pp3c2_29600	AT1G26910	RPL10B	Ribosomal protein L16p/L10e family protein

Pp3.3 gene*	Best hit At gene*	At_symbol*	At_description*
Pp3c2_29840	AT1G67430	NaN	Ribosomal protein L22p/L17e family protein
Pp3c2_30250	AT3G55610	P5CS2	delta 1-pyrroline-5-carboxylate synthase 2
Pp3c2_31540	AT5G39740	OLI7.RPL5B	ribosomal protein L5 B
Pp3c2_32050	AT5G59850	NaN	Ribosomal protein S8 family protein
Pp3c2_32310	AT4G18040	AT.EIF4E1.CUM1.EIF4E.eIF4E1	eukaryotic translation initiation factor 4E
Pp3c2_32510	AT3G05590	RPL18	ribosomal protein L18
Pp3c2_33280	AT4G18730	RPL16B	ribosomal protein L16B
Pp3c2_35700	AT4G31460	NaN	Ribosomal L28 family
Pp3c2_36900	AT1G19910	ATVHA-C2.AVA-2PE.AVA-P2	ATPase. F0/V0 complex. subunit C protein
Pp3c2_37900	AT1G76490	HMG1.HMGR1	hydroxy methylglutaryl CoA reductase 1
Pp3c2_38140	AT5G56940	NaN	Ribosomal protein S16 family protein
Pp3c3_270	AT5G57020	ATNMT1.NMT1	myristoyl-CoA:protein N-myristoyltransferase
Pp3c3_1140	AT1G07770	RPS15A	ribosomal protein S15A
Pp3c3_1620	AT3G10950	NaN	Zinc-binding ribosomal protein family protein
Pp3c3_2280	AT4G12610	ATRAP74.RAP74	transcription activators;transcription initiation factors
Pp3c3_2800	AT5G02610	NaN	Ribosomal L29 family protein
Pp3c3_3050	AT5G07990	CYP75B1.D501.TT7	Cytochrome P450 superfamily protein
Pp3c3_3090	AT3G22300	RPS10	ribosomal protein S10
Pp3c3_3400	AT1G27600	I9H.IRX9-L	Nucleotide-diphospho-sugar transferases superfamily protein
Pp3c3_3480	AT1G74270	NaN	Ribosomal protein L35Ae family protein
Pp3c3_5450	AT1G67350	NaN	NaN
Pp3c3_5550	AT1G57660	NaN	Translation protein SH3-like family protein
Pp3c3_5690	AT4G30930	NFD1	Ribosomal protein L21
Pp3c3_7160	AT2G07560	AHA6.HA6	H(+)-ATPase 6
Pp3c3_7180	AT2G07560	AHA6.HA6	H(+)-ATPase 6
Pp3c3_8500	AT3G56150	ATEIF3C-1.ATTIF3C1.EIF3C.EIF3C-1.TIF3C1	eukaryotic translation initiation factor 3C
Pp3c3_8930	AT3G53020	RPL24.RPL24B.STV1	Ribosomal protein L24e family protein
Pp3c3_8980	AT3G11170	FAD7.FADD	fatty acid desaturase 7
Pp3c3_9940	AT5G64420	NaN	DNA polymerase V family
Pp3c3_10260	AT4G14320	NaN	Zinc-binding ribosomal protein family protein
Pp3c3_10800	AT5G03300	ADK2	adenosine kinase 2
Pp3c3_10840	AT2G36620	RPL24A	ribosomal protein L24
Pp3c3_10859	AT5G28840	GME	GDP-D-mannose 3',5'-epimerase
Pp3c3_11250	AT2G34480	NaN	Ribosomal protein L18ae/LX family protein
Pp3c3_12210	AT2G18020	EMB2296	Ribosomal protein L2 family
Pp3c3_13060	AT1G30820	NaN	CTP synthase family protein
Pp3c3_14800	AT5G60700	NaN	glycosyltransferase family protein 2
Pp3c3_14880	AT5G04800	NaN	Ribosomal S17 family protein
Pp3c3_16990	AT3G54470	NaN	uridine 5'-monophosphate synthase / UMP synthase (PYRE-F) (UMPS)
Pp3c3_19700	AT5G28060	NaN	Ribosomal protein S24e family protein
Pp3c3_20490	AT1G04610	YUC3	YUCCA 3
Pp3c3_21130	AT3G49010	ATBBC1.BBC1.RSU2	breast basic conserved 1
Pp3c3_21830	AT1G74040	IMS1.IPMS2.MAML-3	2-isopropylmalate synthase 1
Pp3c3_23540	AT3G58700	NaN	Ribosomal L5P family protein
Pp3c3_23720	AT5G15200	NaN	Ribosomal protein S4
Pp3c3_23880	AT3G63410	APG1.E37.IEP37.VTE3	S-adenosyl-L-methionine-dependent methyltransferases superfamily protein
Pp3c3_25300	AT2G18110	NaN	Translation elongation factor EF1B/ribosomal protein S6 family protein
Pp3c3_25640	AT2G43460	NaN	Ribosomal L38e protein family
Pp3c3_26540	AT3G25860	LTA2.PLE2	2-oxoacid dehydrogenases acyltransferase family protein
Pp3c3_26860	AT4G36420	NaN	Ribosomal protein L12 family protein
Pp3c3_29280	AT5G20980	ATMS3.MS3	methionine synthase 3
Pp3c3_33160	AT4G34640	ERG9.SQS1	squalene synthase 1
Pp3c3_33470	AT1G25220	ASB1.TRP4.WEI7	anthranilate synthase beta subunit 1
Pp3c3_34710	AT5G03905	NaN	Iron-sulphur cluster biosynthesis family protein
Pp3c3_35300	AT3G16950	LPD1.ptlpd1	lipoamide dehydrogenase 1
Pp3c3_35760	AT5G47320	RPS19	ribosomal protein S19
Pp3c3_36280	AT1G53880	NaN	Eukaryotic translation initiation factor 2B (eIF-2B) family protein
Pp3c3_37040	AT5G20040	ATIPT9.IPT9	isopentenyltransferase 9
Pp3c3_37810	AT2G39630	NaN	Nucleotide-diphospho-sugar transferases superfamily protein
Pp3c4_1020	AT2G22240	ATIPS2.ATMIPS2.MIPS2	myo-inositol-1-phosphate synthase 2
Pp3c4_3220	AT5G27700	NaN	Ribosomal protein S21e
Pp3c4_6350	AT3G49080	NaN	Ribosomal protein S5 domain 2-like superfamily protein
Pp3c4_7510	AT3G11710	ATKRS-1	lysyl-tRNA synthetase 1
Pp3c4_8600	AT3G57560	NAGK	N-acetyl-l-glutamate kinase
Pp3c4_8620	AT3G55280	RPL23AB	ribosomal protein L23AB
Pp3c4_11270	AT4G13430	ATLEUC1.IIL1	isopropyl malate isomerase large subunit 1
Pp3c4_11370	AT5G59240	NaN	Ribosomal protein S8e family protein
Pp3c4_11930	AT2G36170	NaN	Ubiquitin supergroup;Ribosomal protein L40e
Pp3c4_12690	AT4G36130	NaN	Ribosomal protein L2 family
Pp3c4_13370	AT3G58700	NaN	Ribosomal L5P family protein
Pp3c4_13380	AT3G11710	ATKRS-1	lysyl-tRNA synthetase 1
Pp3c4_13590	AT5G03415	ATDPB.DPB	Transcription factor DP
Pp3c4_14160	AT4G34670	NaN	Ribosomal protein S3Ae
Pp3c4_15060	AT4G03550	ATGSL05.ATGSL5.GSL05.GSL5.PMR4	glucan synthase-like 5
Pp3c4_15290	AT5G15200	NaN	Ribosomal protein S4
Pp3c4_15480	AT1G77940	NaN	Ribosomal protein L7Ae/L30e/S12e/Gadd45 family protein
Pp3c4_15740	AT1G15110	NaN	phosphatidyl serine synthase family protein
Pp3c4_16870	AT1G19580	GAMMA CA1	gamma carbonic anhydrase 1
Pp3c4_17740	AT4G02610	NaN	Aldolase-type TIM barrel family protein
Pp3c4_17856	AT3G07160	ATGSL10.CALS9.gsl10	glucan synthase-like 10
Pp3c4_20080	AT5G51460	ATTPPA	Haloacid dehalogenase-like hydrolase (HAD) superfamily protein
Pp3c4_21520	AT1G57860	NaN	Translation protein SH3-like family protein
Pp3c4_21680	AT2G30490	ATC4H.C4H.CYP73A5.REF3	cinnamate-4-hydroxylase
Pp3c4_23720	AT3G62830	ATUXS2.AUD1.UXS2	NAD(P)-binding Rossmann-fold superfamily protein
Pp3c4_24414	AT3G56150	ATEIF3C-1.ATTIF3C1.EIF3C.EIF3C-1.TIF3C1	eukaryotic translation initiation factor 3C
Pp3c4_25990	AT2G38560	TFIIS	transcript elongation factor IIS
Pp3c4_26450	AT4G11420	ATEIF3A-1.ATTIF3A1.EIF3A.EIF3A-1.TIF3A1	eukaryotic translation initiation factor 3A
Pp3c4_27540	AT5G59850	NaN	Ribosomal protein S8 family protein
Pp3c4_29560	AT3G13882	NaN	Ribosomal protein L34
Pp3c4_29970	AT1G74270	NaN	Ribosomal protein L35Ae family protein
Pp3c4_31590	AT4G12110	ATSMO1.ATSMO1-1.SMO1-1	sterol-4alpha-methyl oxidase 1-1
Pp3c5_2800	AT1G34030	NaN	Ribosomal protein S13/S18 family
Pp3c5_3300	AT5G42650	AOS.CYP74A.DDE2	allene oxide synthase
Pp3c5_3870	AT3G58700	NaN	Ribosomal L5P family protein
Pp3c5_4330	AT1G77940	NaN	Ribosomal protein L7Ae/L30e/S12e/Gadd45 family protein
Pp3c5_5330	AT5G35530	NaN	Ribosomal protein S3 family protein

Pp3.3 gene*	Best hit At gene*	At_symbol*	At_description*
Pp3c5_5750	AT1G07770	RPS15A	ribosomal protein S15A
Pp3c5_6000	AT5G03850	NaN	Nucleic acid-binding. OB-fold-like protein
Pp3c5_6600	AT2G13540	ABH1.ATCBP80.CBP80.ENS	ARM repeat superfamily protein
Pp3c5_7330	AT1G55880	NaN	Pyridoxal-5'-phosphate-dependent enzyme family protein
Pp3c5_8810	AT2G36390	BE3.SBE2.1	starch branching enzyme 2.1
Pp3c5_9590	AT2G46210	NaN	Fatty acid/sphingolipid desaturase
Pp3c5_9890	AT2G33840	NaN	Tyrosyl-tRNA synthetase. class Ib. bacterial/mitochondrial
Pp3c5_10340	AT1G78570	ATRHM1.RHM1.ROL1	rhamnose biosynthesis 1
Pp3c5_10680	AT3G20230	NaN	Ribosomal L18p/L5e family protein
Pp3c5_10720	AT1G32930	NaN	Galactosyltransferase family protein
Pp3c5_10760	AT1G14810	NaN	semialdehyde dehydrogenase family protein
Pp3c5_12940	AT4G35490	MRPL11	mitochondrial ribosomal protein L11
Pp3c5_14880	AT1G29940	NRPA2	nuclear RNA polymerase A2
Pp3c5_14900	AT2G17265	DMR1.HSK	homoserine kinase
Pp3c5_15220	AT1G60620	ATRPAC43.RPAC43	RNA polymerase I subunit 43
Pp3c5_15740	AT3G58140	NaN	phenylalanyl-tRNA synthetase class IIc family protein
Pp3c5_16070	AT2G40510	NaN	Ribosomal protein S26e family protein
Pp3c5_16770	AT5G08280	HEMC	hydroxymethylbilane synthase
Pp3c5_18660	AT4G29540	NaN	bacterial transferase hexapeptide repeat-containing protein
Pp3c5_20500	AT5G20920	EIF2 BETA.EMB1401	eukaryotic translation initiation factor 2 beta subunit
Pp3c5_23930	AT1G65440	GTB1	global transcription factor group B1
Pp3c5_24750	AT2G45440	DHDPS2	dihydrodipicolinate synthase
Pp3c5_25290	AT2G39550	ATGGT-IB.GGB.PGGT-I	Prenyltransferase family protein
Pp3c5_26400	AT2G29690	ASA2.ATHANSYNAB	anthranilate synthase 2
Pp3c5_26660	AT4G11820	EMB2778.FKP1.HMGS.MVA1	hydroxymethylglutaryl-CoA synthase / HMG-CoA synthase
Pp3c5_26860	AT1G70600	NaN	Ribosomal protein L18e/L15 superfamily protein
Pp3c5_26980	AT5G05730	AMT1.ASA1.JDL1.TRP5.WEI2	anthranilate synthase alpha subunit 1
Pp3c5_27000	AT1G61580	ARP2.RPL3B	R-protein L3 B
Pp3c5_27030	AT1G61580	ARP2.RPL3B	R-protein L3 B
Pp3c5_28210	AT3G13920	EIF4A1.RH4.TIF4A1	eukaryotic translation initiation factor 4A1
Pp3c6_610	AT4G31985	NaN	Ribosomal protein L39 family protein
Pp3c6_1080	AT3G13920	EIF4A1.RH4.TIF4A1	eukaryotic translation initiation factor 4A1
Pp3c6_1920	AT4G11160	NaN	Translation initiation factor 2. small GTP-binding protein
Pp3c6_2930	AT2G18950	ATHPT.HPT1.TPT1.VTE2	homogentisate phytyltransferase 1
Pp3c6_3460	AT5G40820	ATATR.ATR.ATRAD3	Ataxia telangiectasia-mutated and RAD3-related
Pp3c6_3470	AT1G70600	NaN	Ribosomal protein L18e/L15 superfamily protein
Pp3c6_3890	AT4G26500	ATSUFE.CPSUFE.EMB1374.SUFE1	chloroplast sulfur E
Pp3c6_3950	AT2G45440	DHDPS2	dihydrodipicolinate synthase
Pp3c6_7730	AT5G07090	NaN	Ribosomal protein S4 (RPS4A) family protein
Pp3c6_8910	AT2G40590	NaN	Ribosomal protein S26e family protein
Pp3c6_10280	AT2G21170	PDTPI.TIM	triosephosphate isomerase
Pp3c6_12700	AT3G54250	NaN	GHMP kinase family protein
Pp3c6_14770	AT1G67070	DIN9.PMI2	Mannose-6-phosphate isomerase. type I
Pp3c6_15170	AT1G78570	ATRHM1.RHM1.ROL1	rhamnose biosynthesis 1
Pp3c6_15300	AT1G63000	NRS/ER.UER1	nucleotide-rhamnose synthase/epimerase-reductase
Pp3c6_15310	AT1G63000	NRS/ER.UER1	nucleotide-rhamnose synthase/epimerase-reductase
Pp3c6_17160	AT5G01410	ATPDX1.ATPDX1.3.PDX1.PDX1.3.RSR4	Aldolase-type TIM barrel family protein
Pp3c6_17230	AT1G10840	TIF3H1	translation initiation factor 3 subunit H1
Pp3c6_17680	AT5G15200	NaN	Ribosomal protein S4
Pp3c6_19330	AT3G10950	NaN	Zinc-binding ribosomal protein family protein
Pp3c6_19470	AT2G39630	NaN	Nucleotide-diphospho-sugar transferases superfamily protein
Pp3c6_19930	AT2G33840	NaN	Tyrosyl-tRNA synthetase. class Ib. bacterial/mitochondrial
Pp3c6_21360	AT5G20040	ATIPT9.IPT9	isopentenyltransferase 9
Pp3c6_21770	AT1G34030	NaN	Ribosomal protein S13/S18 family
Pp3c6_21830	AT1G34030	NaN	Ribosomal protein S13/S18 family
Pp3c6_22940	AT2G46210	NaN	Fatty acid/sphingolipid desaturase
Pp3c6_28950	AT4G16450	NaN	NaN
Pp3c6_29520	AT4G00620	NaN	Amino acid dehydrogenase family protein
Pp3c7_370	AT5G25780	ATEIF3B-2.EIF3B.EIF3B-2	eukaryotic translation initiation factor 3B-2
Pp3c7_650	AT3G19820	CBB1.DIM.DIM1.DWF1.EVE1	cell elongation protein / DWARF1 / DIMINUTO (DIM)
Pp3c7_1450	AT2G29390	ATSMO2.SMO2-2	sterol 4-alpha-methyl-oxidase 2-2
Pp3c7_1650	AT4G02780	ABC33.ATCPS1.CPS.CPS1.GA1	Terpenoid cyclases/Protein prenyltransferases superfamily protein
Pp3c7_1880	AT4G02780	ABC33.ATCPS1.CPS.CPS1.GA1	Terpenoid cyclases/Protein prenyltransferases superfamily protein
Pp3c7_2620	AT5G35530	NaN	Ribosomal protein S3 family protein
Pp3c7_3770	AT3G13920	EIF4A1.RH4.TIF4A1	eukaryotic translation initiation factor 4A1
Pp3c7_4200	AT1G19580	GAMMA CA1	gamma carbonic anhydrase 1
Pp3c7_5560	AT2G39990	AteIF3f.EIF2.eIF3F	eukaryotic translation initiation factor 2
Pp3c7_5780	AT5G10360	EMB3010.RPS6B	Ribosomal protein S6e
Pp3c7_8060	AT3G07270	NaN	GTP cyclohydrolase I
Pp3c7_10260	AT1G62640	KAS III	3-ketoacyl-acyl carrier protein synthase III
Pp3c7_11840	AT4G00620	NaN	Amino acid dehydrogenase family protein
Pp3c7_12270	AT5G48760	NaN	Ribosomal protein L13 family protein
Pp3c7_13700	AT4G00585	NaN	NaN
Pp3c7_14200	AT5G02960	NaN	Ribosomal protein S12/S23 family protein
Pp3c7_15160	AT5G57850	NaN	D-aminoacid aminotransferase-like PLP-dependent enzymes superfamily protein
Pp3c7_17000	AT2G26560	PLA IIA.PLA2A.PLP2	phospholipase A 2A
Pp3c7_18950	AT3G19480	NaN	D-3-phosphoglycerate dehydrogenase
Pp3c7_20120	AT4G14320	NaN	Zinc-binding ribosomal protein family protein
Pp3c7_20160	AT2G42740	RPL16A	ribosomal protein large subunit 16A
Pp3c7_20680	AT2G47690	NaN	NADH-ubiquinone oxidoreductase-related
Pp3c7_21070	AT5G04800	NaN	Ribosomal S17 family protein
Pp3c7_21140	AT5G04800	NaN	Ribosomal S17 family protein
Pp3c7_21380	AT1G26880	NaN	Ribosomal protein L34e superfamily protein
Pp3c7_21930	AT1G69620	RPL34	ribosomal protein L34
Pp3c7_22030	AT3G05590	RPL18	ribosomal protein L18
Pp3c7_22280	AT5G19220	ADG2.APL1	ADP glucose pyrophosphorylase large subunit 1
Pp3c7_22400	AT1G26910	RPL10B	Ribosomal protein L16p/L10e family protein
Pp3c7_23800	AT5G07090	NaN	Ribosomal protein S4 (RPS4A) family protein
Pp3c7_24050	AT3G46100	ATHRS1.HRS1	Histidyl-tRNA synthetase 1
Pp3c7_25220	AT1G67430	NaN	Ribosomal protein L22p/L17e family protein
Pp3c7_25240	AT1G67430	NaN	Ribosomal protein L22p/L17e family protein
Pp3c7_25660	AT5G39740	OLI7.RPL5B	ribosomal protein L5 B
Pp3c7_26650	AT5G56670	NaN	Ribosomal protein S30 family protein
Pp3c7_26720	AT4G00100	ATRPS13A.PFL2.RPS13.RPS13A	ribosomal protein S13A
Pp3c8_1610	AT3G58660	NaN	Ribosomal protein L1p/L10e family
Pp3c8_2050	AT3G16950	LPD1.ptlpd1	lipoamide dehydrogenase 1

Pp3.3 gene*	Best hit At gene*	At_symbol*	At_description*
Pp3c8_3310	AT3G58680	ATMBF1B.MBF1B	multiprotein bridging factor 1B
Pp3c8_4960	AT5G49460	ACLB-2	ATP citrate lyase subunit B 2
Pp3c8_5640	AT3G57490	NaN	Ribosomal protein S5 family protein
Pp3c8_5770	AT1G70600	NaN	Ribosomal protein L18e/L15 superfamily protein
Pp3c8_6490	AT1G74910	NaN	ADP-glucose pyrophosphorylase family protein
Pp3c8_6500	AT1G74910	NaN	ADP-glucose pyrophosphorylase family protein
Pp3c8_6700	AT2G05320	NaN	beta-1,2-N-acetylglucosaminyltransferase II
Pp3c8_12080	AT1G04480	NaN	Ribosomal protein L14p/L23e family protein
Pp3c8_12410	AT3G27870	NaN	ATPase E1-E2 type family protein / haloacid dehalogenase-like hydrolase family protein
Pp3c8_13300	AT5G59230	NaN	transcription factor-related
Pp3c8_13880	AT2G34480	NaN	Ribosomal protein L18ae/LX family protein
Pp3c8_15480	AT1G11680	CYP51.CYP51A2.CYP51G1.EMB1738	CYTOCHROME P450 51G1
Pp3c8_16130	AT3G04400	emb2171	Ribosomal protein L14p/L23e family protein
Pp3c8_16210	AT4G28360	NaN	Ribosomal protein L22p/L17e family protein
Pp3c8_16590	AT4G22570	APT3	adenine phosphoribosyl transferase 3
Pp3c8_16820	AT2G37270	ATRPS5B.RPS5B	ribosomal protein 5B
Pp3c8_16860	AT2G37270	ATRPS5B.RPS5B	ribosomal protein 5B
Pp3c8_22360	AT4G16720	NaN	Ribosomal protein L23/L15e family protein
Pp3c8_22380	AT5G24510	NaN	60S acidic ribosomal protein family
Pp3c8_22480	AT1G48830	NaN	Ribosomal protein S7e family protein
Pp3c8_25050	AT2G20450	NaN	Ribosomal protein L14
Pp3c9_380	AT3G62870	NaN	Ribosomal protein L7Ae/L30e/S12e/Gadd45 family protein
Pp3c9_950	AT3G26618	ERF1-3	eukaryotic release factor 1-3
Pp3c9_1670	AT1G07210	NaN	Ribosomal protein S18
Pp3c9_2670	AT5G05170	ATCESA3.ATH-B.CESA3.CEV1.IXR1	Cellulose synthase family protein
Pp3c9_4143	AT2G13680	ATGSL02.CALS5.GLS2	callose synthase 5
Pp3c9_5060	AT5G51820	ATPGMP.PGM.PGM1.STF1	phosphoglucomutase
Pp3c9_5670	AT3G10050	OMR1	L-O-methylthreonine resistant 1
Pp3c9_7700	AT2G31170	SYCO ARATH	Cysteinyl-tRNA synthetase. class Ia family protein
Pp3c9_8500	AT1G52300	NaN	Zinc-binding ribosomal protein family protein
Pp3c9_12870	AT5G23300	PYRD	pyrimidine d
Pp3c9_12920	AT2G32260	ATCCT1.CCT1	phosphorylcholine cytidylyltransferase
Pp3c9_14000	AT5G53070	NaN	Ribosomal protein L9/RNase H1
Pp3c9_14720	AT3G16780	NaN	Ribosomal protein L19e family protein
Pp3c9_15130	AT5G14760	AO	L-aspartate oxidase
Pp3c9_15390	AT4G35630	PSAT	phosphoserine aminotransferase
Pp3c9_15430	AT3G28340	GATL10	galacturonosyltransferase-like 10
Pp3c9_15980	AT1G48270	GCR1	G-protein-coupled receptor 1
Pp3c9_18780	AT4G31985	NaN	Ribosomal protein L39 family protein
Pp3c9_19720	AT1G20050	HYD1	C-8,7 sterol isomerase
Pp3c9_21480	AT3G11510	NaN	Ribosomal protein S11 family protein
Pp3c9_26150	AT3G18410	NaN	Complex I subunit NDUFS6
Pp3c10_990	AT4G25740	NaN	RNA binding Plectin/S10 domain-containing protein
Pp3c10_2890	AT3G42050	NaN	vacuolar ATP synthase subunit H family protein
Pp3c10_4970	AT4G34110	ATPAB2.PAB2.PABP2	poly(A) binding protein 2
Pp3c10_6390	AT1G17890	GER2	NAD(P)-binding Rossmann-fold superfamily protein
Pp3c10_7370	AT3G49010	ATBBC1.BBC1.RSU2	breast basic conserved 1
Pp3c10_7980	AT3G43980	NaN	Ribosomal protein S14p/S29e family protein
Pp3c10_8090	AT2G19740	NaN	Ribosomal protein L31e family protein
Pp3c10_8200	AT5G53460	GLT1	NADH-dependent glutamate synthase 1
Pp3c10_8510	AT2G47760	ALG3.AtALG3	asparagine-linked glycosylation 3
Pp3c10_8730	AT5G40850	UPM1	urophorphyrin methylase 1
Pp3c10_9620	AT4G11120	NaN	translation elongation factor Ts (EF-Ts). putative
Pp3c10_9960	AT1G30000	MNS3	alpha-mannosidase 3
Pp3c10_11740	AT3G62830	ATUXS2.AUD1.UXS2	NAD(P)-binding Rossmann-fold superfamily protein
Pp3c10_12320	AT5G11900	NaN	Translation initiation factor SUI1 family protein
Pp3c10_15140	AT2G18110	NaN	Translation elongation factor EF1B/ribosomal protein S6 family protein
Pp3c10_19330	AT3G07160	ATGSL10.CALS9.gsl10	glucan synthase-like 10
Pp3c10_19590	AT1G70570	NaN	anthranilate phosphoribosyltransferase. putative
Pp3c10_20240	AT3G59540	NaN	Ribosomal L38e protein family
Pp3c10_20867	AT3G07160	ATGSL10.CALS9.gsl10	glucan synthase-like 10
Pp3c10_21170	AT4G25740	NaN	RNA binding Plectin/S10 domain-containing protein
Pp3c10_25460	AT1G61580	ARP2.RPL3B	R-protein L3 B
Pp3c11_520	AT5G04930	ALA1	aminophospholipid ATPase 1
Pp3c11_1000	AT4G26300	emb1027	Arginyl-tRNA synthetase. class Ic
Pp3c11_1100	AT4G00100	ATRPS13A.PFL2.RPS13.RPS13A	ribosomal protein S13A
Pp3c11_2940	AT1G26880	NaN	Ribosomal protein L34e superfamily protein
Pp3c11_3000	AT1G12350	ATCOAB.COAB	4-phospho-panto-thenoylcysteine synthetase
Pp3c11_3090	AT1G26880	NaN	Ribosomal protein L34e superfamily protein
Pp3c11_3690	AT2G17360	NaN	Ribosomal protein S4 (RPS4A) family protein
Pp3c11_3700	AT2G40010	NaN	Ribosomal protein L10 family protein
Pp3c11_4130	AT1G26910	RPL10B	Ribosomal protein L16p/L10e family protein
Pp3c11_4930	AT4G16710	NaN	glycosyltransferase family protein 28
Pp3c11_5720	AT5G19220	ADG2.APL1	ADP glucose pyrophosphorylase large subunit 1
Pp3c11_5840	AT1G67430	NaN	Ribosomal protein L22p/L17e family protein
Pp3c11_6420	AT5G39740	OLI7.RPL5B	ribosomal protein L5 B
Pp3c11_8230	AT3G20330	PYRB	PYRIMIDINE B
Pp3c11_8270	AT5G48300	ADG1.APS1	ADP glucose pyrophosphorylase 1
Pp3c11_9380	AT2G39290	PGP1.PGPS1.PGS1	phosphatidylglycerolphosphate synthase 1
Pp3c11_9630	AT1G74270	NaN	Ribosomal protein L35Ae family protein
Pp3c11_10490	AT5G59850	NaN	Ribosomal protein S8 family protein
Pp3c11_11070	AT5G02960	NaN	Ribosomal protein S12/S23 family protein
Pp3c11_12280	AT3G51440	NaN	Calcium-dependent phosphotriesterase superfamily protein
Pp3c11_17230	AT4G34670	NaN	Ribosomal protein S3Ae
Pp3c11_17260	AT4G34670	NaN	Ribosomal protein S3Ae
Pp3c11_17280	AT5G48760	NaN	Ribosomal protein L13 family protein
Pp3c11_17440	AT2G43360	BIO2.BIOB	Radical SAM superfamily protein
Pp3c11_18690	AT4G18330	NaN	Translation elongation factor EF1A/initiation factor IF2gamma family protein
Pp3c11_19160	AT2G35390	NaN	Phosphoribosyltransferase family protein
Pp3c11_19990	AT2G39260	NaN	binding;RNA binding
Pp3c11_21120	AT5G10360	EMB3010.RPS6B	Ribosomal protein S6e
Pp3c11_22520	AT1G47260	APFI.GAMMA CA2	gamma carbonic anhydrase 2
Pp3c11_22640	AT5G35530	NaN	Ribosomal protein S3 family protein
Pp3c11_22980	AT3G13920	EIF4A1.RH4.TIF4A1	eukaryotic translation initiation factor 4A1
Pp3c11_23800	AT1G64970	G-TMT.TMT1.VTE4	gamma-tocopherol methyltransferase
Pp3c11_25130	AT4G18100	NaN	Ribosomal protein L32e

Pp3.3 gene*	Best hit At gene*	At_symbol*	At_description*
Pp3c11_25960	AT5G25780	ATEIF3B-2.EIF3B.EIF3B-2	eukaryotic translation initiation factor 3B-2
Pp3c11_26120	AT3G19820	CBB1.DIM.DIM1.DWF1.EVE1	cell elongation protein / DWARF1 / DIMINUTO (DIM)
Pp3c12_320	AT5G52920	PKP-BETA1.PKP1.PKP2	plastidic pyruvate kinase beta subunit 1
Pp3c12_350	AT1G20330	CVP1.FRL1.SMT2	sterol methyltransferase 2
Pp3c12_3210	AT2G32060	NaN	Ribosomal protein L7Ae/L30e/S12e/Gadd45 family protein
Pp3c12_4480	AT4G24830	NaN	arginosuccinate synthase family
Pp3c12_5860	AT5G54160	ATOMT1.OMT1	O-methyltransferase 1
Pp3c12_7770	AT2G34480	NaN	Ribosomal protein L18ae/LX family protein
Pp3c12_8050	AT5G67510	NaN	Translation protein SH3-like family protein
Pp3c12_8200	AT3G54010	DEI1.PAS1	FKBP-type peptidyl-prolyl cis-trans isomerase family protein
Pp3c12_9050	AT3G62720	ATXT1.XT1.XXT1	xylosyltransferase 1
Pp3c12_9180	AT5G09500	NaN	Ribosomal protein S19 family protein
Pp3c12_9190	AT5G15200	NaN	Ribosomal protein S4
Pp3c12_9320	AT1G57860	NaN	Translation protein SH3-like family protein
Pp3c12_10200	AT4G36130	NaN	Ribosomal protein L2 family
Pp3c12_10760	AT5G28060	NaN	Ribosomal protein S24e family protein
Pp3c12_10870	AT5G28060	NaN	Ribosomal protein S24e family protein
Pp3c12_14310	AT4G04870	CLS	cardiolipin synthase
Pp3c12_14340	AT1G70570	NaN	anthranilate phosphoribosyltransferase. putative
Pp3c12_17640	AT4G36420	NaN	Ribosomal protein L12 family protein
Pp3c12_17990	AT3G59540	NaN	Ribosomal L38e protein family
Pp3c12_18260	AT5G59240	NaN	Ribosomal protein S8e family protein
Pp3c12_18499	AT5G26710	NaN	Glutamyl/glutaminyl-tRNA synthetase. class Ic
Pp3c12_18590	AT4G34670	NaN	Ribosomal protein S3Ae
Pp3c12_19360	AT3G55280	RPL23AB	ribosomal protein L23AB
Pp3c12_20050	AT5G51460	ATTPPA	Haloacid dehalogenase-like hydrolase (HAD) superfamily protein
Pp3c12_20520	AT3G11710	ATKRS-1	lysyl-tRNA synthetase 1
Pp3c12_20560	AT4G02120	NaN	CTP synthase family protein
Pp3c12_23740	AT4G22910	CCS52A1.FZR2	FIZZY-related 2
Pp3c12_25120	AT5G02960	NaN	Ribosomal protein S12/S23 family protein
Pp3c12_25380	AT1G15710	NaN	prephenate dehydrogenase family protein
Pp3c12_26130	AT4G20330	NaN	Transcription initiation factor TFIIE. beta subunit
Pp3c13_1280	AT5G57020	ATNMT1.NMT1	myristoyl-CoA:protein N-myristoyltransferase
Pp3c13_2360	AT1G57660	NaN	Translation protein SH3-like family protein
Pp3c13_3420	AT4G39080	VHA-A3	vacuolar proton ATPase A3
Pp3c13_4380	AT3G42640	AHA8.HA8	H(+)-ATPase 8
Pp3c13_5460	AT2G43710	FAB2.SSI2	Plant stearoyl-acyl-carrier-protein desaturase family protein
Pp3c13_8360	AT1G41880	NaN	Ribosomal protein L35Ae family protein
Pp3c13_8930	AT4G22930	DHOASE.PYR4	pyrimidin 4
Pp3c13_8990	AT5G46290	KAS I.KAS1	3-ketoacyl-acyl carrier protein synthase I
Pp3c13_10770	AT2G36620	RPL24A	ribosomal protein L24
Pp3c13_10890	AT3G56150	ATEIF3C-1.ATTIF3C1.EIF3C.EIF3C-1.TIF3C1	eukaryotic translation initiation factor 3C
Pp3c13_12320	AT2G34480	NaN	Ribosomal protein L18ae/LX family protein
Pp3c13_13363	AT2G04520	NaN	Nucleic acid-binding. OB-fold-like protein
Pp3c13_13620	AT2G04520	NaN	Nucleic acid-binding. OB-fold-like protein
Pp3c13_14220	AT1G12840	ATVHA-C.DET3	vacuolar ATP synthase subunit C (VATC)/ vacuolar proton pump C subunit (DET3)
Pp3c13_14790	AT1G65290	mtACP2	mitochondrial acyl carrier protein 2
Pp3c13_15330	AT2G20060	NaN	Ribosomal protein L4/L1 family
Pp3c13_15620	AT1G69410	ATELF5A-3.ELF5A-3	eukaryotic elongation factor 5A-3
Pp3c13_15900	AT3G49010	ATBBC1.BBC1.RSU2	breast basic conserved 1
Pp3c13_15920	AT3G55280	RPL23AB	ribosomal protein L23AB
Pp3c13_17460	AT5G04360	ATLDA.ATPU1.LDA.PU1	limit dextrinase
Pp3c13_19780	AT5G15200	NaN	Ribosomal protein S4
Pp3c13_20020	AT5G59240	NaN	Ribosomal protein S8e family protein
Pp3c13_20209	AT1G08130	ATLIG1.LIG1	DNA ligase 1
Pp3c13_20430	AT1G77130	GUX3.PGSIP2	plant glycogenin-like starch initiation protein 2
Pp3c13_21760	AT4G36130	NaN	Ribosomal protein L2 family
Pp3c13_22220	AT5G28060	NaN	Ribosomal protein S24e family protein
Pp3c13_22230	AT4G33680	AGD2	Pyridoxal phosphate (PLP)-dependent transferases superfamily protein
Pp3c13_23630	AT4G36130	NaN	Ribosomal protein L2 family
Pp3c13_24420	AT3G62870	NaN	Ribosomal protein L7Ae/L30e/S12e/Gadd45 family protein
Pp3c13_24450	AT3G62870	NaN	Ribosomal protein L7Ae/L30e/S12e/Gadd45 family protein
Pp3c13_24470	AT1G67730	ATKCR1.KCR1.YBR159	beta-ketoacyl reductase 1
Pp3c14_1810	AT4G25740	NaN	RNA binding Plectin/S10 domain-containing protein
Pp3c14_2730	AT3G57660	NRPA1	nuclear RNA polymerase A1
Pp3c14_3490	AT3G49010	ATBBC1.BBC1.RSU2	breast basic conserved 1
Pp3c14_4100	AT1G74060	NaN	Ribosomal protein L6 family protein
Pp3c14_4160	AT1G74060	NaN	Ribosomal protein L6 family protein
Pp3c14_7180	AT3G45030	NaN	Ribosomal protein S10p/S20e family protein
Pp3c14_7550	AT2G19740	NaN	Ribosomal protein L31e family protein
Pp3c14_8480	AT5G10160	NaN	Thioesterase superfamily protein
Pp3c14_8600	AT1G33140	PGY2	Ribosomal protein L6 family
Pp3c14_8740	AT5G53460	GLT1	NADH-dependent glutamate synthase 1
Pp3c14_9600	AT3G48930	EMB1080	Nucleic acid-binding. OB-fold-like protein
Pp3c14_10580	AT4G18100	NaN	Ribosomal protein L32e
Pp3c14_11550	AT1G30620	HSR8.MUR4.UXE1	NAD(P)-binding Rossmann-fold superfamily protein
Pp3c14_11870	AT2G37040	ATPAL1.PAL1	PHE ammonia lyase 1
Pp3c14_12850	AT5G19220	ADG2.APL1	ADP glucose pyrophosphorylase large subunit 1
Pp3c14_13580	AT4G39200	NaN	Ribosomal protein S25 family protein
Pp3c14_15630	AT3G62830	ATUXS2.AUD1.UXS2	NAD(P)-binding Rossmann-fold superfamily protein
Pp3c14_17850	AT5G42810	ATIPK1.IPK1	inositol-pentakisphosphate 2-kinase 1
Pp3c14_19610	AT5G03850	NaN	Nucleic acid-binding. OB-fold-like protein
Pp3c14_19640	AT3G02080	NaN	Ribosomal protein S19e family protein
Pp3c14_20220	AT5G57870	eIFiso4G1	MIF4G domain-containing protein / MA3 domain-containing protein
Pp3c14_20670	AT2G26100	NaN	Galactosyltransferase family protein
Pp3c14_20870	AT3G47930	ATGLDH.GLDH	L-galactono-1.4-lactone dehydrogenase
Pp3c14_21510	AT5G24510	NaN	60S acidic ribosomal protein family
Pp3c14_21790	AT5G08170	ATAIH.EMB1873	porphyromonas-type peptidyl-arginine deiminase family protein
Pp3c14_22050	AT4G34570	THY-2	thymidylate synthase 2
Pp3c14_22080	AT5G58770	NaN	Undecaprenyl pyrophosphate synthetase family protein
Pp3c14_22450	AT5G50375	CPI1	cyclopropyl isomerase
Pp3c14_22920	AT4G16720	NaN	Ribosomal protein L23/L15e family protein
Pp3c14_24710	AT3G05020	ACP.ACP1	acyl carrier protein 1
Pp3c14_25550	AT3G11170	FAD7.FADD	fatty acid desaturase 7
Pp3c14_26100	AT3G03050	ATCSLD3.CSLD3.KJK	cellulose synthase-like D3
Pp3c14_26580	AT5G55500	ATXYLT.XYLT	beta-1,2-xylosyltransferase

Pp3.3 gene*	Best hit At gene*	At_symbol*	At_description*
Pp3c15_290	AT3G62870	NaN	Ribosomal protein L7Ae/L30e/S12e/Gadd45 family protein
Pp3c15_4020	AT2G31170	SYCO ARATH	Cysteinyl-tRNA synthetase. class Ia family protein
Pp3c15_4110	AT5G04490	VTE5	vitamin E pathway gene 5
Pp3c15_4370	AT1G15250	NaN	Zinc-binding ribosomal protein family protein
Pp3c15_5130	AT3G16780	NaN	Ribosomal protein L19e family protein
Pp3c15_5610	AT2G46210	NaN	Fatty acid/sphingolipid desaturase
Pp3c15_5860	AT4G27090	NaN	Ribosomal protein L14
Pp3c15_7380	AT2G44040	NaN	Dihydrodipicolinate reductase. bacterial/plant
Pp3c15_10960	AT4G17910	NaN	transferases. transferring acyl groups
Pp3c15_11360	AT2G05990	ENR1.MOD1	NAD(P)-binding Rossmann-fold superfamily protein
Pp3c15_12700	AT2G16485	NaN	nucleic acid binding;zinc ion binding;DNA binding
Pp3c15_13540	AT4G24440	NaN	transcription initiation factor IIA gamma chain / TFIIA-gamma (TFIIA-S)
Pp3c15_14410	AT3G53430	NaN	Ribosomal protein L11 family protein
Pp3c15_14860	AT4G31985	NaN	Ribosomal protein L39 family protein
Pp3c15_21130	AT1G50480	THFS	10-formyltetrahydrofolate synthetase
Pp3c15_21360	AT5G35530	NaN	Ribosomal protein S3 family protein
Pp3c15_21510	AT3G45030	NaN	Ribosomal protein S10p/S20e family protein
Pp3c15_22240	AT3G11510	NaN	Ribosomal protein S11 family protein
Pp3c15_23330	AT4G30950	FAD6.FADC.SFD4	fatty acid desaturase 6
Pp3c15_23380	AT1G75670	NaN	DNA-directed RNA polymerases
Pp3c15_23980	AT3G57610	ADSS	adenylosuccinate synthase
Pp3c15_25430	AT5G10360	EMB3010.RPS6B	Ribosomal protein S6e
Pp3c15_25780	AT5G10360	EMB3010.RPS6B	Ribosomal protein S6e
Pp3c15_25810	AT4G31700	RPS6.RPS6A	ribosomal protein S6
Pp3c16_410	AT3G16780	NaN	Ribosomal protein L19e family protein
Pp3c16_620	AT1G66520	pde194	formyltransferase. putative
Pp3c16_2090	AT1G34030	NaN	Ribosomal protein S13/S18 family
Pp3c16_3760	AT5G35530	NaN	Ribosomal protein S3 family protein
Pp3c16_5320	AT3G54110	ATPUMP1.ATUCP1.PUMP1.UCP.UCP1	plant uncoupling mitochondrial protein 1
Pp3c16_5340	AT3G55360	ATTSC13.CER10.ECR.TSC13	3-oxo-5-alpha-steroid 4-dehydrogenase family protein
Pp3c16_5490	AT1G67070	DIN9.PMI2	Mannose-6-phosphate isomerase. type I
Pp3c16_7970	AT1G78570	ATRHM1.RHM1.ROL1	rhamnose biosynthesis 1
Pp3c16_7980	AT2G32060	NaN	Ribosomal protein L7Ae/L30e/S12e/Gadd45 family protein
Pp3c16_9210	AT2G40510	NaN	Ribosomal protein S26e family protein
Pp3c16_9360	AT2G36990	ATSIG6.SIG6.SIGF.SOLDAT8	RNApolymerase sigma-subunit F
Pp3c16_10400	AT1G77940	NaN	Ribosomal protein L7Ae/L30e/S12e/Gadd45 family protein
Pp3c16_10880	AT3G45100	SETH2	UDP-Glycosyltransferase superfamily protein
Pp3c16_11330	AT2G21170	PDTPI.TIM	triosephosphate isomerase
Pp3c16_11740	AT5G48930	HCT	hydroxycinnamoyl-CoA shikimate/quinate hydroxycinnamoyl transferase
Pp3c16_11800	AT5G01410	ATPDX1.ATPDX1.3.PDX1.PDX1.3.RSR4	Aldolase-type TIM barrel family protein
Pp3c16_12160	AT5G09500	NaN	Ribosomal protein S19 family protein
Pp3c16_13360	AT2G36390	BE3.SBE2.1	starch branching enzyme 2.1
Pp3c16_13640	AT3G04400	emb2171	Ribosomal protein L14p/L23e family protein
Pp3c16_15220	AT4G00100	ATRPS13A.PFL2.RPS13.RPS13A	ribosomal protein S13A
Pp3c16_16670	AT3G25660	NaN	Amidase family protein
Pp3c16_18880	AT5G58420	NaN	Ribosomal protein S4 (RPS4A) family protein
Pp3c16_20070	AT4G10450	NaN	Ribosomal protein L6 family
Pp3c16_21800	AT1G63160	RFC2	replication factor C 2
Pp3c16_22430	AT1G70600	NaN	Ribosomal protein L18e/L15 superfamily protein
Pp3c17_1680	AT1G20330	CVP1.FRL1.SMT2	sterol methyltransferase 2
Pp3c17_2160	AT5G39850	NaN	Ribosomal protein S4
Pp3c17_2240	AT3G61130	GAUT1.LGT1	galacturonosyltransferase 1
Pp3c17_3630	AT2G32060	NaN	Ribosomal protein L7Ae/L30e/S12e/Gadd45 family protein
Pp3c17_4670	AT4G39200	NaN	Ribosomal protein S25 family protein
Pp3c17_5630	AT1G23820	SPDS1	spermidine synthase 1
Pp3c17_5990	AT5G49030	OVA2	tRNA synthetase class I (I. L. M and V) family protein
Pp3c17_6430	AT4G13050	NaN	Acyl-ACP thioesterase
Pp3c17_12020	AT3G26618	ERF1-3	eukaryotic release factor 1-3
Pp3c17_13270	AT1G05170	NaN	Galactosyltransferase family protein
Pp3c17_13300	AT5G03850	NaN	Nucleic acid-binding. OB-fold-like protein
Pp3c17_13460	AT4G23660	AtPPT1.PPT1	polyprenyltransferase 1
Pp3c17_13600	AT4G29910	ATORC5.ORC5	origin recognition complex protein 5
Pp3c17_13610	AT1G34130	STT3B	staurosporin and temperature sensitive 3-like b
Pp3c17_13784	AT1G65070	NaN	DNA mismatch repair protein MutS. type 2
Pp3c17_15200	AT3G02080	NaN	Ribosomal protein S19e family protein
Pp3c17_15920	AT5G28840	GME	GDP-D-mannose 3',5'-epimerase
Pp3c17_16180	AT1G67320	NaN	DNA primase. large subunit family
Pp3c17_16690	AT5G17990	pat1.TRP1	tryptophan biosynthesis 1
Pp3c17_18440	AT5G57870	eIFiso4G1	MIF4G domain-containing protein / MA3 domain-containing protein
Pp3c17_18640	AT5G57870	eIFiso4G1	MIF4G domain-containing protein / MA3 domain-containing protein
Pp3c17_18740	AT5G57870	eIFiso4G1	MIF4G domain-containing protein / MA3 domain-containing protein
Pp3c17_20510	AT4G14710	ATARD2	RmlC-like cupins superfamily protein
Pp3c17_22180	AT4G16720	NaN	Ribosomal protein L23/L15e family protein
Pp3c17_22190	AT5G24510	NaN	60S acidic ribosomal protein family
Pp3c17_22380	AT3G03050	ATCSLD3.CSLD3.KJK	cellulose synthase-like D3
Pp3c17_23500	AT1G13560	AAPT1.ATAAPT1	aminoalcoholphosphotransferase 1
Pp3c17_24020	AT5G57590	BIO1	adenosylmethionine-8-amino-7-oxononanoate transaminases
Pp3c17_24100	AT4G25050	ACP4	acyl carrier protein 4
Pp3c17_24230	AT4G25050	ACP4	acyl carrier protein 4
Pp3c18_1350	AT5G18380	NaN	Ribosomal protein S5 domain 2-like superfamily protein
Pp3c18_2090	AT4G34670	NaN	Ribosomal protein S3Ae
Pp3c18_4460	AT3G63170	NaN	Chalcone-flavanone isomerase family protein
Pp3c18_5450	AT4G38510	NaN	ATPase. V1 complex. subunit B protein
Pp3c18_6190	AT1G79900	ATMBAC2.BAC2	Mitochondrial substrate carrier family protein
Pp3c18_8580	AT2G33040	ATP3	gamma subunit of Mt ATP synthase
Pp3c18_13130	AT4G27070	TSB2	tryptophan synthase beta-subunit 2
Pp3c18_13160	AT1G15250	NaN	Zinc-binding ribosomal protein family protein
Pp3c18_13220	AT5G10920	NaN	L-Aspartase-like family protein
Pp3c18_13860	AT2G43460	NaN	Ribosomal L38e protein family
Pp3c18_14440	AT3G16780	NaN	Ribosomal protein L19e family protein
Pp3c18_14700	AT4G36810	GGPS1	geranylgeranyl pyrophosphate synthase 1
Pp3c18_14720	AT4G33250	ATTIF3K1.EIF3K.TIF3K1	eukaryotic translation initiation factor 3K
Pp3c18_14740	AT2G37560	ATORC2.ORC2	origin recognition complex second largest subunit 2
Pp3c18_15140	AT4G24670	TAR2	tryptophan aminotransferase related 2
Pp3c18_20060	AT1G09640	NaN	Translation elongation factor EF1B. gamma chain
Pp3c18_20140	AT5G60540	ATPDX2.EMB2407.PDX2	pyridoxine biosynthesis 2

Pp3.3 gene*	Best hit At gene*	At_symbol*	At_description*
Pp3c18_20150	AT2G07050	CAS1	cycloartenol synthase 1
Pp3c18_20850	AT1G32900	NaN	UDP-Glycosyltransferase superfamily protein
Pp3c18_21210	AT5G17920	ATCIMS.ATMETS.ATMS1	Cobalamin-independent synthase family protein
Pp3c19_3790	AT1G24360	NaN	NAD(P)-binding Rossmann-fold superfamily protein
Pp3c19_4120	AT5G63840	PSL5.RSW3	Glycosyl hydrolases family 31 protein
Pp3c19_5720	AT3G13490	ATKRS-2.OVA5	Lysyl-tRNA synthetase. class II
Pp3c19_6490	AT5G59240	NaN	Ribosomal protein S8e family protein
Pp3c19_7230	AT4G25890	NaN	60S acidic ribosomal protein family
Pp3c19_7430	AT2G38040	CAC3	acetyl Co-enzyme a carboxylase carboxyltransferase alpha subunit
Pp3c19_7712	AT3G01180	AtSS2.SS2	starch synthase 2
Pp3c19_8030	AT2G37250	ADK.ATPADK1	adenosine kinase
Pp3c19_8410	AT1G27680	APL2	ADPGLC-PPase large subunit
Pp3c19_9420	AT5G10330	ATHPA1.EMB2196.HISN6A.HPA1	histidinol phosphate aminotransferase 1
Pp3c19_10310	AT3G04840	NaN	Ribosomal protein S3Ae
Pp3c19_10330	AT5G48760	NaN	Ribosomal protein L13 family protein
Pp3c19_11630	AT2G33040	ATP3	gamma subunit of Mt ATP synthase
Pp3c19_11880	AT1G31860	AT-IE.HISN2	histidine biosynthesis bifunctional protein (HISIE)
Pp3c19_13120	AT1G72550	NaN	tRNA synthetase beta subunit family protein
Pp3c19_17550	AT1G09640	NaN	Translation elongation factor EF1B. gamma chain
Pp3c19_18340	AT4G14070	AAE15	acyl-activating enzyme 15
Pp3c19_19270	AT5G37510	CI76.EMB1467	NADH-ubiquinone dehydrogenase. mitochondrial. putative
Pp3c19_21200	AT1G70190	NaN	Ribosomal protein L7/L12. C-terminal/adaptor protein ClpS-like
Pp3c19_22360	AT3G58610	NaN	ketol-acid reductoisomerase
Pp3c19_22410	AT5G18380	NaN	Ribosomal protein S5 domain 2-like superfamily protein
Pp3c20_1360	AT1G48830	NaN	Ribosomal protein S7e family protein
Pp3c20_1550	AT3G01120	ATCYS1.CGS.CGS1.MTO1	Pyridoxal phosphate (PLP)-dependent transferases superfamily protein
Pp3c20_1830	AT1G16740	NaN	Ribosomal protein L20
Pp3c20_2460	AT2G44530	NaN	Phosphoribosyltransferase family protein
Pp3c20_2550	AT1G52300	NaN	Zinc-binding ribosomal protein family protein
Pp3c20_2940	AT5G53340	NaN	Galactosyltransferase family protein
Pp3c20_3610	AT3G25570	NaN	Adenosylmethionine decarboxylase family protein
Pp3c20_5100	AT4G32770	ATSDX1.VTE1	tocopherol cyclase. chloroplast/ vitamin E deficient 1 (VTE1)/ sucrose export defective 1 (SXD1)
Pp3c20_5690	AT3G57290	ATEIF3E-1.ATINT6.EIF3E.INT-6.INT6.TIF3E1	eukaryotic translation initiation factor 3E
Pp3c20_7040	AT4G29840	MTO2.TS	Pyridoxal-5'-phosphate-dependent enzyme family protein
Pp3c20_7570	AT1G65290	mtACP2	mitochondrial acyl carrier protein 2
Pp3c20_10450	AT5G45400	ATRPA70C.RPA70C	Replication factor-A protein 1-related
Pp3c20_12680	AT2G42710	NaN	Ribosomal protein L1p/L10e family
Pp3c20_15150	AT2G34480	NaN	Ribosomal protein L18ae/LX family protein
Pp3c20_17090	AT2G45300	NaN	RNA 3'â'-terminal phosphate cyclase/enolpyruvate transferase. alpha/beta
Pp3c20_17250	AT1G80190	PSF1	partner of SLD five 1
Pp3c20_18740	AT3G57490	NaN	Ribosomal protein S5 family protein
Pp3c20_18830	AT1G70600	NaN	Ribosomal protein L18e/L15 superfamily protein
Pp3c20_18840	AT3G28715	NaN	ATPase. V0/A0 complex. subunit C/D
Pp3c20_18970	AT2G37270	ATRPS5B.RPS5B	ribosomal protein 5B
Pp3c20_19020	AT5G26830	NaN	Threonyl-tRNA synthetase
Pp3c20_19160	AT2G30200	NaN	catalytics;transferases;[acyl-carrier-protein] S-malonyltransferases;binding
Pp3c20_21880	AT1G79460	ATKS.ATKS1.GA2.KS.KS1	Terpenoid cyclases/Protein prenyltransferases superfamily protein
Pp3c20_22440	AT3G48560	AHAS.ALS.CSR1.IMR1.TZP5	chlorsulfuron/imidazolinone resistant 1
Pp3c21_580	AT5G52520	OVA6.PRORS1	Class II aaRS and biotin synthetases superfamily protein
Pp3c21_3730	AT5G17920	ATCIMS.ATMETS.ATMS1	Cobalamin-independent synthase family protein
Pp3c21_5280	AT4G01210	NaN	glycosyl transferase family 1 protein
Pp3c21_5470	AT4G27070	TSB2	tryptophan synthase beta-subunit 2
Pp3c21_5560	AT4G27070	TSB2	tryptophan synthase beta-subunit 2
Pp3c21_6620	AT5G59240	NaN	Ribosomal protein S8e family protein
Pp3c21_8340	AT3G02870	VTC4	Inositol monophosphatase family protein
Pp3c21_8640	AT2G40550	ETG1	E2F target gene 1
Pp3c21_8670	AT1G48520	GATB	GLU-ADT subunit B
Pp3c21_8680	AT2G24090	NaN	Ribosomal protein L35
Pp3c21_9750	AT5G07990	CYP75B1.D501.TT7	Cytochrome P450 superfamily protein
Pp3c21_9920	AT5G52100	crr1	Dihydrodipicolinate reductase. bacterial/plant
Pp3c21_9940	AT3G53180	NaN	glutamate-ammonia ligases;catalytics;glutamate-ammonia ligases
Pp3c21_10860	AT5G51690	ACS12	1-amino-cyclopropane-1-carboxylate synthase 12
Pp3c21_10940	AT1G56350	NaN	Peptide chain release factor 2
Pp3c21_11230	AT3G16780	NaN	Ribosomal protein L19e family protein
Pp3c21_11700	AT2G33040	ATP3	gamma subunit of Mt ATP synthase
Pp3c21_14550	AT1G09640	NaN	Translation elongation factor EF1B. gamma chain
Pp3c21_16160	AT4G25890	NaN	60S acidic ribosomal protein family
Pp3c21_18320	AT1G20260	NaN	ATPase. V1 complex. subunit B protein
Pp3c21_20630	AT5G18380	NaN	Ribosomal protein S5 domain 2-like superfamily protein
Pp3c21_20900	AT3G58610	NaN	ketol-acid reductoisomerase
Pp3c21_21510	AT4G34670	NaN	Ribosomal protein S3Ae
Pp3c21_21540	AT3G61110	ARS27A.RS27A	ribosomal protein S27
Pp3c22_90	AT3G61130	GAUT1.LGT1	galacturonosyltransferase 1
Pp3c22_160	AT1G58080	ATATP-PRT1.ATP-PRT1.HISN1A	ATP phosphoribosyl transferase 1
Pp3c22_470	AT1G26810	GALT1	galactosyltransferase1
Pp3c22_490	AT5G27450	MK.MVK	mevalonate kinase
Pp3c22_1860	AT5G59240	NaN	Ribosomal protein S8e family protein
Pp3c22_1870	AT5G12290	DGS1	dgd1 suppressor 1
Pp3c22_2050	AT5G13280	AK.AK-LYS1.AK1	aspartate kinase 1
Pp3c22_2590	AT3G04240	SEC	Tetratricopeptide repeat (TPR)-like superfamily protein
Pp3c22_3160	AT5G48930	HCT	hydroxycinnamoyl-CoA shikimate/quinate hydroxycinnamoyl transferase
Pp3c22_4140	AT5G48930	HCT	hydroxycinnamoyl-CoA shikimate/quinate hydroxycinnamoyl transferase
Pp3c22_7200	AT5G08415	NaN	Radical SAM superfamily protein
Pp3c22_7830	AT1G09830	NaN	Glycinamide ribonucleotide (GAR) synthetase
Pp3c22_8510	AT4G27070	TSB2	tryptophan synthase beta-subunit 2
Pp3c22_9260	AT1G80560	ATIMD2.IMD2	isopropylmalate dehydrogenase 2
Pp3c22_11160	AT1G08510	FATB	fatty acyl-ACP thioesterases B
Pp3c22_12850	AT4G01850	AtSAM2.MAT2.SAM-2.SAM2	S-adenosylmethionine synthetase 2
Pp3c22_13130	AT4G00620	NaN	Amino acid dehydrogenase family protein
Pp3c22_13970	AT5G50210	OLD5.QS.SUFE3	quinolinate synthase
Pp3c22_15010	AT1G09640	NaN	Translation elongation factor EF1B. gamma chain
Pp3c22_15210	AT2G43750	ACS1.ATCS-B.CPACS1.OASB	O-acetylserine (thiol) lyase B
Pp3c22_15340	AT1G72550	NaN	tRNA synthetase beta subunit family protein
Pp3c22_18300	AT4G34670	NaN	Ribosomal protein S3Ae
Pp3c22_18860	AT1G12910	ATAN11.LWD1	Transducin/WD40 repeat-like superfamily protein
Pp3c22_21090	AT5G18380	NaN	Ribosomal protein S5 domain 2-like superfamily protein

Pp3.3 gene*	Best hit At gene*	At_symbol*	At_description*
Pp3c22_22010	AT1G66580	RPL10C.SAG24	senescence associated gene 24
Pp3c22_23080	AT1G08980	AMI1.ATAMI1.ATTOC64-I.TOC64-I	amidase 1
Pp3c23_390	AT5G44635	MCM6	minichromosome maintenance (MCM2/3/5) family protein
Pp3c23_3680	AT1G51590	MANIB.MNS1	alpha-mannosidase 1
Pp3c23_4120	AT1G07320	RPL4	ribosomal protein L4
Pp3c23_4520	AT3G04400	emb2171	Ribosomal protein L14p/L23e family protein
Pp3c23_6150	AT2G37270	ATRPS5B.RPS5B	ribosomal protein 5B
Pp3c23_6600	AT5G54770	THI1.THI4.TZ	thiazole biosynthetic enzyme. chloroplast (ARA6) (THI1) (THI4)
Pp3c23_8220	AT1G23290	RPL27A.RPL27AB	Ribosomal protein L18e/L15 superfamily protein
Pp3c23_8410	AT3G57490	NaN	Ribosomal protein S5 family protein
Pp3c23_9440	AT3G01040	GAUT13	galacturonosyltransferase 13
Pp3c23_9570	AT3G63250	ATHMT-2.HMT-2.HMT2	homocysteine methyltransferase 2
Pp3c23_9830	AT3G28340	GATL10	galacturonosyltransferase-like 10
Pp3c23_10040	AT5G27700	NaN	Ribosomal protein S21e
Pp3c23_10130	AT1G64060	ATRBOH F.ATRBOHF.RBOH F.RBOHAP108.RBOHF	respiratory burst oxidase protein F
Pp3c23_11670	AT2G45790	ATPMM.PMM	phosphomannomutase
Pp3c23_12730	AT1G08360	NaN	Ribosomal protein L1p/L10e family
Pp3c23_15390	AT5G26710	NaN	Glutamyl/glutaminyl-tRNA synthetase. class Ic
Pp3c23_16600	AT3G14390	NaN	Pyridoxal-dependent decarboxylase family protein
Pp3c23_17260	AT1G52300	NaN	Zinc-binding ribosomal protein family protein
Pp3c23_17430	AT3G55620	emb1624	Translation initiation factor IF6
Pp3c23_18100	AT5G24510	NaN	60S acidic ribosomal protein family
Pp3c23_18210	AT3G17465	RPL3P	ribosomal protein L3 plastid
Pp3c23_18280	AT1G48830	NaN	Ribosomal protein S7e family protein
Pp3c23_18410	AT3G01120	ATCYS1.CGS.CGS1.MTO1	Pyridoxal phosphate (PLP)-dependent transferases superfamily protein
Pp3c23_22060	AT2G20450	NaN	Ribosomal protein L14
Pp3c24_20	AT5G14660	ATDEF2.DEF2.PDF1B	peptide deformylase 1B
Pp3c24_2340	AT4G25740	NaN	RNA binding Plectin/S10 domain-containing protein
Pp3c24_3690	AT1G08360	NaN	Ribosomal protein L1p/L10e family
Pp3c24_3950	AT3G01040	GAUT13	galacturonosyltransferase 13
Pp3c24_5500	AT3G57490	NaN	Ribosomal protein S5 family protein
Pp3c24_5780	AT3G57490	NaN	Ribosomal protein S5 family protein
Pp3c24_5980	AT2G23420	NAPRT2	nicotinate phosphoribosyltransferase 2
Pp3c24_6930	AT5G66680	DGL1	dolichyl-diphosphooligosaccharide-protein glycosyltransferase 48kDa subunit family protein
Pp3c24_7340	AT3G14390	NaN	Pyridoxal-dependent decarboxylase family protein
Pp3c24_7341	AT3G14390	NaN	Pyridoxal-dependent decarboxylase family protein
Pp3c24_8160	AT5G27700	NaN	Ribosomal protein S21e
Pp3c24_9920	AT5G10160	NaN	Thioesterase superfamily protein
Pp3c24_10460	AT1G50430	7RED.DWF5.LE.PA.ST7R	Ergosterol biosynthesis ERG4/ERG24 family
Pp3c24_12530	AT4G11630	NaN	Ribosomal protein L19 family protein
Pp3c24_13910	AT3G55620	emb1624	Translation initiation factor IF6
Pp3c24_15540	AT4G23620	NaN	Ribosomal protein L25/Gln-tRNA synthetase. anti-codon-binding domain
Pp3c24_16040	AT4G26850	VTC2	mannose-1-phosphate guanylyltransferase (GDP)s
Pp3c24_16390	AT3G20540	POLGAMMA1	polymerase gamma 1
Pp3c24_18220	AT5G16130	NaN	Ribosomal protein S7e family protein
Pp3c24_18560	AT1G65290	mtACP2	mitochondrial acyl carrier protein 2
Pp3c24_18680	AT3G01800	NaN	Ribosome recycling factor
Pp3c24_18700	AT4G17190	FPS2	farnesyl diphosphate synthase 2
Pp3c24_18790	AT2G34480	NaN	Ribosomal protein L18ae/LX family protein
Pp3c24_18820	AT1G63680	ATMURE.MURE.PDE316	acid-amino acid ligases;ligases;ATP binding;ATP binding;ligases
Pp3c24_18880	AT2G37270	ATRPS5B.RPS5B	ribosomal protein 5B
Pp3c24_20890	AT4G27090	NaN	Ribosomal protein L14
Pp3c25_230	AT5G18790	NaN	Ribosomal protein L33 family protein
Pp3c25_300	AT2G31490	NaN	NaN
Pp3c25_1050	AT1G10170	ATNFXL1.NFXL1	NF-X-like 1
Pp3c25_2440	AT1G10840	TIF3H1	translation initiation factor 3 subunit H1
Pp3c25_2670	AT1G10840	TIF3H1	translation initiation factor 3 subunit H1
Pp3c25_3730	AT5G58420	NaN	Ribosomal protein S4 (RPS4A) family protein
Pp3c25_3736	AT5G58420	NaN	Ribosomal protein S4 (RPS4A) family protein
Pp3c25_5720	AT2G29630	PY.THIC	thiaminC
Pp3c25_5840	AT4G19710	AK-HSDH.AK-HSDH II	aspartate kinase-homoserine dehydrogenase ii
Pp3c25_5870	AT4G19710	AK-HSDH.AK-HSDH II	aspartate kinase-homoserine dehydrogenase ii
Pp3c25_8110	AT4G08350	GTA02.GTA2	global transcription factor group A2
Pp3c25_9320	AT1G77940	NaN	Ribosomal protein L7Ae/L30e/S12e/Gadd45 family protein
Pp3c25_11980	AT1G26880	NaN	Ribosomal protein L34e superfamily protein
Pp3c25_13260	AT1G70600	NaN	Ribosomal protein L18e/L15 superfamily protein
Pp3c26_330	AT3G62870	NaN	Ribosomal protein L7Ae/L30e/S12e/Gadd45 family protein
Pp3c26_350	AT2G47610	NaN	Ribosomal protein L7Ae/L30e/S12e/Gadd45 family protein
Pp3c26_360	AT3G62870	NaN	Ribosomal protein L7Ae/L30e/S12e/Gadd45 family protein
Pp3c26_2200	AT5G55140	NaN	ribosomal protein L30 family protein
Pp3c26_3000	AT5G27700	NaN	Ribosomal protein S21e
Pp3c26_6700	AT4G18820	NaN	AAA-type ATPase family protein
Pp3c26_7220	AT5G17920	ATCIMS.ATMETS.ATMS1	Cobalamin-independent synthase family protein
Pp3c26_10900	AT5G18110	NCBP	novel cap-binding protein
Pp3c26_11220	AT4G14320	NaN	Zinc-binding ribosomal protein family protein
Pp3c26_11290	AT4G11420	ATEIF3A-1.ATTIF3A1.EIF3A.EIF3A-1.TIF3A1	eukaryotic translation initiation factor 3A
Pp3c26_11300	AT1G30010	NaN	Intron maturase. type II family protein
Pp3c26_13710	AT1G17410	NaN	Nucleoside diphosphate kinase family protein
Pp3c26_13720	AT5G02610	NaN	Ribosomal L29 family protein
Pp3c26_14180	AT2G20370	KAM1.MUR3	Exostosin family protein
Pp3c26_14310	AT2G24490	ATRPA2.ATRPA32A.ROR1.RPA2.RPA32A	replicon protein A2
Pp3c26_15390	AT3G10950	NaN	Zinc-binding ribosomal protein family protein
Pp3c27_190	AT5G20920	EIF2 BETA.EMB1401	eukaryotic translation initiation factor 2 beta subunit
Pp3c27_1320	AT5G35530	NaN	Ribosomal protein S3 family protein
Pp3c27_1920	AT5G15200	NaN	Ribosomal protein S4
Pp3c27_1950	AT3G25660	NaN	Amidase family protein
Pp3c27_1990	AT1G04950	ATTAF6.TAF6.TAFII59	TATA BOX ASSOCIATED FACTOR II 59
Pp3c27_2260	AT5G45775	NaN	Ribosomal L5P family protein
Pp3c27_2550	AT3G54110	ATPUMP1.ATUCP1.PUMP1.UCP.UCP1	plant uncoupling mitochondrial protein 1
Pp3c27_2710	AT2G44050	COS1	6,7-dimethyl-8-ribityllumazine synthase/ DMRL synthase/ lumazine synthase/ riboflavin synthase
Pp3c27_2780	AT3G02080	NaN	Ribosomal protein S19e family protein
Pp3c27_2790	AT1G34360	NaN	translation initiation factor 3 (IF-3) family protein
Pp3c27_3130	AT5G48930	HCT	hydroxycinnamoyl-CoA shikimate/quinate hydroxycinnamoyl transferase
Pp3c27_3230	AT5G48930	HCT	hydroxycinnamoyl-CoA shikimate/quinate hydroxycinnamoyl transferase
Pp3c27_4050	AT5G15530	BCCP2.CAC1-B	biotin carboxyl carrier protein 2
Pp3c27_4150	AT3G48730	GSA2	glutamate-1-semialdehyde 2,1-aminomutase 2

Pp3.3 gene*	Best hit At gene*	At_symbol*	At_description*
Pp3c27_4310	AT4G30000	NaN	Dihydropterin pyrophosphokinase / Dihydropteroate synthase
Pp3c27_7320	AT1G50200	ACD.ALATS	Alanyl-tRNA synthetase
Pp3c27_7820	AT5G09500	NaN	Ribosomal protein S19 family protein
Pp3c27_8190	AT1G33140	PGY2	Ribosomal protein L6 family
Pp3s93_30	AT3G13920	EIF4A1.RH4.TIF4A1	eukaryotic translation initiation factor 4A1
Pp3s116_30	AT3G48930	EMB1080	Nucleic acid-binding. OB-fold-like protein
Pp3s127_20	AT5G19220	ADG2.APL1	ADP glucose pyrophosphorylase large subunit 1

*Sixty-nine of 774 genes are colored in red as the common DEGs both 1cell-DGE and 5'DGE.

Supplementary Table S11. DEGs in 24 h-high identified using 1cell-DGE categorized by the GO term "translation".

Pp3.3 gene*	Best hit At gene*	At_symbol*	At_description*
Pp3c1_420	AT5G56670	NaN	Ribosomal protein S30 family protein
Pp3c1_1530	AT3G10950	NaN	Zinc-binding ribosomal protein family protein
Pp3c1_1800	AT1G26880	NaN	Ribosomal protein L34e superfamily protein
Pp3c1_3170	AT1G26910	RPL10B	Ribosomal protein L16p/L10e family protein
Pp3c1_3480	AT3G05590	RPL18	ribosomal protein L18
Pp3c1_4030	AT1G41880	NaN	Ribosomal protein L35Ae family protein
Pp3c1_4290	AT5G59850	NaN	Ribosomal protein S8 family protein
Pp3c1_5320	AT3G44890	RPL9	ribosomal protein L9
Pp3c1_5330	AT3G05590	RPL18	ribosomal protein L18
Pp3c1_6160	AT4G00100	ATRPS13A.PFL2.RPS13.RPS13A	ribosomal protein S13A
Pp3c1_6650	AT1G67430	NaN	Ribosomal protein L22p/L17e family protein
Pp3c1_7160	AT5G39740	OLI7.RPL5B	ribosomal protein L5 B
Pp3c1_7830	AT5G56940	NaN	Ribosomal protein S16 family protein
Pp3c1_8280	AT2G40010	NaN	Ribosomal protein L10 family protein
Pp3c1_8290	AT5G58420	NaN	Ribosomal protein S4 (RPS4A) family protein
Pp3c1_10010	AT5G64050	ATERS.ERS.OVA3	glutamate tRNA synthetase
Pp3c1_15830	AT5G48760	NaN	Ribosomal protein L13 family protein
Pp3c1_16170	AT2G36620	RPL24A	ribosomal protein L24
Pp3c1_18050	AT2G44860	NaN	Ribosomal protein L24e family protein
Pp3c1_18400	AT4G25740	NaN	RNA binding Plectin/S10 domain-containing protein
Pp3c1_19680	AT1G25350	OVA9	glutamine-tRNA ligase. putative / glutaminyl-tRNA synthetase. putative / GlnRS. putative
Pp3c1_21860	AT3G45030	NaN	Ribosomal protein S10p/S20e family protein
Pp3c1_22820	AT5G27820	NaN	Ribosomal L18p/L5e family protein
Pp3c1_24120	AT1G72370	AP40.P40.RP40.RPSAA	40s ribosomal protein SA
Pp3c1_24300	AT1G74050	NaN	Ribosomal protein L6 family protein
Pp3c1_26280	AT3G49010	ATBBC1.BBC1.RSU2	breast basic conserved 1
Pp3c1_28770	AT3G10090	NaN	Nucleic acid-binding. OB-fold-like protein
Pp3c1_29390	AT4G26310	NaN	elongation factor P (EF-P) family protein
Pp3c1_32410	AT1G26740	NaN	Ribosomal L32p protein family
Pp3c1_32490	AT4G18100	NaN	Ribosomal protein L32e
Pp3c1_34390	AT2G19740	NaN	Ribosomal protein L31e family protein
Pp3c1_36220	AT5G24510	NaN	60S acidic ribosomal protein family
Pp3c1_37420	AT4G16720	NaN	Ribosomal protein L23/L15e family protein
Pp3c1_40490	AT5G46160	NaN	Ribosomal protein L14p/L23e family protein
Pp3c2_3850	AT3G62120	NaN	Class II aaRS and biotin synthetases superfamily protein
Pp3c2_5780	AT4G16720	NaN	Ribosomal protein L23/L15e family protein
Pp3c2_6730	AT3G44010	NaN	Ribosomal protein S14p/S29e family protein
Pp3c2_9590	AT4G18100	NaN	Ribosomal protein L32e
Pp3c2_9650	AT3G53740	NaN	Ribosomal protein L36e family protein
Pp3c2_9830	AT1G14610	TWN2.VALRS	valyl-tRNA synthetase / valine--tRNA ligase (VALRS)
Pp3c2_10060	AT3G02080	NaN	Ribosomal protein S19e family protein
Pp3c2_11670	AT5G23535	NaN	KOW domain-containing protein
Pp3c2_13300	AT1G74060	NaN	Ribosomal protein L6 family protein
Pp3c2_13690	AT3G48930	EMB1080	Nucleic acid-binding. OB-fold-like protein
Pp3c2_14800	AT4G39200	NaN	Ribosomal protein S25 family protein
Pp3c2_16490	AT2G19740	NaN	Ribosomal protein L31e family protein
Pp3c2_16770	AT4G39280	NaN	phenylalanyl-tRNA synthetase. putative / phenylalanine--tRNA ligase. putative
Pp3c2_18620	AT4G25740	NaN	RNA binding Plectin/S10 domain-containing protein
Pp3c2_24130	AT5G02960	NaN	Ribosomal protein S12/S23 family protein
Pp3c2_24930	AT2G17360	NaN	Ribosomal protein S4 (RPS4A) family protein
Pp3c2_27110	AT1G15390	ATDEF1.PDF1A	peptide deformylase 1A
Pp3c2_28870	AT5G56670	NaN	Ribosomal protein S30 family protein
Pp3c2_29600	AT1G26910	RPL10B	Ribosomal protein L16p/L10e family protein
Pp3c2_29840	AT1G67430	NaN	Ribosomal protein L22p/L17e family protein
Pp3c2_31540	AT5G39740	OLI7.RPL5B	ribosomal protein L5 B
Pp3c2_32050	AT5G59850	NaN	Ribosomal protein S8 family protein
Pp3c2_32310	AT4G18040	AT.EIF4E1.CUM1.EIF4E.eIF4E1	eukaryotic translation initiation factor 4E
Pp3c2_32510	AT3G05590	RPL18	ribosomal protein L18
Pp3c2_33280	AT4G18730	RPL16B	ribosomal protein L16B
Pp3c2_35700	AT4G31460	NaN	Ribosomal L28 family
Pp3c2_38140	AT5G56940	NaN	Ribosomal protein S16 family protein
Pp3c3_1140	AT1G07770	RPS15A	ribosomal protein S15A
Pp3c3_1620	AT3G10950	NaN	Zinc-binding ribosomal protein family protein
Pp3c3_2800	AT5G02610	NaN	Ribosomal L29 family protein
Pp3c3_3090	AT3G22300	RPS10	ribosomal protein S10
Pp3c3_3480	AT1G74270	NaN	Ribosomal protein L35Ae family protein
Pp3c3_5550	AT1G57660	NaN	Translation protein SH3-like family protein
Pp3c3_5690	AT4G30930	NFD1	Ribosomal protein L21
Pp3c3_8500	AT3G56150	ATEIF3C-1.ATTIF3C1.EIF3C.EIF3C-1.TIF3C1	eukaryotic translation initiation factor 3C
Pp3c3_8930	AT3G53020	RPL24.RPL24B.STV1	Ribosomal protein L24e family protein
Pp3c3_10260	AT4G14320	NaN	Zinc-binding ribosomal protein family protein
Pp3c3_10840	AT2G36620	RPL24A	ribosomal protein L24
Pp3c3_11250	AT2G34480	NaN	Ribosomal protein L18ae/LX family protein
Pp3c3_12210	AT2G18020	EMB2296	Ribosomal protein L2 family
Pp3c3_14880	AT5G04800	NaN	Ribosomal S17 family protein
Pp3c3_19700	AT5G28060	NaN	Ribosomal protein S24e family protein
Pp3c3_21130	AT3G49010	ATBBC1.BBC1.RSU2	breast basic conserved 1
Pp3c3_23540	AT3G58700	NaN	Ribosomal L5P family protein
Pp3c3_23720	AT5G15200	NaN	Ribosomal protein S4
Pp3c3_25300	AT2G18110	NaN	Translation elongation factor EF1B/ribosomal protein S6 family protein
Pp3c3_25640	AT2G43460	NaN	Ribosomal L38e protein family
Pp3c3_26860	AT4G36420	NaN	Ribosomal protein L12 family protein
Pp3c3_35760	AT5G47320	RPS19	ribosomal protein S19
Pp3c3_36280	AT1G53880	NaN	Eukaryotic translation initiation factor 2B (eIF-2B) family protein
Pp3c4_3220	AT5G27700	NaN	Ribosomal protein S21e

Pp3.3 gene*	Best hit At gene*	At_symbol*	At_description*
Pp3c4_6350	AT3G49080	NaN	Ribosomal protein S5 domain 2-like superfamily protein
Pp3c4_7510	AT3G11710	ATKRS-1	lysyl-tRNA synthetase 1
Pp3c4_8620	AT3G55280	RPL23AB	ribosomal protein L23AB
Pp3c4_11370	AT5G59240	NaN	Ribosomal protein S8e family protein
Pp3c4_11930	AT2G36170	NaN	Ubiquitin supergroup;Ribosomal protein L40e
Pp3c4_12690	AT4G36130	NaN	Ribosomal protein L2 family
Pp3c4_13370	AT3G58700	NaN	Ribosomal L5P family protein
Pp3c4_13380	AT3G11710	ATKRS-1	lysyl-tRNA synthetase 1
Pp3c4_14160	AT4G34670	NaN	Ribosomal protein S3Ae
Pp3c4_15290	AT5G15200	NaN	Ribosomal protein S4
Pp3c4_15480	AT1G77940	NaN	Ribosomal protein L7Ae/L30e/S12e/Gadd45 family protein
Pp3c4_21520	AT1G57860	NaN	Translation protein SH3-like family protein
Pp3c4_24414	AT3G56150	ATEIF3C-1.ATTIF3C1.EIF3C.EIF3C-1.TIF3C1	eukaryotic translation initiation factor 3C
Pp3c4_26450	AT4G11420	ATEIF3A-1.ATTIF3A1.EIF3A.EIF3A-1.TIF3A1	eukaryotic translation initiation factor 3A
Pp3c4_27540	AT5G59850	NaN	Ribosomal protein S8 family protein
Pp3c4_29560	AT3G13882	NaN	Ribosomal protein L34
Pp3c4_29970	AT1G74270	NaN	Ribosomal protein L35Ae family protein
Pp3c5_2800	AT1G34030	NaN	Ribosomal protein S13/S18 family
Pp3c5_3870	AT3G58700	NaN	Ribosomal L5P family protein
Pp3c5_4330	AT1G77940	NaN	Ribosomal protein L7Ae/L30e/S12e/Gadd45 family protein
Pp3c5_5330	AT5G35530	NaN	Ribosomal protein S3 family protein
Pp3c5_5750	AT1G07770	RPS15A	ribosomal protein S15A
Pp3c5_6000	AT5G03850	NaN	Nucleic acid-binding. OB-fold-like protein
Pp3c5_6600	AT2G13540	ABH1.ATCBP80.CBP80.ENS	ARM repeat superfamily protein
Pp3c5_9890	AT2G33840	NaN	Tyrosyl-tRNA synthetase. class Ib. bacterial/mitochondrial
Pp3c5_10680	AT3G20230	NaN	Ribosomal L18p/L5e family protein
Pp3c5_12940	AT4G35490	MRPL11	mitochondrial ribosomal protein L11
Pp3c5_15740	AT3G58140	NaN	phenylalanyl-tRNA synthetase class IIc family protein
Pp3c5_16070	AT2G40510	NaN	Ribosomal protein S26e family protein
Pp3c5_20500	AT5G20920	EIF2 BETA.EMB1401	eukaryotic translation initiation factor 2 beta subunit
Pp3c5_26860	AT1G70600	NaN	Ribosomal protein L18e/L15 superfamily protein
Pp3c5_27000	AT1G61580	ARP2.RPL3B	R-protein L3 B
Pp3c5_27030	AT1G61580	ARP2.RPL3B	R-protein L3 B
Pp3c5_28210	AT3G13920	EIF4A1.RH4.TIF4A1	eukaryotic translation initiation factor 4A1
Pp3c6_610	AT4G31985	NaN	Ribosomal protein L39 family protein
Pp3c6_1080	AT3G13920	EIF4A1.RH4.TIF4A1	eukaryotic translation initiation factor 4A1
Pp3c6_1920	AT4G11160	NaN	Translation initiation factor 2. small GTP-binding protein
Pp3c6_3470	AT1G70600	NaN	Ribosomal protein L18e/L15 superfamily protein
Pp3c6_7730	AT5G07090	NaN	Ribosomal protein S4 (RPS4A) family protein
Pp3c6_8910	AT2G40590	NaN	Ribosomal protein S26e family protein
Pp3c6_17230	AT1G10840	TIF3H1	translation initiation factor 3 subunit H1
Pp3c6_17680	AT5G15200	NaN	Ribosomal protein S4
Pp3c6_19330	AT3G10950	NaN	Zinc-binding ribosomal protein family protein
Pp3c6_19930	AT2G33840	NaN	Tyrosyl-tRNA synthetase. class Ib. bacterial/mitochondrial
Pp3c6_21770	AT1G34030	NaN	Ribosomal protein S13/S18 family
Pp3c6_21830	AT1G34030	NaN	Ribosomal protein S13/S18 family
Pp3c7_370	AT5G25780	ATEIF3B-2.EIF3B.EIF3B-2	eukaryotic translation initiation factor 3B-2
Pp3c7_2620	AT5G35530	NaN	Ribosomal protein S3 family protein
Pp3c7_3770	AT3G13920	EIF4A1.RH4.TIF4A1	eukaryotic translation initiation factor 4A1
Pp3c7_5560	AT2G39990	AteIF3f.EIF2.eIF3F	eukaryotic translation initiation factor 2
Pp3c7_5780	AT5G10360	EMB3010.RPS6B	Ribosomal protein S6e
Pp3c7_12270	AT5G48760	NaN	Ribosomal protein L13 family protein
Pp3c7_14200	AT5G02960	NaN	Ribosomal protein S12/S23 family protein
Pp3c7_20120	AT4G14320	NaN	Zinc-binding ribosomal protein family protein
Pp3c7_20160	AT2G42740	RPL16A	ribosomal protein large subunit 16A
Pp3c7_21070	AT5G04800	NaN	Ribosomal S17 family protein
Pp3c7_21140	AT5G04800	NaN	Ribosomal S17 family protein
Pp3c7_21380	AT1G26880	NaN	Ribosomal protein L34e superfamily protein
Pp3c7_21930	AT1G69620	RPL34	ribosomal protein L34
Pp3c7_22030	AT3G05590	RPL18	ribosomal protein L18
Pp3c7_22400	AT1G26910	RPL10B	Ribosomal protein L16p/L10e family protein
Pp3c7_23800	AT5G07090	NaN	Ribosomal protein S4 (RPS4A) family protein
Pp3c7_24050	AT3G46100	ATHRS1.HRS1	Histidyl-tRNA synthetase 1
Pp3c7_25220	AT1G67430	NaN	Ribosomal protein L22p/L17e family protein
Pp3c7_25240	AT1G67430	NaN	Ribosomal protein L22p/L17e family protein
Pp3c7_25660	AT5G39740	OLI7.RPL5B	ribosomal protein L5 B
Pp3c7_26650	AT5G56670	NaN	Ribosomal protein S30 family protein
Pp3c7_26720	AT4G00100	ATRPS13A.PFL2.RPS13.RPS13A	ribosomal protein S13A
Pp3c8_1610	AT3G58660	NaN	Ribosomal protein L1p/L10e family
Pp3c8_5640	AT3G57490	NaN	Ribosomal protein S5 family protein
Pp3c8_5770	AT1G70600	NaN	Ribosomal protein L18e/L15 superfamily protein
Pp3c8_12080	AT1G04480	NaN	Ribosomal protein L14p/L23e family protein
Pp3c8_13880	AT2G34480	NaN	Ribosomal protein L18ae/LX family protein
Pp3c8_16130	AT3G04400	emb2171	Ribosomal protein L14p/L23e family protein
Pp3c8_16210	AT4G28360	NaN	Ribosomal protein L22p/L17e family protein
Pp3c8_16820	AT2G37270	ATRPS5B.RPS5B	ribosomal protein 5B
Pp3c8_16860	AT2G37270	ATRPS5B.RPS5B	ribosomal protein 5B
Pp3c8_22360	AT4G16720	NaN	Ribosomal protein L23/L15e family protein
Pp3c8_22380	AT5G24510	NaN	60S acidic ribosomal protein family
Pp3c8_22480	AT1G48830	NaN	Ribosomal protein S7e family protein
Pp3c8_25050	AT2G20450	NaN	Ribosomal protein L14
Pp3c9_380	AT3G62870	NaN	Ribosomal protein L7Ae/L30e/S12e/Gadd45 family protein
Pp3c9_950	AT3G26618	ERF1-3	eukaryotic release factor 1-3
Pp3c9_1670	AT1G07210	NaN	Ribosomal protein S18
Pp3c9_7700	AT2G31170	SYCO ARATH	Cysteinyl-tRNA synthetase. class Ia family protein
Pp3c9_8500	AT1G52300	NaN	Zinc-binding ribosomal protein family protein

Pp3.3 gene*	Best hit At gene*	At_symbol*	At_description*
Pp3c9_14000	AT5G53070	NaN	Ribosomal protein L9/RNase H1
Pp3c9_14720	AT3G16780	NaN	Ribosomal protein L19e family protein
Pp3c9_18780	AT4G31985	NaN	Ribosomal protein L39 family protein
Pp3c9_21480	AT3G11510	NaN	Ribosomal protein S11 family protein
Pp3c10_990	AT4G25740	NaN	RNA binding Plectin/S10 domain-containing protein
Pp3c10_4970	AT4G34110	ATPAB2.PAB2.PABP2	poly(A) binding protein 2
Pp3c10_7370	AT3G49010	ATBBC1.BBC1.RSU2	breast basic conserved 1
Pp3c10_7980	AT3G43980	NaN	Ribosomal protein S14p/S29e family protein
Pp3c10_8090	AT2G19740	NaN	Ribosomal protein L31e family protein
Pp3c10_9620	AT4G11120	NaN	translation elongation factor Ts (EF-Ts). putative
Pp3c10_12320	AT5G11900	NaN	Translation initiation factor SUI1 family protein
Pp3c10_15140	AT2G18110	NaN	Translation elongation factor EF1B/ribosomal protein S6 family protein
Pp3c10_20240	AT3G59540	NaN	Ribosomal L38e protein family
Pp3c10_21170	AT4G25740	NaN	RNA binding Plectin/S10 domain-containing protein
Pp3c10_25460	AT1G61580	ARP2.RPL3B	R-protein L3 B
Pp3c11_1000	AT4G26300	emb1027	Arginyl-tRNA synthetase. class Ic
Pp3c11_1100	AT4G00100	ATRPS13A.PFL2.RPS13.RPS13A	ribosomal protein S13A
Pp3c11_2940	AT1G26880	NaN	Ribosomal protein L34e superfamily protein
Pp3c11_3090	AT1G26880	NaN	Ribosomal protein L34e superfamily protein
Pp3c11_3690	AT2G17360	NaN	Ribosomal protein S4 (RPS4A) family protein
Pp3c11_3700	AT2G40010	NaN	Ribosomal protein L10 family protein
Pp3c11_4130	AT1G26910	RPL10B	Ribosomal protein L16p/L10e family protein
Pp3c11_5840	AT1G67430	NaN	Ribosomal protein L22p/L17e family protein
Pp3c11_6420	AT5G39740	OLI7.RPL5B	ribosomal protein L5 B
Pp3c11_9630	AT1G74270	NaN	Ribosomal protein L35Ae family protein
Pp3c11_10490	AT5G59850	NaN	Ribosomal protein S8 family protein
Pp3c11_11070	AT5G02960	NaN	Ribosomal protein S12/S23 family protein
Pp3c11_17230	AT4G34670	NaN	Ribosomal protein S3Ae
Pp3c11_17260	AT4G34670	NaN	Ribosomal protein S3Ae
Pp3c11_17280	AT5G48760	NaN	Ribosomal protein L13 family protein
Pp3c11_18690	AT4G18330	NaN	Translation elongation factor EF1A/initiation factor IF2gamma family protein
Pp3c11_19990	AT2G39260	NaN	binding;RNA binding
Pp3c11_21120	AT5G10360	EMB3010.RPS6B	Ribosomal protein S6e
Pp3c11_22640	AT5G35530	NaN	Ribosomal protein S3 family protein
Pp3c11_22980	AT3G13920	EIF4A1.RH4.TIF4A1	eukaryotic translation initiation factor 4A1
Pp3c11_25130	AT4G18100	NaN	Ribosomal protein L32e
Pp3c11_25960	AT5G25780	ATEIF3B-2.EIF3B.EIF3B-2	eukaryotic translation initiation factor 3B-2
Pp3c12_3210	AT2G32060	NaN	Ribosomal protein L7Ae/L30e/S12e/Gadd45 family protein
Pp3c12_7770	AT2G34480	NaN	Ribosomal protein L18ae/LX family protein
Pp3c12_8050	AT5G67510	NaN	Translation protein SH3-like family protein
Pp3c12_9180	AT5G09500	NaN	Ribosomal protein S19 family protein
Pp3c12_9190	AT5G15200	NaN	Ribosomal protein S4
Pp3c12_9320	AT1G57860	NaN	Translation protein SH3-like family protein
Pp3c12_10200	AT4G36130	NaN	Ribosomal protein L2 family
Pp3c12_10760	AT5G28060	NaN	Ribosomal protein S24e family protein
Pp3c12_10870	AT5G28060	NaN	Ribosomal protein S24e family protein
Pp3c12_17640	AT4G36420	NaN	Ribosomal protein L12 family protein
Pp3c12_17990	AT3G59540	NaN	Ribosomal L38e protein family
Pp3c12_18260	AT5G59240	NaN	Ribosomal protein S8e family protein
Pp3c12_18499	AT5G26710	NaN	Glutamyl/glutaminyl-tRNA synthetase. class Ic
Pp3c12_18590	AT4G34670	NaN	Ribosomal protein S3Ae
Pp3c12_19360	AT3G55280	RPL23AB	ribosomal protein L23AB
Pp3c12_20520	AT3G11710	ATKRS-1	lysyl-tRNA synthetase 1
Pp3c12_25120	AT5G02960	NaN	Ribosomal protein S12/S23 family protein
Pp3c13_2360	AT1G57660	NaN	Translation protein SH3-like family protein
Pp3c13_8360	AT1G41880	NaN	Ribosomal protein L35Ae family protein
Pp3c13_10770	AT2G36620	RPL24A	ribosomal protein L24
Pp3c13_10890	AT3G56150	ATEIF3C-1.ATTIF3C1.EIF3C.EIF3C-1.TIF3C1	eukaryotic translation initiation factor 3C
Pp3c13_12320	AT2G34480	NaN	Ribosomal protein L18ae/LX family protein
Pp3c13_13363	AT2G04520	NaN	Nucleic acid-binding. OB-fold-like protein
Pp3c13_13620	AT2G04520	NaN	Nucleic acid-binding. OB-fold-like protein
Pp3c13_15330	AT2G20060	NaN	Ribosomal protein L4/L1 family
Pp3c13_15620	AT1G69410	ATELF5A-3.ELF5A-3	eukaryotic elongation factor 5A-3
Pp3c13_15900	AT3G49010	ATBBC1.BBC1.RSU2	breast basic conserved 1
Pp3c13_15920	AT3G55280	RPL23AB	ribosomal protein L23AB
Pp3c13_19780	AT5G15200	NaN	Ribosomal protein S4
Pp3c13_20020	AT5G59240	NaN	Ribosomal protein S8e family protein
Pp3c13_21760	AT4G36130	NaN	Ribosomal protein L2 family
Pp3c13_22220	AT5G28060	NaN	Ribosomal protein S24e family protein
Pp3c13_23630	AT4G36130	NaN	Ribosomal protein L2 family
Pp3c13_24420	AT3G62870	NaN	Ribosomal protein L7Ae/L30e/S12e/Gadd45 family protein
Pp3c13_24450	AT3G62870	NaN	Ribosomal protein L7Ae/L30e/S12e/Gadd45 family protein
Pp3c14_1810	AT4G25740	NaN	RNA binding Plectin/S10 domain-containing protein
Pp3c14_3490	AT3G49010	ATBBC1.BBC1.RSU2	breast basic conserved 1
Pp3c14_4100	AT1G74060	NaN	Ribosomal protein L6 family protein
Pp3c14_4160	AT1G74060	NaN	Ribosomal protein L6 family protein
Pp3c14_7180	AT3G45030	NaN	Ribosomal protein S10p/S20e family protein
Pp3c14_7550	AT2G19740	NaN	Ribosomal protein L31e family protein
Pp3c14_8600	AT1G33140	PGY2	Ribosomal protein L6 family
Pp3c14_9600	AT3G48930	EMB1080	Nucleic acid-binding. OB-fold-like protein
Pp3c14_10580	AT4G18100	NaN	Ribosomal protein L32e
Pp3c14_13580	AT4G39200	NaN	Ribosomal protein S25 family protein
Pp3c14_19610	AT5G03850	NaN	Nucleic acid-binding. OB-fold-like protein
Pp3c14_19640	AT3G02080	NaN	Ribosomal protein S19e family protein
Pp3c14_20220	AT5G57870	eIFiso4G1	MIF4G domain-containing protein / MA3 domain-containing protein
Pp3c14_21510	AT5G24510	NaN	60S acidic ribosomal protein family

Pp3.3 gene*	Best hit At gene*	At_symbol*	At_description*
Pp3c14_22920	AT4G16720	NaN	Ribosomal protein L23/L15e family protein
Pp3c15_290	AT3G62870	NaN	Ribosomal protein L7Ae/L30e/S12e/Gadd45 family protein
Pp3c15_4020	AT2G31170	SYCO ARATH	Cysteinyl-tRNA synthetase. class Ia family protein
Pp3c15_4370	AT1G15250	NaN	Zinc-binding ribosomal protein family protein
Pp3c15_5130	AT3G16780	NaN	Ribosomal protein L19e family protein
Pp3c15_5860	AT4G27090	NaN	Ribosomal protein L14
Pp3c15_14410	AT3G53430	NaN	Ribosomal protein L11 family protein
Pp3c15_14860	AT4G31985	NaN	Ribosomal protein L39 family protein
Pp3c15_21360	AT5G35530	NaN	Ribosomal protein S3 family protein
Pp3c15_21510	AT3G45030	NaN	Ribosomal protein S10p/S20e family protein
Pp3c15_22240	AT3G11510	NaN	Ribosomal protein S11 family protein
Pp3c15_25430	AT5G10360	EMB3010.RPS6B	Ribosomal protein S6e
Pp3c15_25780	AT5G10360	EMB3010.RPS6B	Ribosomal protein S6e
Pp3c15_25810	AT4G31700	RPS6.RPS6A	ribosomal protein S6
Pp3c16_410	AT3G16780	NaN	Ribosomal protein L19e family protein
Pp3c16_2090	AT1G34030	NaN	Ribosomal protein S13/S18 family
Pp3c16_3760	AT5G35530	NaN	Ribosomal protein S3 family protein
Pp3c16_7980	AT2G32060	NaN	Ribosomal protein L7Ae/L30e/S12e/Gadd45 family protein
Pp3c16_9210	AT2G40510	NaN	Ribosomal protein S26e family protein
Pp3c16_10400	AT1G77940	NaN	Ribosomal protein L7Ae/L30e/S12e/Gadd45 family protein
Pp3c16_12160	AT5G09500	NaN	Ribosomal protein S19 family protein
Pp3c16_13640	AT3G04400	emb2171	Ribosomal protein L14p/L23e family protein
Pp3c16_15220	AT4G00100	ATRPS13A.PFL2.RPS13.RPS13A	ribosomal protein S13A
Pp3c16_16670	AT3G25660	NaN	Amidase family protein
Pp3c16_18880	AT5G58420	NaN	Ribosomal protein S4 (RPS4A) family protein
Pp3c16_20070	AT4G10450	NaN	Ribosomal protein L6 family
Pp3c16_22430	AT1G70600	NaN	Ribosomal protein L18e/L15 superfamily protein
Pp3c17_2160	AT5G39850	NaN	Ribosomal protein S4
Pp3c17_3630	AT2G32060	NaN	Ribosomal protein L7Ae/L30e/S12e/Gadd45 family protein
Pp3c17_4670	AT4G39200	NaN	Ribosomal protein S25 family protein
Pp3c17_5990	AT5G49030	OVA2	tRNA synthetase class I (I. L. M and V) family protein
Pp3c17_12020	AT3G26618	ERF1-3	eukaryotic release factor 1-3
Pp3c17_13300	AT5G03850	NaN	Nucleic acid-binding. OB-fold-like protein
Pp3c17_15200	AT3G02080	NaN	Ribosomal protein S19e family protein
Pp3c17_18440	AT5G57870	eIFiso4G1	MIF4G domain-containing protein / MA3 domain-containing protein
Pp3c17_18640	AT5G57870	eIFiso4G1	MIF4G domain-containing protein / MA3 domain-containing protein
Pp3c17_18740	AT5G57870	eIFiso4G1	MIF4G domain-containing protein / MA3 domain-containing protein
Pp3c17_22180	AT4G16720	NaN	Ribosomal protein L23/L15e family protein
Pp3c17_22190	AT5G24510	NaN	60S acidic ribosomal protein family
Pp3c18_1350	AT5G18380	NaN	Ribosomal protein S5 domain 2-like superfamily protein
Pp3c18_2090	AT4G34670	NaN	Ribosomal protein S3Ae
Pp3c18_13160	AT1G15250	NaN	Zinc-binding ribosomal protein family protein
Pp3c18_13860	AT2G43460	NaN	Ribosomal L38e protein family
Pp3c18_14440	AT3G16780	NaN	Ribosomal protein L19e family protein
Pp3c18_14720	AT4G33250	ATTIF3K1.EIF3K.TIF3K1	eukaryotic translation initiation factor 3K
Pp3c18_20060	AT1G09640	NaN	Translation elongation factor EF1B. gamma chain
Pp3c19_5720	AT3G13490	ATKRS-2.OVA5	Lysyl-tRNA synthetase. class II
Pp3c19_6490	AT5G59240	NaN	Ribosomal protein S8e family protein
Pp3c19_7230	AT4G25890	NaN	60S acidic ribosomal protein family
Pp3c19_10310	AT3G04840	NaN	Ribosomal protein S3Ae
Pp3c19_10330	AT5G48760	NaN	Ribosomal protein L13 family protein
Pp3c19_13120	AT1G72550	NaN	tRNA synthetase beta subunit family protein
Pp3c19_17550	AT1G09640	NaN	Translation elongation factor EF1B. gamma chain
Pp3c19_21200	AT1G70190	NaN	Ribosomal protein L7/L12. C-terminal/adaptor protein ClpS-like
Pp3c19_22410	AT5G18380	NaN	Ribosomal protein S5 domain 2-like superfamily protein
Pp3c20_1360	AT1G48830	NaN	Ribosomal protein S7e family protein
Pp3c20_1830	AT1G16740	NaN	Ribosomal protein L20
Pp3c20_2550	AT1G52300	NaN	Zinc-binding ribosomal protein family protein
Pp3c20_5690	AT3G57290	ATEIF3E-1.ATINT6.EIF3E.INT-6.INT6.TIF3E1	eukaryotic translation initiation factor 3E
Pp3c20_12680	AT2G42710	NaN	Ribosomal protein L1p/L10e family
Pp3c20_15150	AT2G34480	NaN	Ribosomal protein L18ae/LX family protein
Pp3c20_18740	AT3G57490	NaN	Ribosomal protein S5 family protein
Pp3c20_18830	AT1G70600	NaN	Ribosomal protein L18e/L15 superfamily protein
Pp3c20_18970	AT2G37270	ATRPS5B.RPS5B	ribosomal protein 5B
Pp3c20_19020	AT5G26830	NaN	Threonyl-tRNA synthetase
Pp3c21_580	AT5G52520	OVA6.PRORS1	Class II aaRS and biotin synthetases superfamily protein
Pp3c21_6620	AT5G59240	NaN	Ribosomal protein S8e family protein
Pp3c21_8670	AT1G48520	GATB	GLU-ADT subunit B
Pp3c21_8680	AT2G24090	NaN	Ribosomal protein L35
Pp3c21_10940	AT1G56350	NaN	Peptide chain release factor 2
Pp3c21_11230	AT3G16780	NaN	Ribosomal protein L19e family protein
Pp3c21_14550	AT1G09640	NaN	Translation elongation factor EF1B. gamma chain
Pp3c21_16160	AT4G25890	NaN	60S acidic ribosomal protein family
Pp3c21_20630	AT5G18380	NaN	Ribosomal protein S5 domain 2-like superfamily protein
Pp3c21_21510	AT4G34670	NaN	Ribosomal protein S3Ae
Pp3c21_21540	AT3G61110	ARS27A.RS27A	ribosomal protein S27
Pp3c22_1860	AT5G59240	NaN	Ribosomal protein S8e family protein
Pp3c22_15010	AT1G09640	NaN	Translation elongation factor EF1B. gamma chain
Pp3c22_15340	AT1G72550	NaN	tRNA synthetase beta subunit family protein
Pp3c22_18300	AT4G34670	NaN	Ribosomal protein S3Ae
Pp3c22_21090	AT5G18380	NaN	Ribosomal protein S5 domain 2-like superfamily protein
Pp3c22_22010	AT1G66580	RPL10C.SAG24	senescence associated gene 24
Pp3c23_4120	AT1G07320	RPL4	ribosomal protein L4
Pp3c23_4520	AT3G04400	emb2171	Ribosomal protein L14p/L23e family protein
Pp3c23_6150	AT2G37270	ATRPS5B.RPS5B	ribosomal protein 5B
Pp3c23_8220	AT1G23290	RPL27A.RPL27AB	Ribosomal protein L18e/L15 superfamily protein

Pp3.3 gene*	Best hit At gene*	At_symbol*	At_description*
Pp3c23_8410	AT3G57490	NaN	Ribosomal protein S5 family protein
Pp3c23_10040	AT5G27700	NaN	Ribosomal protein S21e
Pp3c23_12730	AT1G08360	NaN	Ribosomal protein L1p/L10e family
Pp3c23_15390	AT5G26710	NaN	Glutamyl/glutaminyl-tRNA synthetase. class Ic
Pp3c23_17260	AT1G52300	NaN	Zinc-binding ribosomal protein family protein
Pp3c23_17430	AT3G55620	emb1624	Translation initiation factor IF6
Pp3c23_18100	AT5G24510	NaN	60S acidic ribosomal protein family
Pp3c23_18210	AT3G17465	RPL3P	ribosomal protein L3 plastid
Pp3c23_18280	AT1G48830	NaN	Ribosomal protein S7e family protein
Pp3c23_22060	AT2G20450	NaN	Ribosomal protein L14
Pp3c24_20	AT5G14660	ATDEF2.DEF2.PDF1B	peptide deformylase 1B
Pp3c24_2340	AT4G25740	NaN	RNA binding Plectin/S10 domain-containing protein
Pp3c24_3690	AT1G08360	NaN	Ribosomal protein L1p/L10e family
Pp3c24_5500	AT3G57490	NaN	Ribosomal protein S5 family protein
Pp3c24_5780	AT3G57490	NaN	Ribosomal protein S5 family protein
Pp3c24_8160	AT5G27700	NaN	Ribosomal protein S21e
Pp3c24_12530	AT4G11630	NaN	Ribosomal protein L19 family protein
Pp3c24_13910	AT3G55620	emb1624	Translation initiation factor IF6
Pp3c24_15540	AT4G23620	NaN	Ribosomal protein L25/Gln-tRNA synthetase. anti-codon-binding domain
Pp3c24_18220	AT5G16130	NaN	Ribosomal protein S7e family protein
Pp3c24_18680	AT3G01800	NaN	Ribosome recycling factor
Pp3c24_18790	AT2G34480	NaN	Ribosomal protein L18ae/LX family protein
Pp3c24_18880	AT2G37270	ATRPS5B.RPS5B	ribosomal protein 5B
Pp3c24_20890	AT4G27090	NaN	Ribosomal protein L14
Pp3c25_230	AT5G18790	NaN	Ribosomal protein L33 family protein
Pp3c25_2440	AT1G10840	TIF3H1	translation initiation factor 3 subunit H1
Pp3c25_2670	AT1G10840	TIF3H1	translation initiation factor 3 subunit H1
Pp3c25_3730	AT5G58420	NaN	Ribosomal protein S4 (RPS4A) family protein
Pp3c25_3736	AT5G58420	NaN	Ribosomal protein S4 (RPS4A) family protein
Pp3c25_8110	AT4G08350	GTA02.GTA2	global transcription factor group A2
Pp3c25_9320	AT1G77940	NaN	Ribosomal protein L7Ae/L30e/S12e/Gadd45 family protein
Pp3c25_11980	AT1G26880	NaN	Ribosomal protein L34e superfamily protein
Pp3c25_13260	AT1G70600	NaN	Ribosomal protein L18e/L15 superfamily protein
Pp3c26_330	AT3G62870	NaN	Ribosomal protein L7Ae/L30e/S12e/Gadd45 family protein
Pp3c26_350	AT2G47610	NaN	Ribosomal protein L7Ae/L30e/S12e/Gadd45 family protein
Pp3c26_360	AT3G62870	NaN	Ribosomal protein L7Ae/L30e/S12e/Gadd45 family protein
Pp3c26_2200	AT5G55140	NaN	ribosomal protein L30 family protein
Pp3c26_3000	AT5G27700	NaN	Ribosomal protein S21e
Pp3c26_10900	AT5G18110	NCBP	novel cap-binding protein
Pp3c26_11220	AT4G14320	NaN	Zinc-binding ribosomal protein family protein
Pp3c26_11290	AT4G11420	ATEIF3A-1.ATTIF3A1.EIF3A.EIF3A-1.TIF3A1	eukaryotic translation initiation factor 3A
Pp3c26_13720	AT5G02610	NaN	Ribosomal L29 family protein
Pp3c26_15390	AT3G10950	NaN	Zinc-binding ribosomal protein family protein
Pp3c27_190	AT5G20920	EIF2 BETA.EMB1401	eukaryotic translation initiation factor 2 beta subunit
Pp3c27_1320	AT5G35530	NaN	Ribosomal protein S3 family protein
Pp3c27_1920	AT5G15200	NaN	Ribosomal protein S4
Pp3c27_1950	AT3G25660	NaN	Amidase family protein
Pp3c27_2260	AT5G45775	NaN	Ribosomal L5P family protein
Pp3c27_2780	AT3G02080	NaN	Ribosomal protein S19e family protein
Pp3c27_2790	AT1G34360	NaN	translation initiation factor 3 (IF-3) family protein
Pp3c27_7320	AT1G50200	ACD.ALATS	Alanyl-tRNA synthetase
Pp3c27_7820	AT5G09500	NaN	Ribosomal protein S19 family protein
Pp3c27_8190	AT1G33140	PGY2	Ribosomal protein L6 family
Pp3s93_30	AT3G13920	EIF4A1.RH4.TIF4A1	eukaryotic translation initiation factor 4A1
Pp3s116_30	AT3G48930	EMB1080	Nucleic acid-binding. OB-fold-like protein

*Twenty-seven of 398 genes are depicted in red as the common DEGs both 1cell-DGE and 5'DGE.

Supplementary Table S12. DEGs in 24 h-high identified using 1cell-DGE categorized by the GO term "carbohydrate metabolic process".

Pp3.3 gene*	Best hit At gene*	At_symbol*	At_description*
Pp3c1_2420	AT4G03210	XTH9	xyloglucan endotransglucosylase/hydrolase 9
Pp3c1_5000	AT4G29130	ATHXK1.GIN2.HXK1	hexokinase 1
Pp3c1_13780	AT5G58050	SVL4	SHV3-like 4
Pp3c1_29650	AT2G01140	NaN	Aldolase superfamily protein
Pp3c1_29840	AT5G22130	PNT1	mannosyltransferase family protein
Pp3c1_32570	AT1G66430	NaN	pfkB-like carbohydrate kinase family protein
Pp3c1_39460	AT4G04040	MEE51	Phosphofructokinase family protein
Pp3c1_39880	AT5G62620	NaN	Galactosyltransferase family protein
Pp3c1_41250	AT3G03050	ATCSLD3.CSLD3.KJK	cellulose synthase-like D3
Pp3c1_41400	AT3G03050	ATCSLD3.CSLD3.KJK	cellulose synthase-like D3
Pp3c1_41710	AT5G57500	NaN	Galactosyltransferase family protein
Pp3c2_1270	AT4G23100	ATECS1.CAD2.GSH1.GSHA.PAD2.RML1	glutamate-cysteine ligase
Pp3c2_1330	AT3G03050	ATCSLD3.CSLD3.KJK	cellulose synthase-like D3
Pp3c2_11980	AT4G04040	MEE51	Phosphofructokinase family protein
Pp3c2_17700	AT2G01140	NaN	Aldolase superfamily protein
Pp3c2_18380	AT3G57220	NaN	Glycosyl transferase family 4 protein
Pp3c2_28200	AT4G26690	GPDL2.MRH5.SHV3	PLC-like phosphodiesterase family protein
Pp3c2_28550	AT5G66530	NaN	Galactose mutarotase-like superfamily protein
Pp3c2_31490	AT1G12240	ATBETAFRUCT4.VAC-INV	Glycosyl hydrolases family 32 protein
Pp3c3_3400	AT1G27600	I9H.IRX9-L	Nucleotide-diphospho-sugar transferases superfamily protein
Pp3c3_7980	AT1G75680	AtGH9B7.GH9B7	glycosyl hydrolase 9B7
Pp3c3_14050	AT4G09020	ATISA3.ISA3	isoamylase 3
Pp3c3_37810	AT2G39630	NaN	Nucleotide-diphospho-sugar transferases superfamily protein
Pp3c4_1020	AT2G22240	ATIPS2.ATMIPS2.MIPS2	myo-inositol-1-phosphate synthase 2
Pp3c4_9820	AT3G23770	NaN	O-Glycosyl hydrolases family 17 protein
Pp3c4_11270	AT4G13430	ATLEUC1.IIL1	isopropyl malate isomerase large subunit 1
Pp3c4_11860	AT3G22960	PKP-ALPHA.PKP1	Pyruvate kinase family protein
Pp3c4_15060	AT4G03550	ATGSL05.ATGSL5.GSL05.GSL5.PMR4	glucan synthase-like 5
Pp3c4_17856	AT3G07160	ATGSL10.CALS9.gsl10	glucan synthase-like 10
Pp3c4_20080	AT5G51460	ATTPPA	Haloacid dehalogenase-like hydrolase (HAD) superfamily protein
Pp3c4_23720	AT3G62830	ATUXS2.AUD1.UXS2	NAD(P)-binding Rossmann-fold superfamily protein
Pp3c4_26640	AT5G36890	BGLU42	beta glucosidase 42
Pp3c4_28130	AT5G66460	NaN	Glycosyl hydrolase superfamily protein
Pp3c5_4460	AT3G23640	HGL1	heteroglycan glucosidase 1
Pp3c5_5170	AT5G01930	NaN	Glycosyl hydrolase superfamily protein
Pp3c5_8810	AT2G36390	BE3.SBE2.1	starch branching enzyme 2.1
Pp3c5_10340	AT1G78570	ATRHM1.RHM1.ROL1	rhamnose biosynthesis 1
Pp3c5_10720	AT1G32930	NaN	Galactosyltransferase family protein
Pp3c6_80	AT1G16300	GAPCP-2	glyceraldehyde-3-phosphate dehydrogenase of plastid 2
Pp3c6_170	AT1G16300	GAPCP-2	glyceraldehyde-3-phosphate dehydrogenase of plastid 2
Pp3c6_10280	AT2G21170	PDTPI.TIM	triosephosphate isomerase
Pp3c6_12710	AT5G66280	GMD1	GDP-D-mannose 4,6-dehydratase 1
Pp3c6_14770	AT1G67070	DIN9.PMI2	Mannose-6-phosphate isomerase. type I
Pp3c6_15170	AT1G78570	ATRHM1.RHM1.ROL1	rhamnose biosynthesis 1
Pp3c6_15300	AT1G63000	NRS/ER.UER1	nucleotide-rhamnose synthase/epimerase-reductase
Pp3c6_15310	AT1G63000	NRS/ER.UER1	nucleotide-rhamnose synthase/epimerase-reductase
Pp3c6_19470	AT2G39630	NaN	Nucleotide-diphospho-sugar transferases superfamily protein
Pp3c6_22980	AT5G01260	NaN	Carbohydrate-binding-like fold
Pp3c7_19260	AT1G02000	GAE2	UDP-D-glucuronate 4-epimerase 2
Pp3c7_22420	AT4G18240	ATSS4.SS4.SSIV	starch synthase 4
Pp3c8_940	AT4G34480	NaN	O-Glycosyl hydrolases family 17 protein
Pp3c8_6520	AT1G10760	GWD.GWD1.SEX1.SOP.SOP1	Pyruvate phosphate dikinase. PEP/pyruvate binding domain
Pp3c8_6536	AT1G10760	GWD.GWD1.SEX1.SOP.SOP1	Pyruvate phosphate dikinase. PEP/pyruvate binding domain
Pp3c8_6700	AT2G05320	NaN	beta-1,2-N-acetylglucosaminyltransferase II
Pp3c8_15610	AT2G22480	PFK5	phosphofructokinase 5
Pp3c8_19540	AT5G13870	EXGT-A4.XTH5	xyloglucan endotransglucosylase/hydrolase 5
Pp3c9_2670	AT5G05170	ATCESA3.ATH-B.CESA3.CEV1.IXR1	Cellulose synthase family protein
Pp3c9_4143	AT2G13680	ATGSL02.CALS5.GLS2	callose synthase 5
Pp3c9_5060	AT5G51820	ATPGMP.PGM.PGM1.STF1	phosphoglucomutase
Pp3c9_6840	AT3G16910	AAE7.ACN1	acyl-activating enzyme 7
Pp3c9_15430	AT3G28340	GATL10	galacturonosyltransferase-like 10
Pp3c10_3450	AT5G13110	G6PD2	glucose-6-phosphate dehydrogenase 2
Pp3c10_6390	AT1G17890	GER2	NAD(P)-binding Rossmann-fold superfamily protein
Pp3c10_8510	AT2G47760	ALG3.AtALG3	asparagine-linked glycosylation 3
Pp3c10_9960	AT1G30000	MNS3	alpha-mannosidase 3
Pp3c10_11740	AT3G62830	ATUXS2.AUD1.UXS2	NAD(P)-binding Rossmann-fold superfamily protein
Pp3c10_19330	AT3G07160	ATGSL10.CALS9.gsl10	glucan synthase-like 10
Pp3c10_20867	AT3G07160	ATGSL10.CALS9.gsl10	glucan synthase-like 10
Pp3c11_8270	AT5G48300	ADG1.APS1	ADP glucose pyrophosphorylase 1
Pp3c11_14370	AT1G02000	GAE2	UDP-D-glucuronate 4-epimerase 2
Pp3c11_15280	AT4G03210	XTH9	xyloglucan endotransglucosylase/hydrolase 9
Pp3c12_320	AT5G52920	PKP-BETA1.PKP1.PKP2	plastidic pyruvate kinase beta subunit 1
Pp3c12_9050	AT3G62720	ATXT1.XT1.XXT1	xylosyltransferase 1
Pp3c12_13470	AT3G23770	NaN	O-Glycosyl hydrolases family 17 protein
Pp3c12_16120	AT3G22960	PKP-ALPHA.PKP1	Pyruvate kinase family protein
Pp3c12_17000	AT4G33770	NaN	Inositol 1,3,4-trisphosphate 5/6-kinase family protein
Pp3c12_20050	AT5G51460	ATTPPA	Haloacid dehalogenase-like hydrolase (HAD) superfamily protein
Pp3c13_11140	AT3G23640	HGL1	heteroglycan glucosidase 1
Pp3c13_12000	AT5G01930	NaN	Glycosyl hydrolase superfamily protein
Pp3c13_17460	AT5G04360	ATLDA.ATPU1.LDA.PU1	limit dextrinase
Pp3c13_23550	AT2G29560	ENOC	cytosolic enolase
Pp3c13_24600	AT1G64390	AtGH9C2.GH9C2	glycosyl hydrolase 9C2

Pp3.3 gene*	Best hit At gene*	At_symbol*	At_description*
Pp3c14_2990	AT5G13110	G6PD2	glucose-6-phosphate dehydrogenase 2
Pp3c14_11550	AT1G30620	HSR8.MUR4.UXE1	NAD(P)-binding Rossmann-fold superfamily protein
Pp3c14_13300	AT5G13110	G6PD2	glucose-6-phosphate dehydrogenase 2
Pp3c14_15630	AT3G62830	ATUXS2.AUD1.UXS2	NAD(P)-binding Rossmann-fold superfamily protein
Pp3c14_16940	AT5G58330	NaN	lactate/malate dehydrogenase family protein
Pp3c14_17100	AT4G03210	XTH9	xyloglucan endotransglucosylase/hydrolase 9
Pp3c14_17850	AT5G42810	ATIPK1.IPK1	inositol-pentakisphosphate 2-kinase 1
Pp3c14_20670	AT2G26100	NaN	Galactosyltransferase family protein
Pp3c14_26100	AT3G03050	ATCSLD3.CSLD3.KJK	cellulose synthase-like D3
Pp3c14_26580	AT5G55500	ATXYLT.XYLT	beta-1,2-xylosyltransferase
Pp3c15_3000	AT1G67140	SWEETIE	HEAT repeat-containing protein
Pp3c15_8520	AT1G01090	PDH-E1 ALPHA	pyruvate dehydrogenase E1 alpha
Pp3c16_4030	AT1G66430	NaN	pfkB-like carbohydrate kinase family protein
Pp3c16_5490	AT1G67070	DIN9.PMI2	Mannose-6-phosphate isomerase. type I
Pp3c16_7970	AT1G78570	ATRHM1.RHM1.ROL1	rhamnose biosynthesis 1
Pp3c16_11330	AT2G21170	PDTPI.TIM	triosephosphate isomerase
Pp3c16_13360	AT2G36390	BE3.SBE2.1	starch branching enzyme 2.1
Pp3c16_19380	AT1G22170	NaN	Phosphoglycerate mutase family protein
Pp3c17_2240	AT3G61130	GAUT1.LGT1	galacturonosyltransferase 1
Pp3c17_3060	AT5G13870	EXGT-A4.XTH5	xyloglucan endotransglucosylase/hydrolase 5
Pp3c17_8050	AT5G08570	NaN	Pyruvate kinase family protein
Pp3c17_13270	AT1G05170	NaN	Galactosyltransferase family protein
Pp3c17_13610	AT1G34130	STT3B	staurosporin and temperature sensitive 3-like b
Pp3c17_20310	AT5G58330	NaN	lactate/malate dehydrogenase family protein
Pp3c17_22380	AT3G03050	ATCSLD3.CSLD3.KJK	cellulose synthase-like D3
Pp3c18_9850	AT1G76550	NaN	Phosphofructokinase family protein
Pp3c18_20850	AT1G32900	NaN	UDP-Glycosyltransferase superfamily protein
Pp3c19_4120	AT5G63840	PSL5.RSW3	Glycosyl hydrolases family 31 protein
Pp3c19_7712	AT3G01180	AtSS2.SS2	starch synthase 2
Pp3c19_8410	AT1G27680	APL2	ADPGLC-PPase large subunit
Pp3c19_19220	AT5G54570	BGLU41	beta glucosidase 41
Pp3c20_2940	AT5G53340	NaN	Galactosyltransferase family protein
Pp3c20_7860	AT4G00490	BAM2.BMY9	beta-amylase 2
Pp3c20_13380	AT2G22480	PFK5	phosphofructokinase 5
Pp3c20_17110	AT3G62710	NaN	Glycosyl hydrolase family protein
Pp3c20_17800	AT5G64860	DPE1	disproportionating enzyme
Pp3c21_8340	AT3G02870	VTC4	Inositol monophosphatase family protein
Pp3c21_16140	AT1G76550	NaN	Phosphofructokinase family protein
Pp3c21_19280	AT4G29130	ATHXK1.GIN2.HXK1	hexokinase 1
Pp3c22_90	AT3G61130	GAUT1.LGT1	galacturonosyltransferase 1
Pp3c22_470	AT1G26810	GALT1	galactosyltransferase1
Pp3c22_2470	AT4G31140	NaN	O-Glycosyl hydrolases family 17 protein
Pp3c22_2590	AT3G04240	SEC	Tetratricopeptide repeat (TPR)-like superfamily protein
Pp3c22_9700	AT4G00490	BAM2.BMY9	beta-amylase 2
Pp3c22_21700	AT3G22960	PKP-ALPHA.PKP1	Pyruvate kinase family protein
Pp3c23_3680	AT1G51590	MANIB.MNS1	alpha-mannosidase 1
Pp3c23_9440	AT3G01040	GAUT13	galacturonosyltransferase 13
Pp3c23_9830	AT3G28340	GATL10	galacturonosyltransferase-like 10
Pp3c23_11670	AT2G45790	ATPMM.PMM	phosphomannomutase
Pp3c23_18810	AT5G01260	NaN	Carbohydrate-binding-like fold
Pp3c24_840	AT5G17520	MEX1.RCP1	root cap 1 (RCP1)
Pp3c24_3950	AT3G01040	GAUT13	galacturonosyltransferase 13
Pp3c24_6930	AT5G66680	DGL1	dolichyl-diphosphooligosaccharide-protein glycosyltransferase 48kDa subunit family protein
Pp3c24_8780	AT3G27300	G6PD5	glucose-6-phosphate dehydrogenase 5
Pp3c24_11070	AT5G64380	NaN	Inositol monophosphatase family protein
Pp3c24_13530	AT5G67540	NaN	Arabinanase/levansucrase/invertase
Pp3c24_16550	AT1G15380	NaN	Lactoylglutathione lyase / glyoxalase I family protein
Pp3c24_17310	AT5G24400	EMB2024.PGL3	NagB/RpiA/CoA transferase-like superfamily protein
Pp3c25_4050	AT4G03210	XTH9	xyloglucan endotransglucosylase/hydrolase 9
Pp3c25_4070	AT4G03210	XTH9	xyloglucan endotransglucosylase/hydrolase 9
Pp3c25_13970	AT1G79530	GAPCP-1	glyceraldehyde-3-phosphate dehydrogenase of plastid 1
Pp3c25_15030	AT5G13980	NaN	Glycosyl hydrolase family 38 protein
Pp3c26_9330	AT1G68560	ATXYL1.TRG1.XYL1	alpha-xylosidase 1
Pp3c26_14180	AT2G20370	KAM1.MUR3	Exostosin family protein

*Fifteen of 146 genes are colored in red as the common DEGs both 1cell-DGE and 5'DGE.

Supplementary Table S13. DEGs in 24 h-high identified using 1cell-DGE categorized by the GO term "anatomical structure morphogenesis".

Pp3.3 gene*	Best hit At gene*	At_symbol*	At_description*
Pp3c1_60	AT4G20910	CRM2.HEN1	double-stranded RNA binding protein-related / DsRBD protein-related
Pp3c1_860	AT3G01460	ATMBD9.MBD9	methyl-CPG-binding domain 9
Pp3c1_6150	AT2G35110	GRL.NAP1.NAPP	transcription activators
Pp3c1_6160	AT4G00100	ATRPS13A.PFL2.RPS13.RPS13A	ribosomal protein S13A
Pp3c1_6880	AT5G22220	ATE2FB.E2F1.E2FB	E2F transcription factor 1
Pp3c1_7160	AT5G39740	OLI7.RPL5B	ribosomal protein L5 B
Pp3c1_13010	AT5G45190	NaN	Cyclin family protein
Pp3c1_21120	AT5G67100	ICU2	DNA-directed DNA polymerases
Pp3c1_35770	AT1G48410	AGO1	Stabilizer of iron transporter SufD / Polynucleotidyl transferase
Pp3c1_35990	AT4G34460	AGB1.ATAGB1.ELK4	GTP binding protein beta 1
Pp3c1_37980	AT4G28250	ATEXPB3.ATHEXP BETA 1.6.EXPB3	expansin B3
Pp3c1_39840	AT2G38440	ATSCAR2.DIS3.ITB1.SCAR2.WAVE4	SCAR homolog 2
Pp3c2_4060	AT1G48410	AGO1	Stabilizer of iron transporter SufD / Polynucleotidyl transferase
Pp3c2_8430	AT2G38440	ATSCAR2.DIS3.ITB1.SCAR2.WAVE4	SCAR homolog 2
Pp3c2_11270	AT1G05180	AXR1	NAD(P)-binding Rossmann-fold superfamily protein
Pp3c2_16690	AT5G18700	EMB3013.RUK	Protein kinase family protein with ARM repeat domain
Pp3c2_28200	AT4G26690	GPDL2.MRH5.SHV3	PLC-like phosphodiesterase family protein
Pp3c2_31540	AT5G39740	OLI7.RPL5B	ribosomal protein L5 B
Pp3c2_34930	AT1G74030	ENO1	enolase 1
Pp3c2_35750	AT3G01460	ATMBD9.MBD9	methyl-CPG-binding domain 9
Pp3c3_270	AT5G57020	ATNMT1.NMT1	myristoyl-CoA:protein N-myristoyltransferase
Pp3c3_19880	AT4G14147	ARPC4	protein binding
Pp3c3_24130	AT5G55540	LOP1.TRN1	tornado 1
Pp3c3_31010	AT3G51460	RHD4	Phosphoinositide phosphatase family protein
Pp3c3_32980	AT3G07880	SCN1	Immunoglobulin E-set superfamily protein
Pp3c3_33440	AT5G09810	ACT7	actin 7
Pp3c4_1720	AT4G34490	ATCAP1.CAP 1.CAP1	cyclase associated protein 1
Pp3c4_6230	AT5G09810	ACT7	actin 7
Pp3c4_7260	AT1G69780	ATHB13	Homeobox-leucine zipper protein family
Pp3c4_12350	AT1G75500	WAT1	Walls Are Thin 1
Pp3c4_15060	AT4G03550	ATGSL05.ATGSL5.GSL05.GSL5.PMR4	glucan synthase-like 5
Pp3c5_2890	AT1G03060	SPI	Beige/BEACH domain ;WD domain. G-beta repeat protein
Pp3c5_6270	AT5G19530	ACL5	S-adenosyl-L-methionine-dependent methyltransferases superfamily protein
Pp3c5_6600	AT2G13540	ABH1.ATCBP80.CBP80.ENS	ARM repeat superfamily protein
Pp3c5_22030	AT1G12980	DRN.ESR1	Integrase-type DNA-binding superfamily protein
Pp3c6_5706	AT1G03060	SPI	Beige/BEACH domain ;WD domain. G-beta repeat protein
Pp3c6_6290	AT5G18580	EMB40.FASS.FASS 2.FS1.GDO.TON2	tonneau 2 (TON2)
Pp3c6_6440	AT3G18730	BRU1.MGO3.TSK	tetratricopeptide repeat (TPR)-containing protein
Pp3c6_10010	AT3G46740	MAR1.TOC75-III	translocon at the outer envelope membrane of chloroplasts 75-III
Pp3c6_20680	AT4G38630	ATMCB1.MBP1.MCB1.RPN10	regulatory particle non-ATPase 10
Pp3c6_25960	AT1G50240	FU	Protein kinase family protein with ARM repeat domain
Pp3c6_26100	AT1G03060	SPI	Beige/BEACH domain ;WD domain. G-beta repeat protein
Pp3c6_27380	AT5G19530	ACL5	S-adenosyl-L-methionine-dependent methyltransferases superfamily protein
Pp3c7_650	AT3G19820	CBB1.DIM.DIM1.DWF1.EVE1	cell elongation protein / DWARF1 / DIMINUTO (DIM)
Pp3c7_3740	AT3G05040	HST.HST1	ARM repeat superfamily protein
Pp3c7_8710	AT5G04240	ELF6	Zinc finger (C2H2 type) family protein / transcription factor jumonji (jmj) family protein
Pp3c7_12810	AT5G56320	ATEXP14.ATEXPA14.ATHEXP ALPHA 1.5.EXP14.EXPA14	expansin A14
Pp3c7_12870	AT5G56320	ATEXP14.ATEXPA14.ATHEXP ALPHA 1.5.EXP14.EXPA14	expansin A14
Pp3c7_23430	AT3G46740	MAR1.TOC75-III	translocon at the outer envelope membrane of chloroplasts 75-III
Pp3c7_25660	AT5G39740	OLI7.RPL5B	ribosomal protein L5 B
Pp3c7_26720	AT4G00100	ATRPS13A.PFL2.RPS13.RPS13A	ribosomal protein S13A
Pp3c8_870	AT1G69530	AT-EXP1.ATEXP1.ATEXPA1.ATHEXP ALPHA 1.2.EXP1.EXPA1	expansin A1
Pp3c8_12060	AT2G18390	ARL2.ATARLC1.HAL.TTN5	ADP-ribosylation factor family protein
Pp3c8_13450	AT1G69530	AT-EXP1.ATEXP1.ATEXPA1.ATHEXP ALPHA 1.2.EXP1.EXPA1	expansin A1
Pp3c8_13470	AT1G69530	AT-EXP1.ATEXP1.ATEXPA1.ATHEXP ALPHA 1.2.EXP1.EXPA1	expansin A1
Pp3c8_14900	AT5G56320	ATEXP14.ATEXPA14.ATHEXP ALPHA 1.5.EXP14.EXPA14	expansin A14
Pp3c9_2440	AT3G12280	ATRBR1.RB.RB1.RBR.RBR1	retinoblastoma-related 1
Pp3c9_2830	AT5G26240	ATCLC-D.CLC-D	chloride channel D
Pp3c9_4143	AT2G13680	ATGSL02.CALS5.GLS2	callose synthase 5
Pp3c9_7720	AT2G06850	EXGT-A1.EXT.XTH4	xyloglucan endotransglucosylase/hydrolase 4
Pp3c9_11830	AT1G13980	EMB30.GN.VAN7	sec7 domain-containing protein
Pp3c9_21250	AT1G80490	TPR1	TOPLESS-related 1
Pp3c9_23630	AT1G16890	UBC13B.UBC36	ubiquitin-conjugating enzyme 36
Pp3c10_5440	AT5G17020	ATCRM1.ATXPO1.HIT2.XPO1.XPO1A	exportin 1A
Pp3c10_17070	AT5G09810	ACT7	actin 7
Pp3c10_20720	AT5G55540	LOP1.TRN1	tornado 1
Pp3c11_1080	AT2G35110	GRL.NAP1.NAPP	transcription activators
Pp3c11_1100	AT4G00100	ATRPS13A.PFL2.RPS13.RPS13A	ribosomal protein S13A
Pp3c11_6420	AT5G39740	OLI7.RPL5B	ribosomal protein L5 B
Pp3c11_12000	AT5G56320	ATEXP14.ATEXPA14.ATHEXP ALPHA 1.5.EXP14.EXPA14	expansin A14
Pp3c11_24520	AT5G18560	PUCHI	Integrase-type DNA-binding superfamily protein
Pp3c11_26120	AT3G19820	CBB1.DIM.DIM1.DWF1.EVE1	cell elongation protein / DWARF1 / DIMINUTO (DIM)
Pp3c12_4560	AT5G02260	ATEXP9.ATEXPA9.ATHEXP ALPHA 1.10.EXP9.EXPA9	expansin A9
Pp3c12_8200	AT3G54010	DEI1.PAS1	FKBP-type peptidyl-prolyl cis-trans isomerase family protein
Pp3c12_9050	AT3G62720	ATXT1.XT1.XXT1	xylosyltransferase 1
Pp3c12_16340	AT5G07280	EMS1.EXS	Leucine-rich repeat transmembrane protein kinase
Pp3c12_21910	AT1G72560	PSD	ARM repeat superfamily protein
Pp3c12_23740	AT4G22910	CCS52A1.FZR2	FIZZY-related 2
Pp3c13_1280	AT5G57020	ATNMT1.NMT1	myristoyl-CoA:protein N-myristoyltransferase
Pp3c13_14220	AT1G12840	ATVHA-C.DET3	vacuolar ATP synthase subunit C (VATC) / V-ATPase C subunit (DET3)
Pp3c13_20360	AT5G02260	ATEXP9.ATEXPA9.ATHEXP ALPHA 1.10.EXP9.EXPA9	expansin A9
Pp3c13_22280	AT4G05410	YAO	Transducin/WD40 repeat-like superfamily protein
Pp3c14_1640	AT5G27540	emb2473.MIRO1	MIRO-related GTP-ase 1
Pp3c14_3850	AT5G18700	EMB3013.RUK	Protein kinase family protein with ARM repeat domain
Pp3c14_7440	AT1G05180	AXR1	NAD(P)-binding Rossmann-fold superfamily protein
Pp3c14_11530	AT2G34680	AIR9	Outer arm dynein light chain 1 protein
Pp3c14_18440	AT5G56320	ATEXP14.ATEXPA14.ATHEXP ALPHA 1.5.EXP14.EXPA14	expansin A14
Pp3c14_18590	AT5G56320	ATEXP14.ATEXPA14.ATHEXP ALPHA 1.5.EXP14.EXPA14	expansin A14
Pp3c15_1000	AT4G05410	YAO	Transducin/WD40 repeat-like superfamily protein
Pp3c15_3730	AT5G65930	KCBP.PKCBP.ZWI	kinesin-like calmodulin-binding protein (ZWICHEL)
Pp3c15_11320	AT1G13980	EMB30.GN.VAN7	sec7 domain-containing protein
Pp3c15_17790	AT3G57670	NTT.WIP2	C2H2-type zinc finger family protein
Pp3c16_14030	AT1G71696	SOL1	carboxypeptidase D. putative
Pp3c16_15220	AT4G00100	ATRPS13A.PFL2.RPS13.RPS13A	ribosomal protein S13A
Pp3c16_16110	AT5G05780	AE3.ATHMOV34.RPN8A	RP non-ATPase subunit 8A
Pp3c16_16290	AT5G27540	emb2473.MIRO1	MIRO-related GTP-ase 1
Pp3c17_1180	AT2G42620	MAX2.ORE9.PPS	RNI-like superfamily protein
Pp3c17_7410	AT2G34680	AIR9	Outer arm dynein light chain 1 protein
Pp3c17_12980	AT4G28250	ATEXPB3.ATHEXP BETA 1.6.EXPB3	expansin B3

Pp3.3 gene*	Best hit At gene*	At_symbol*	At_description*
Pp3c18_12190	AT3G60830	ARP7.ATARP7	actin-related protein 7
Pp3c18_15140	AT4G24670	TAR2	tryptophan aminotransferase related 2
Pp3c18_19660	AT5G02260	ATEXP9.ATEXPA9.ATHEXP ALPHA 1.10.EXP9.EXPA9	expansin A9
Pp3c18_20160	AT5G23530	AtCXE18.CXE18	carboxyesterase 18
Pp3c18_20180	AT3G16640	TCTP	translationally controlled tumor protein
Pp3c19_4120	AT5G63840	PSL5.RSW3	Glycosyl hydrolases family 31 protein
Pp3c19_4870	AT1G14830	ADL1C.ADL5.DL1C.DRP1C	DYNAMIN-like 1C
Pp3c19_7110	AT3G61650	TUBG1	gamma-tubulin
Pp3c20_6790	AT4G27060	CN.SPR2.TOR1	ARM repeat superfamily protein
Pp3c20_7710	AT5G63090	LOB	Lateral organ boundaries (LOB) domain family protein
Pp3c20_8320	AT5G48030	GFA2	gametophytic factor 2
Pp3c20_15610	AT3G12160	ATRABA4D.RABA4D	RAB GTPase homolog A4D
Pp3c20_17290	AT5G54380	THE1	protein kinase family protein
Pp3c21_530	AT5G64930	CPR5.HYS1	CPR5 protein. putative
Pp3c21_3680	AT3G16640	TCTP	translationally controlled tumor protein
Pp3c21_8020	AT5G18410	ATSRA1.KLK.PIR.PIR121.PIRP.SRA1	transcription activators
Pp3c21_11720	AT3G13870	RHD3	Root hair defective 3 GTP-binding protein (RHD3)
Pp3c21_15710	AT1G74380	XXT5	xyloglucan xylosyltransferase 5
Pp3c22_630	AT4G28250	ATEXPB3.ATHEXP BETA 1.6.EXPB3	expansin B3
Pp3c22_1100	AT1G14830	ADL1C.ADL5.DL1C.DRP1C	DYNAMIN-like 1C
Pp3c22_2850	AT3G61650	TUBG1	gamma-tubulin
Pp3c22_5670	AT5G64930	CPR5.HYS1	CPR5 protein. putative
Pp3c22_5980	AT5G58230	ATMSI1.MEE70.MSI1	Transducin/WD40 repeat-like superfamily protein
Pp3c22_23030	AT1G74380	XXT5	xyloglucan xylosyltransferase 5
Pp3c23_5320	AT5G26240	ATCLC-D.CLC-D	chloride channel D
Pp3c23_7840	AT3G12160	ATRABA4D.RABA4D	RAB GTPase homolog A4D
Pp3c23_8340	AT1G63700	EMB71.MAPKKK4.YDA	Protein kinase superfamily protein
Pp3c23_15370	AT5G48030	GFA2	gametophytic factor 2
Pp3c24_6080	AT1G63700	EMB71.MAPKKK4.YDA	Protein kinase superfamily protein
Pp3c24_6930	AT5G66680	DGL1	dolichyl-diphosphooligosaccharide-protein glycosyltransferase 48kDa subunit family protein
Pp3c24_19870	AT4G33650	ADL2.DRP3A	dynamin-related protein 3A
Pp3c25_4250	AT1G12980	DRN.ESR1	Integrase-type DNA-binding superfamily protein
Pp3c26_9880	AT5G18560	PUCHI	Integrase-type DNA-binding superfamily protein
Pp3c26_14180	AT2G20370	KAM1.MUR3	Exostosin family protein
Pp3c27_1990	AT1G04950	ATTAF6.TAF6.TAFII59	TATA BOX ASSOCIATED FACTOR II 59
Pp3c27_3260	AT5G19530	ACL5	S-adenosyl-L-methionine-dependent methyltransferases superfamily protein
Pp3c27_8050	AT4G33880	RSL2	ROOT HAIR DEFECTIVE 6-LIKE 2

*Twenty of 136 genes are colored in red as the common DEGs both 1cell-DGE and 5'DGE.

Supplementary Table S14. DEGs in 24 h-high identified using 1cell-DGE categorized by the GO term "cellular component organization".

Pp3.3 gene*	Best hit At gene*	At_symbol*	At_description*
Pp3c1_1000	AT1G21600	PTAC6	plastid transcriptionally active 6
Pp3c1_6150	AT2G35110	GRL.NAP1.NAPP	transcription activators
Pp3c1_6160	AT4G00100	ATRPS13A.PFL2.RPS13.RPS13A	ribosomal protein S13A
Pp3c1_6880	AT5G22220	ATE2FB.E2F1.E2FB	E2F transcription factor 1
Pp3c1_8440	AT1G59610	ADL3.CF1.DL3.DRP2B	dynamin-like 3
Pp3c1_10010	AT5G64050	ATERS.ERS.OVA3	glutamate tRNA synthetase
Pp3c1_13010	AT5G45190	NaN	Cyclin family protein
Pp3c1_17880	AT3G07100	ERMO2.SEC24A	Sec23/Sec24 protein transport family protein
Pp3c1_22480	AT4G01370	ATMPK4.MPK4	MAP kinase 4
Pp3c1_23960	AT1G61290	ATSYP124.SYP124	syntaxin of plants 124
Pp3c1_24120	AT1G72370	AP40.P40.RP40.RPSAA	40s ribosomal protein SA
Pp3c1_31160	AT2G26890	GRV2.KAM2	DNAJ heat shock N-terminal domain-containing protein
Pp3c1_32090	AT5G58270	ATATM3.ATM3.STA1	ABC transporter of the mitochondrion 3
Pp3c1_32220	AT2G16070	PDV2	plastid division2
Pp3c1_35930	AT2G29530	TIM10	Tim10/DDP family zinc finger protein
Pp3c1_37980	AT4G28250	ATEXPB3.ATHEXP BETA 1.6.EXPB3	expansin B3
Pp3c1_39350	AT4G32830	AtAUR1.AUR1	ataurora1
Pp3c1_39840	AT2G38440	ATSCAR2.DIS3.ITB1.SCAR2.WAVE4	SCAR homolog 2
Pp3c1_41070	AT5G08120	MPB2C	movement protein binding protein 2C
Pp3c2_8420	AT5G61670	NaN	NaN
Pp3c2_8430	AT2G38440	ATSCAR2.DIS3.ITB1.SCAR2.WAVE4	SCAR homolog 2
Pp3c2_11870	AT2G25880	AtAUR2.AUR2	ataurora2
Pp3c2_13760	AT5G35520	ATMIS12.MIS12	minichromosome instability 12 (mis12)-like
Pp3c2_16540	AT2G22740	SDG23.SUVH6	SU(VAR)3-9 homolog 6
Pp3c2_16690	AT5G18700	EMB3013.RUK	Protein kinase family protein with ARM repeat domain
Pp3c2_18070	AT3G60740	CHO.EMB133.TFC D.TTN1	ARM repeat superfamily protein
Pp3c2_19250	AT5G40660	NaN	ATP12 protein-related
Pp3c2_22550	AT3G08960	NaN	ARM repeat superfamily protein
Pp3c2_28200	AT4G26690	GPDL2.MRH5.SHV3	PLC-like phosphodiesterase family protein
Pp3c2_30830	AT2G22740	SDG23.SUVH6	SU(VAR)3-9 homolog 6
Pp3c2_32060	AT1G59610	ADL3.CF1.DL3.DRP2B	dynamin-like 3
Pp3c2_33000	AT3G19720	ARC5.DRP5B	P-loop containing nucleoside triphosphate hydrolases superfamily protein
Pp3c2_34930	AT1G74030	ENO1	enolase 1
Pp3c3_2280	AT4G12610	ATRAP74.RAP74	transcription activators;DNA binding;RNA polymerase II transcription factors
Pp3c3_3110	AT5G05970	NEDD1	Transducin/WD40 repeat-like superfamily protein
Pp3c3_5690	AT4G30930	NFD1	Ribosomal protein L21
Pp3c3_5880	AT2G19480	NAP1;2.NFA02.NFA2	nucleosome assembly protein 1;2
Pp3c3_10170	AT1G31480	SGR2	shoot gravitropism 2 (SGR2)
Pp3c3_11140	AT3G52750	FTSZ2-2	Tubulin/FtsZ family protein
Pp3c3_16200	AT2G26890	GRV2.KAM2	DNAJ heat shock N-terminal domain-containing protein
Pp3c3_19880	AT4G14147	ARPC4	protein binding
Pp3c3_29950	AT1G15220	ATCCMH.CCMH	cytochrome c biogenesis protein family
Pp3c3_31010	AT3G51460	RHD4	Phosphoinositide phosphatase family protein
Pp3c3_32130	AT1G26170	NaN	ARM repeat superfamily protein
Pp3c3_32300	AT3G57060	NaN	binding
Pp3c3_32980	AT3G07880	SCN1	Immunoglobulin E-set superfamily protein
Pp3c3_33440	AT5G09810	ACT7	actin 7
Pp3c4_1720	AT4G34490	ATCAP1.CAP 1.CAP1	cyclase associated protein 1
Pp3c4_6230	AT5G09810	ACT7	actin 7
Pp3c4_10660	AT2G19080	NaN	metaxin-related
Pp3c4_12350	AT1G75500	WAT1	Walls Are Thin 1
Pp3c4_14910	AT2G04270	RNEE/G	RNAse E/G-like
Pp3c4_17856	AT3G07160	ATGSL10.CALS9.gsl10	glucan synthase-like 10
Pp3c4_19960	AT2G36070	ATTIM44-2.TIM44-2	translocase inner membrane subunit 44-2
Pp3c4_7610	AT4G27640	NaN	ARM repeat superfamily protein
Pp3c5_2890	AT1G03060	SPI	Beige/BEACH domain ;WD domain. G-beta repeat protein
Pp3c5_4480	AT3G17910	SURF1	Surfeit locus 1 cytochrome c oxidase biogenesis protein
Pp3c5_6270	AT5G19530	ACL5	S-adenosyl-L-methionine-dependent methyltransferases superfamily protein
Pp3c5_7300	AT1G54385	NaN	ARM repeat superfamily protein
Pp3c5_16690	AT2G01730	ATCPSF73-II.CPSF73-II.EDA26	cleavage and polyadenylation specificity factor 73 kDa subunit-II
Pp3c5_17200	AT3G08950	NaN	electron transport SCO1/SenC family protein
Pp3c5_23010	AT5G50920	ATHSP93-V.CLPC.CLPC1.DCA1.HSP93-V	CLPC homologue 1
Pp3c5_23930	AT1G65440	GTB1	global transcription factor group B1
Pp3c5_27640	AT5G45390	CLPP4.NCLPP4	CLP protease P4
Pp3c6_2930	AT2G18950	ATHPT.HPT1.TPT1.VTE2	homogentisate phytyltransferase 1
Pp3c6_3460	AT5G40820	ATATR.ATR.ATRAD3	Ataxia telangiectasia-mutated and RAD3-related
Pp3c6_5706	AT1G03060	SPI	Beige/BEACH domain ;WD domain. G-beta repeat protein
Pp3c6_6290	AT5G18580	EMB40.FASS.FASS 2.FS1.GDO.TON2	tonneau 2 (TON2)
Pp3c6_10010	AT3G46740	MAR1.TOC75-III	translocon at the outer envelope membrane of chloroplasts 75-III
Pp3c6_10280	AT2G21170	PDTPI.TIM	triosephosphate isomerase
Pp3c6_20680	AT4G38630	ATMCB1.MBP1.MCB1.RPN10	regulatory particle non-ATPase 10
Pp3c6_26100	AT1G03060	SPI	Beige/BEACH domain ;WD domain. G-beta repeat protein
Pp3c6_27380	AT5G19530	ACL5	S-adenosyl-L-methionine-dependent methyltransferases superfamily protein
Pp3c7_650	AT3G19820	CBB1.DIM.DIM1.DWF1.EVE1	cell elongation protein / DWARF1 / DIMINUTO (DIM)
Pp3c7_5160	AT5G53480	NaN	ARM repeat superfamily protein
Pp3c7_5660	AT3G61140	ATFUS6.ATSK31.COP11.CSN1.EMB78.FUS6.SK31	26S proteasome. regulatory subunit Rpn7;Proteasome component (PCI) domain
Pp3c7_5710	AT5G56090	COX15	cytochrome c oxidase 15
Pp3c7_6470	AT5G09420	ATTOC64-V.MTOM64.TOC64-V	translocon at the outer membrane of chloroplasts 64-V
Pp3c7_8710	AT5G04240	ELF6	Zinc finger (C2H2 type) family protein / transcription factor jumonji (jmj) family protein
Pp3c7_10880	AT2G30260	U2Bå´'å´'	U2 small nuclear ribonucleoprotein B
Pp3c7_12810	AT5G56320	ATEXP14.ATEXPA14.ATHEXP ALPHA 1.5.EXP14.EXPA14	expansin A14
Pp3c7_12870	AT5G56320	ATEXP14.ATEXPA14.ATHEXP ALPHA 1.5.EXP14.EXPA14	expansin A14
Pp3c7_15020	AT4G25340	ATFKBP53.FKBP53	FK506 BINDING PROTEIN 53
Pp3c7_15024	AT4G25340	ATFKBP53.FKBP53	FK506 BINDING PROTEIN 53
Pp3c7_19554	AT4G14150	KINESIN-12A.PAKRP1	phragmoplast-associated kinesin-related protein 1
Pp3c7_23430	AT3G46740	MAR1.TOC75-III	translocon at the outer envelope membrane of chloroplasts 75-III
Pp3c7_24000	AT4G37280	NaN	MRG family protein
Pp3c7_26720	AT4G00100	ATRPS13A.PFL2.RPS13.RPS13A	ribosomal protein S13A
Pp3c8_870	AT1G69530	AT-EXP1.ATEXP1.ATEXPA1.ATHEXP ALPHA 1.2.EXP1.EXPA1	expansin A1
Pp3c8_7540	AT1G72440	EDA25.SWA2	CCAAT-binding factor
Pp3c8_10370	AT1G78900	VHA-A	vacuolar ATP synthase subunit A
Pp3c8_12060	AT2G18390	ARL2.ATARLC1.HAL.TTN5	ADP-ribosylation factor family protein
Pp3c8_13300	AT5G59230	NaN	transcription factor-related

Pp3.3 gene*	Best hit At gene*	At_symbol*	At_description*
Pp3c8_13450	AT1G69530	AT-EXP1.ATEXP1.ATEXPA1.ATHEXP ALPHA 1.2.EXP1.EXPA1	expansin A1
Pp3c8_13470	AT1G69530	AT-EXP1.ATEXP1.ATEXPA1.ATHEXP ALPHA 1.2.EXP1.EXPA1	expansin A1
Pp3c8_14900	AT5G56320	ATEXP14.ATEXPA14.ATHEXP ALPHA 1.5.EXP14.EXPA14	expansin A14
Pp3c8_20390	AT5G08470	PEX1	peroxisome 1
Pp3c8_25300	AT5G40770	ATPHB3.PHB3	prohibitin 3
Pp3c9_950	AT3G26618	ERF1-3	eukaryotic release factor 1-3
Pp3c9_2440	AT3G12280	ATRBR1.RB.RB1.RBR.RBR1	retinoblastoma-related 1
Pp3c9_2830	AT5G26240	ATCLC-D.CLC-D	chloride channel D
Pp3c9_4143	AT2G13680	ATGSL02.CALS5.GLS2	callose synthase 5
Pp3c9_6030	AT1G20830	MCD1	multiple chloroplast division site 1
Pp3c9_7720	AT2G06850	EXGT-A1.EXT.XTH4	xyloglucan endotransglucosylase/hydrolase 4
Pp3c9_11830	AT1G13980	EMB30.GN.VAN7	sec7 domain-containing protein
Pp3c9_18560	AT1G02090	ATCSN7.COP15.CSN7.FUS5	Proteasome component (PCI) domain protein
Pp3c9_22260	AT2G31660	SAD2.URM9	ARM repeat superfamily protein
Pp3c10_5440	AT5G17020	ATCRM1.ATXPO1.HIT2.XPO1.XPO1A	exportin 1A
Pp3c10_8380	AT5G10490	MSL2	MSCS-like 2
Pp3c10_9060	AT1G21700	ATSWI3C.CHB4.SWI3C	SWITCH/sucrose nonfermenting 3C
Pp3c10_13610	AT1G54690	G-H2AX.GAMMA-H2AX.H2AXB.HTA3	gamma histone variant H2AX
Pp3c10_17070	AT5G09810	ACT7	actin 7
Pp3c10_17110	AT2G28800	ALB3	63 kDa inner membrane family protein
Pp3c10_19330	AT3G07160	ATGSL10.CALS9.gsl10	glucan synthase-like 10
Pp3c10_20867	AT3G07160	ATGSL10.CALS9.gsl10	glucan synthase-like 10
Pp3c11_1080	AT2G35110	GRL.NAP1.NAPP	transcription activators
Pp3c11_1100	AT4G00100	ATRPS13A.PFL2.RPS13.RPS13A	ribosomal protein S13A
Pp3c11_12000	AT5G56320	ATEXP14.ATEXPA14.ATHEXP ALPHA 1.5.EXP14.EXPA14	expansin A14
Pp3c11_26120	AT3G19820	CBB1.DIM.DIM1.DWF1.EVE1	cell elongation protein / DWARF1 / DIMINUTO (DIM)
Pp3c11_26710	AT5G53480	NaN	ARM repeat superfamily protein
Pp3c12_3560	AT4G09680	ATCTC1.CTC1	conserved telomere maintenance component 1
Pp3c12_4560	AT5G02260	ATEXP9.ATEXPA9.ATHEXP ALPHA 1.10.EXP9.EXPA9	expansin A9
Pp3c12_8200	AT3G54010	DEI1.PAS1	FKBP-type peptidyl-prolyl cis-trans isomerase family protein
Pp3c12_12510	AT2G19080	NaN	metaxin-related
Pp3c12_14250	AT4G14960	TUA6	Tubulin/FtsZ family protein
Pp3c12_14500	AT2G46180	GC4	golgin candidate 4
Pp3c12_23740	AT4G22910	CCS52A1.FZR2	FIZZY-related 2
Pp3c12_26130	AT4G20330	NaN	Transcription initiation factor TFIIE. beta subunit
Pp3c13_5300	AT5G13120	ATCYP20-2.CYP20-2	cyclophilin 20-2
Pp3c13_5760	AT5G67320	HOS15	WD-40 repeat family protein
Pp3c13_7040	AT3G23990	HSP60.HSP60-3B	heat shock protein 60
Pp3c13_8840	AT5G62410	ATCAP-E1.ATSMC4.SMC2.TTN3	structural maintenance of chromosomes 2
Pp3c13_14220	AT1G12840	ATVHA-C.DET3	vacuolar ATP synthase subunit C (VATC) / V-ATPase C subunit (DET3)
Pp3c13_17240	AT3G24350	ATSYP32.SYP32	syntaxin of plants 32
Pp3c13_20230	AT4G38130	ATHD1.ATHDA19.HD1.HDA1.HDA19.RPD3A	histone deacetylase 1
Pp3c13_20360	AT5G02260	ATEXP9.ATEXPA9.ATHEXP ALPHA 1.10.EXP9.EXPA9	expansin A9
Pp3c13_22280	AT4G05410	YAO	Transducin/WD40 repeat-like superfamily protein
Pp3c14_1640	AT5G27540	emb2473.MIRO1	MIRO-related GTP-ase 1
Pp3c14_1940	AT3G55340	PHIP1	phragmoplastin interacting protein 1
Pp3c14_3850	AT5G18700	EMB3013.RUK	Protein kinase family protein with ARM repeat domain
Pp3c14_7230	AT1G15880	atgos11.GOS11	golgi snare 11
Pp3c14_18020	AT1G75950	ASK1.ATSKP1.SKP1.SKP1A.UIP1	S phase kinase-associated protein 1
Pp3c14_18440	AT5G56320	ATEXP14.ATEXPA14.ATHEXP ALPHA 1.5.EXP14.EXPA14	expansin A14
Pp3c14_18590	AT5G56320	ATEXP14.ATEXPA14.ATHEXP ALPHA 1.5.EXP14.EXPA14	expansin A14
Pp3c14_19140	AT2G34050	NaN	NaN
Pp3c14_20080	AT3G51790	ATG1.TG1	transmembrane protein G1P-related 1
Pp3c14_22450	AT5G50375	CPI1	cyclopropyl isomerase
Pp3c14_22830	AT5G56280	CSN6A	COP9 signalosome subunit 6A
Pp3c14_23400	AT2G37340	ATRSZ33.RSZ33	arginine/serine-rich zinc knuckle-containing protein 33
Pp3c15_1000	AT4G05410	YAO	Transducin/WD40 repeat-like superfamily protein
Pp3c15_3730	AT5G65930	KCBP.PKCBP.ZWI	kinesin-like calmodulin-binding protein (ZWICHEL)
Pp3c15_11320	AT1G13980	EMB30.GN.VAN7	sec7 domain-containing protein
Pp3c15_12700	AT2G16485	NaN	nucleic acid binding;zinc ion binding;DNA binding
Pp3c15_13540	AT4G24440	NaN	transcription initiation factor IIA gamma chain / TFIIA-gamma (TFIIA-S)
Pp3c15_14600	AT5G15540	ATSCC2.EMB2773.SCC2	PHD finger family protein
Pp3c15_15310	AT4G15770	NaN	RNA binding
Pp3c15_17790	AT3G57670	NTT.WIP2	C2H2-type zinc finger family protein
Pp3c15_19200	AT5G56740	HAC07.HAC7.HAG02.HAG2	histone acetyltransferase of the GNAT family 2
Pp3c15_21650	AT2G31660	SAD2.URM9	ARM repeat superfamily protein
Pp3c15_22100	AT1G02090	ATCSN7.COP15.CSN7.FUS5	Proteasome component (PCI) domain protein
Pp3c16_3270	AT2G46520	NaN	cellular apoptosis susceptibility protein. putative / importin-alpha re-exporter. putative
Pp3c16_4650	AT2G16950	ATTRN1.TRN1	transportin 1
Pp3c16_5350	AT1G15880	atgos11.GOS11	golgi snare 11
Pp3c16_9360	AT2G36990	ATSIG6.SIG6.SIGF.SOLDAT8	RNApolymerase sigma-subunit F
Pp3c16_11330	AT2G21170	PDTPI.TIM	triosephosphate isomerase
Pp3c16_11390	AT2G27100	SE	C2H2 zinc-finger protein SERRATE (SE)
Pp3c16_12940	AT3G27080	TOM20-3	translocase of outer membrane 20 kDa subunit 3
Pp3c16_15220	AT4G00100	ATRPS13A.PFL2.RPS13.RPS13A	ribosomal protein S13A
Pp3c16_16290	AT5G27540	emb2473.MIRO1	MIRO-related GTP-ase 1
Pp3c16_20770	AT5G45390	CLPP4.NCLPP4	CLP protease P4
Pp3c17_9870	AT4G39460	SAMC1.SAMT1	S-adenosylmethionine carrier 1
Pp3c17_12020	AT3G26618	ERF1-3	eukaryotic release factor 1-3
Pp3c17_12980	AT4G28250	ATEXPB3.ATHEXP BETA 1.6.EXPB3	expansin B3
Pp3c17_15750	AT3G53650	NaN	Histone superfamily protein
Pp3c17_19520	AT5G66750	ATDDM1.CHA1.CHR01.CHR1.DDM1.SOM1.SOM4	chromatin remodeling 1
Pp3c17_19810	AT4G33240	FAB1A	1-phosphatidylinositol-4-phosphate 5-kinases;zinc ion binding
Pp3c17_20760	AT5G66410	PLP3b	phosducin-like protein 3 homolog
Pp3c18_40	AT1G54960	ANP2.MAPKKK2.NP2	NPK1-related protein kinase 2
Pp3c18_5230	AT1G54380	NaN	spliceosome protein-related
Pp3c18_12190	AT3G60830	ARP7.ATARP7	actin-related protein 7
Pp3c18_15660	AT5G50810	TIM8	translocase inner membrane subunit 8
Pp3c18_17930	AT5G56600	PFN3.PRF3	profilin 3
Pp3c18_19660	AT5G02260	ATEXP9.ATEXPA9.ATHEXP ALPHA 1.10.EXP9.EXPA9	expansin A9
Pp3c18_20150	AT2G07050	CAS1	cycloartenol synthase 1
Pp3c18_20160	AT5G23530	AtCXE18.CXE18	carboxyesterase 18
Pp3c18_20180	AT3G16640	TCTP	translationally controlled tumor protein
Pp3c19_2490	AT5G55280	ATFTSZ1-1.CPFTSZ.FTSZ1-1	homolog of bacterial cytokinesis Z-ring protein FTSZ 1-1

Pp3.3 gene*	Best hit At gene*	At_symbol*	At_description*
Pp3c19_3524	AT1G54385	NaN	ARM repeat superfamily protein
Pp3c19_4120	AT5G63840	PSL5.RSW3	Glycosyl hydrolases family 31 protein
Pp3c19_4700	AT2G20490	EDA27.NOP10	nucleolar RNA-binding Nop10p family protein
Pp3c19_4870	AT1G14830	ADL1C.ADL5.DL1C.DRP1C	DYNAMIN-like 1C
Pp3c19_7110	AT3G61650	TUBG1	gamma-tubulin
Pp3c19_9370	AT2G30050	NaN	transducin family protein / WD-40 repeat family protein
Pp3c19_12930	AT2G30910	ARPC1.ARPC1A	actin-related protein C1A
Pp3c19_14090	AT2G16070	PDV2	plastid division2
Pp3c19_16000	AT2G41740	ATVLN2.VLN2	villin 2
Pp3c20_5010	AT1G69390	ARC12.ATMINE1.MINE1	homologue of bacterial MinE 1
Pp3c20_5690	AT3G57290	ATEIF3E-1.ATINT6.EIF3E.INT-6.INT6.TIF3E1	eukaryotic translation initiation factor 3E
Pp3c20_6180	AT5G67270	ATEB1C.ATEB1H1.EB1C	end binding protein 1C
Pp3c20_6200	AT3G46560	emb2474.TIM9	Tim10/DDP family zinc finger protein
Pp3c20_6790	AT4G27060	CN.SPR2.TOR1	ARM repeat superfamily protein
Pp3c20_8320	AT5G48030	GFA2	gametophytic factor 2
Pp3c20_9520	AT5G62440	NaN	Protein of unknown function (DUF3223)
Pp3c20_15610	AT3G12160	ATRABA4D.RABA4D	RAB GTPase homolog A4D
Pp3c20_16750	AT5G06680	ATGCP3.ATSPC98.GCP3.SPC98	spindle pole body component 98
Pp3c20_17290	AT5G54380	THE1	protein kinase family protein
Pp3c21_530	AT5G64930	CPR5.HYS1	CPR5 protein. putative
Pp3c21_1460	AT1G71230	AJH2.CSN5.CSN5B	COP9-signalosome 5B
Pp3c21_3680	AT3G16640	TCTP	translationally controlled tumor protein
Pp3c21_4950	AT4G29340	PRF4	profilin 4
Pp3c21_7106	AT3G17340	NaN	ARM repeat superfamily protein
Pp3c21_8020	AT5G18410	ATSRA1.KLK.PIR.PIR121.PIRP.SRA1	transcription activators
Pp3c21_8640	AT2G40550	ETG1	E2F target gene 1
Pp3c21_10940	AT1G56350	NaN	Peptide chain release factor 2
Pp3c21_11720	AT3G13870	RHD3	Root hair defective 3 GTP-binding protein (RHD3)
Pp3c21_15890	AT5G09420	ATTOC64-V.MTOM64.TOC64-V	translocon at the outer membrane of chloroplasts 64-V
Pp3c22_630	AT4G28250	ATEXPB3.ATHEXP BETA 1.6.EXPB3	expansin B3
Pp3c22_1100	AT1G14830	ADL1C.ADL5.DL1C.DRP1C	DYNAMIN-like 1C
Pp3c22_1770	AT1G29990	PFD6	prefoldin 6
Pp3c22_2850	AT3G61650	TUBG1	gamma-tubulin
Pp3c22_4940	AT5G55280	ATFTSZ1-1.CPFTSZ.FTSZ1-1	homolog of bacterial cytokinesis Z-ring protein FTSZ 1-1
Pp3c22_5670	AT5G64930	CPR5.HYS1	CPR5 protein. putative
Pp3c22_5980	AT5G58230	ATMSI1.MEE70.MSI1	Transducin/WD40 repeat-like superfamily protein
Pp3c22_9040	AT5G50810	TIM8	translocase inner membrane subunit 8
Pp3c22_11100	AT1G48050	ATKU80.KU80	Ku80 family protein
Pp3c22_19420	AT3G20000	TOM40	translocase of the outer mitochondrial membrane 40
Pp3c23_2850	AT5G13120	ATCYP20-2.CYP20-2	cyclophilin 20-2
Pp3c23_5320	AT5G26240	ATCLC-D.CLC-D	chloride channel D
Pp3c23_7840	AT3G12160	ATRABA4D.RABA4D	RAB GTPase homolog A4D
Pp3c23_8330	AT1G78900	VHA-A	vacuolar ATP synthase subunit A
Pp3c23_14790	AT3G24590	PLSP1	plastidic type i signal peptidase 1
Pp3c23_14990	AT2G20990	ATSYTA.NTMC2T1.1.NTMC2TYPE1.1.SYT1.SYTA	synaptotagmin A
Pp3c23_15370	AT5G48030	GFA2	gametophytic factor 2
Pp3c23_19930	AT5G67270	ATEB1C.ATEB1H1.EB1C	end binding protein 1C
Pp3c23_22070	AT1G66740	ASF1A.AtSP7.SGA2.SP7	ASF1 like histone chaperone
Pp3c23_22130	AT1G66740	ASF1A.AtSP7.SGA2.SP7	ASF1 like histone chaperone
Pp3c24_1490	AT5G43970	ATTOM22-V.TOM22-V.TOM9-2	translocase of outer membrane 22-V
Pp3c24_4940	AT5G15920	SMC5	structural maintenance of chromosomes 5
Pp3c24_6930	AT5G66680	DGL1	dolichyl-diphosphooligosaccharide-protein glycosyltransferase 48kDa subunit family protein
Pp3c24_9660	AT5G13120	ATCYP20-2.CYP20-2	cyclophilin 20-2
Pp3c24_11460	AT2G20190	ATCLASP.CLASP	CLIP-associated protein
Pp3c24_12050	AT1G48635	PEX3.PEX3-2	peroxin 3
Pp3c24_12180	AT2G28000	CH-CPN60A.CPN60A.SLP	chaperonin-60alpha
Pp3c24_15930	AT3G46560	emb2474.TIM9	Tim10/DDP family zinc finger protein
Pp3c24_18820	AT1G63680	ATMURE.MURE.PDE316	acid-amino acid ligases;ligases;ATP binding;ATP binding;ligases
Pp3c24_19870	AT4G33650	ADL2.DRP3A	dynamin-related protein 3A
Pp3c24_20920	AT5G42400	ATXR7.SDG25	SET domain protein 25
Pp3c25_660	AT1G21700	ATSWI3C.CHB4.SWI3C	SWITCH/sucrose nonfermenting 3C
Pp3c25_4540	AT2G19480	NAP1;2.NFA02.NFA2	nucleosome assembly protein 1;2
Pp3c26_2180	AT3G23990	HSP60.HSP60-3B	heat shock protein 60
Pp3c26_14180	AT2G20370	KAM1.MUR3	Exostosin family protein
Pp3c27_1990	AT1G04950	ATTAF6.TAF6.TAFII59	TATA BOX ASSOCIATED FACTOR II 59
Pp3c27_3260	AT5G19530	ACL5	S-adenosyl-L-methionine-dependent methyltransferases superfamily protein
Pp3c27_8050	AT4G33880	RSL2	ROOT HAIR DEFECTIVE 6-LIKE 2

*Twenty-eight of 254 genes are colored in red as the common DEGs both 1cell-DGE and 5'DGE.

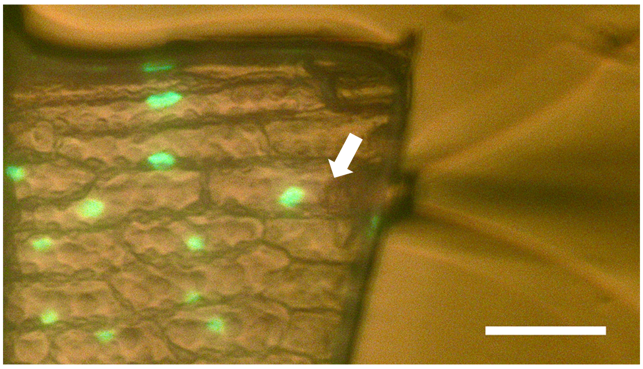

Supplementary Figure S1

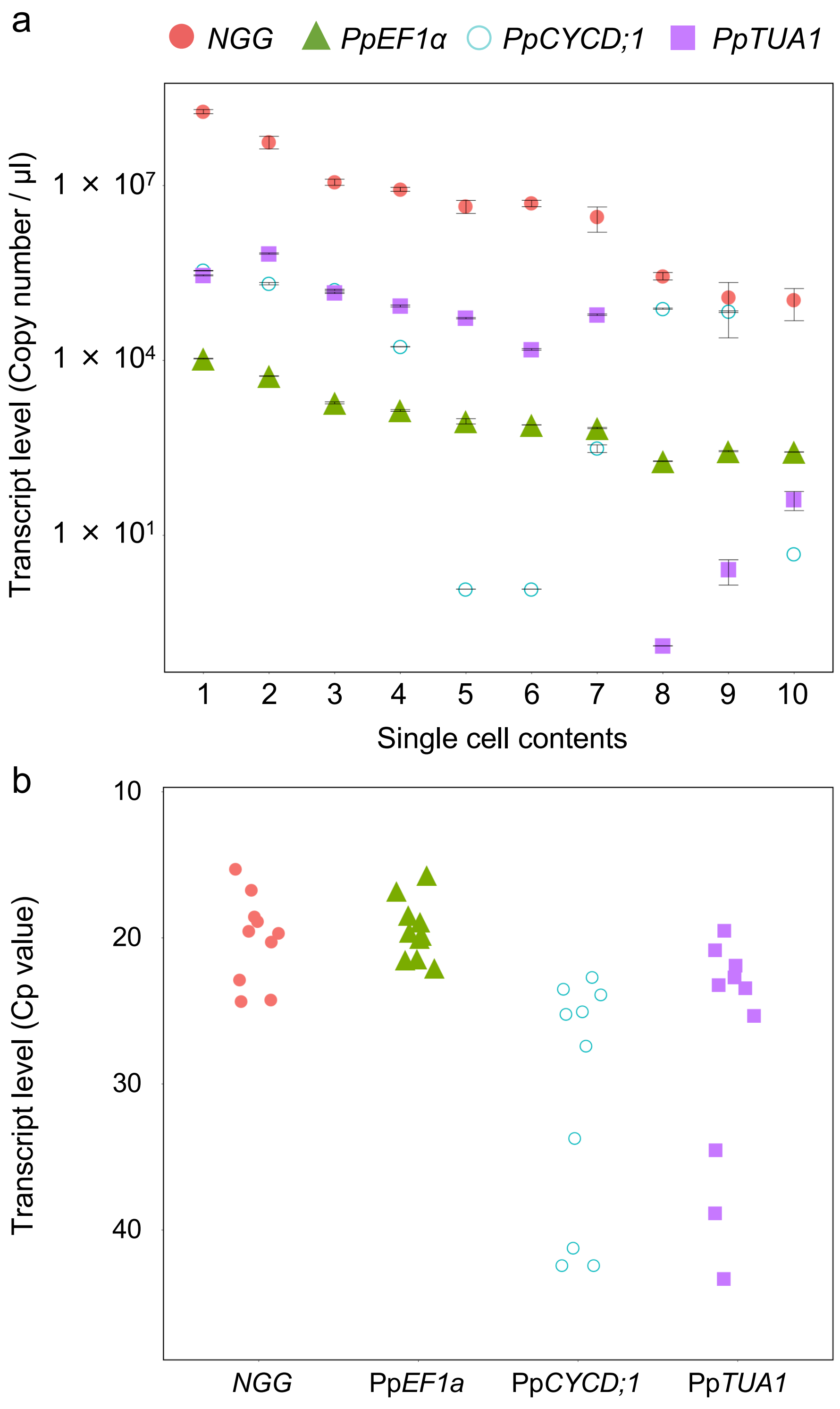

Supplementary Figure S2

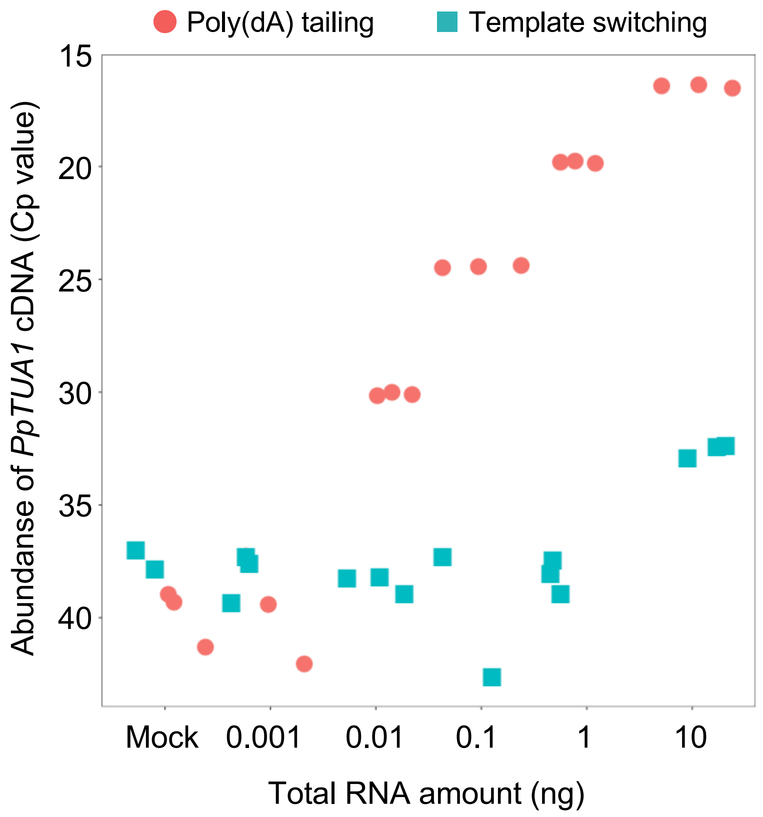

Supplementary Figure S3

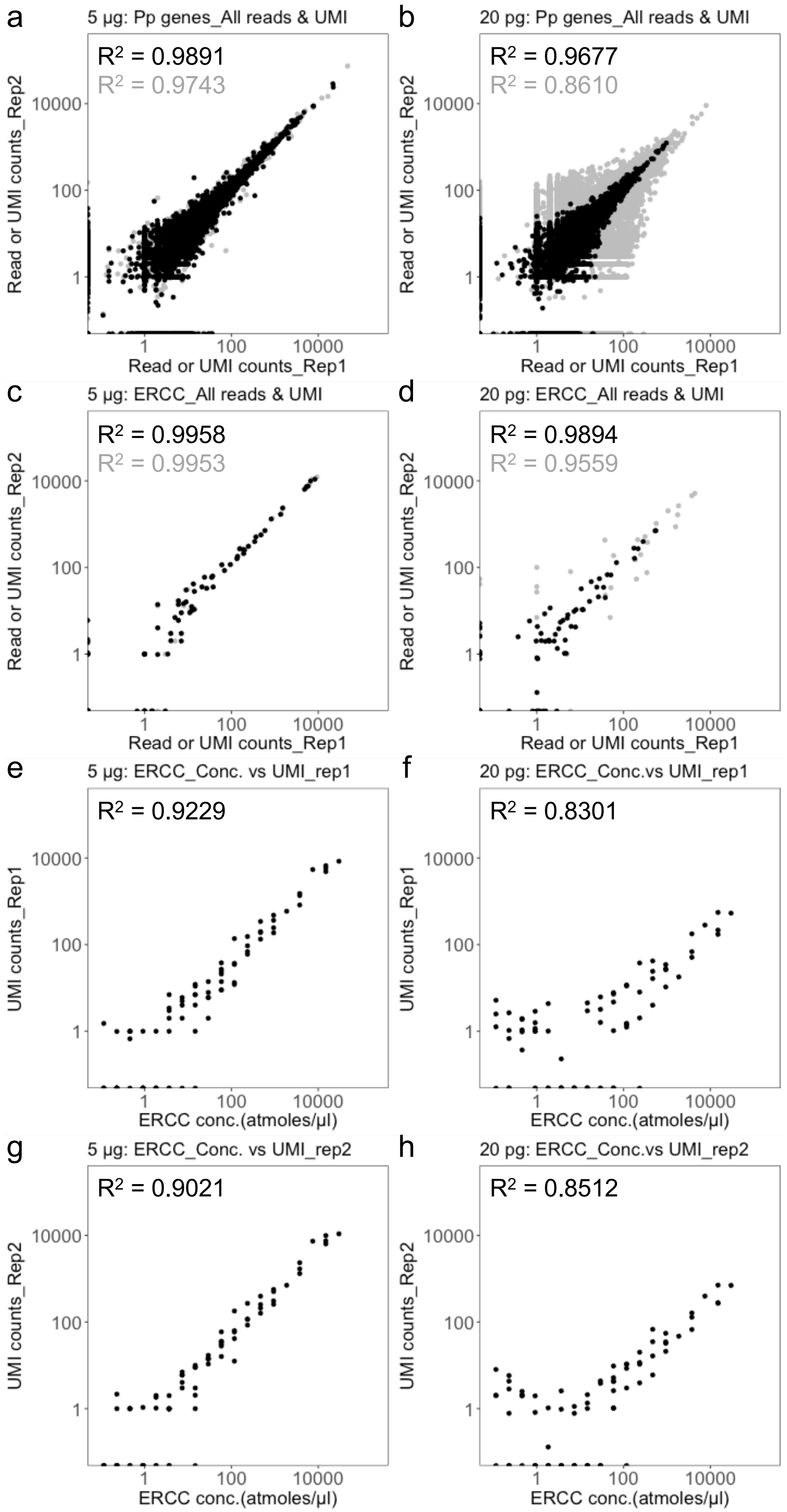

Supplementary Figure S4

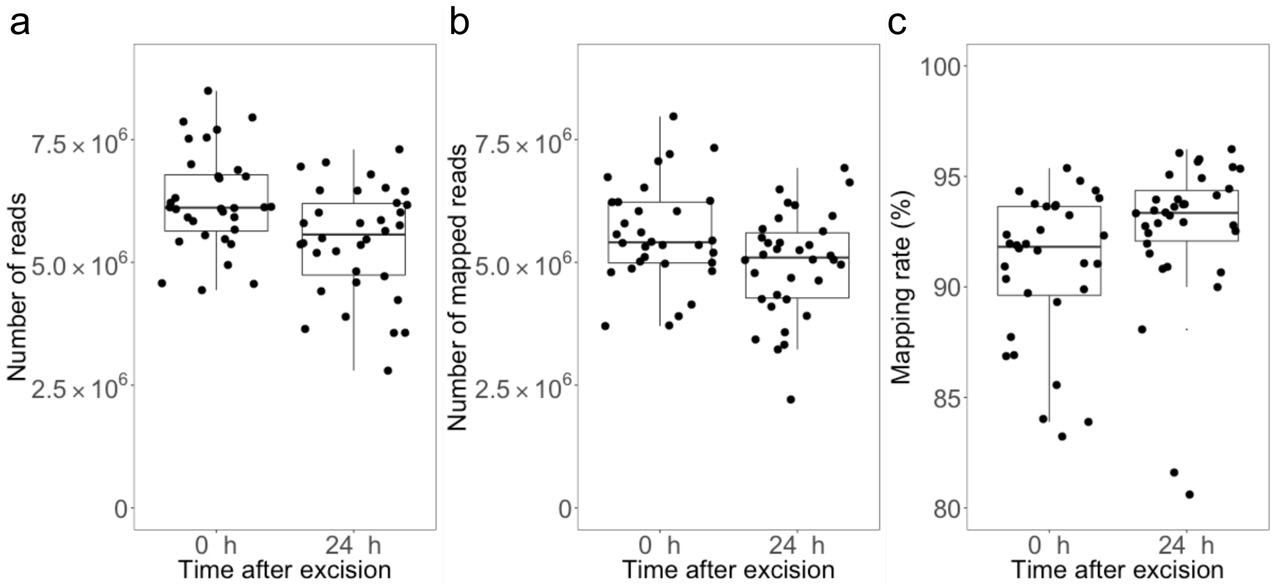

Supplementary Figure S5

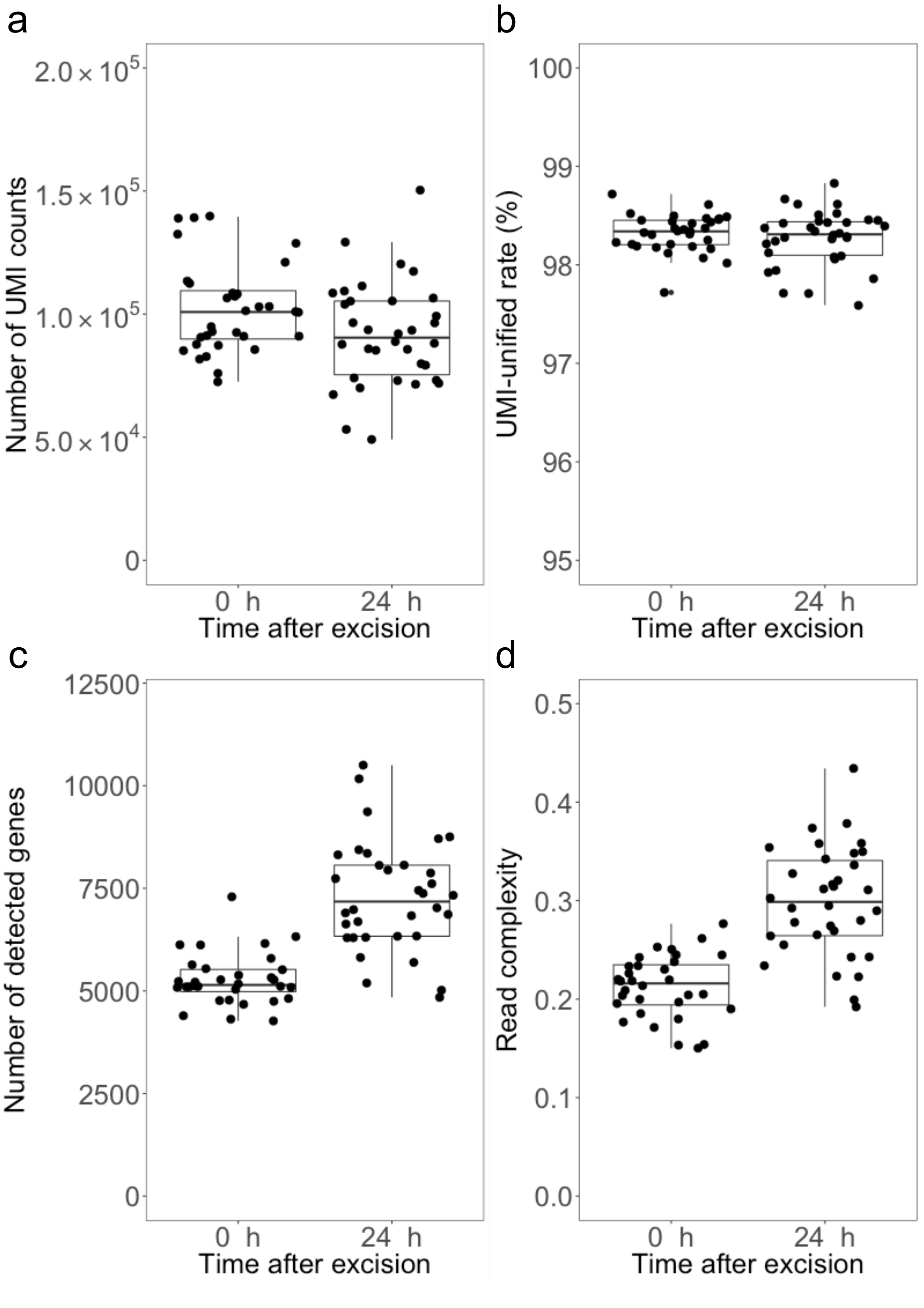

Supplementary Figure S6

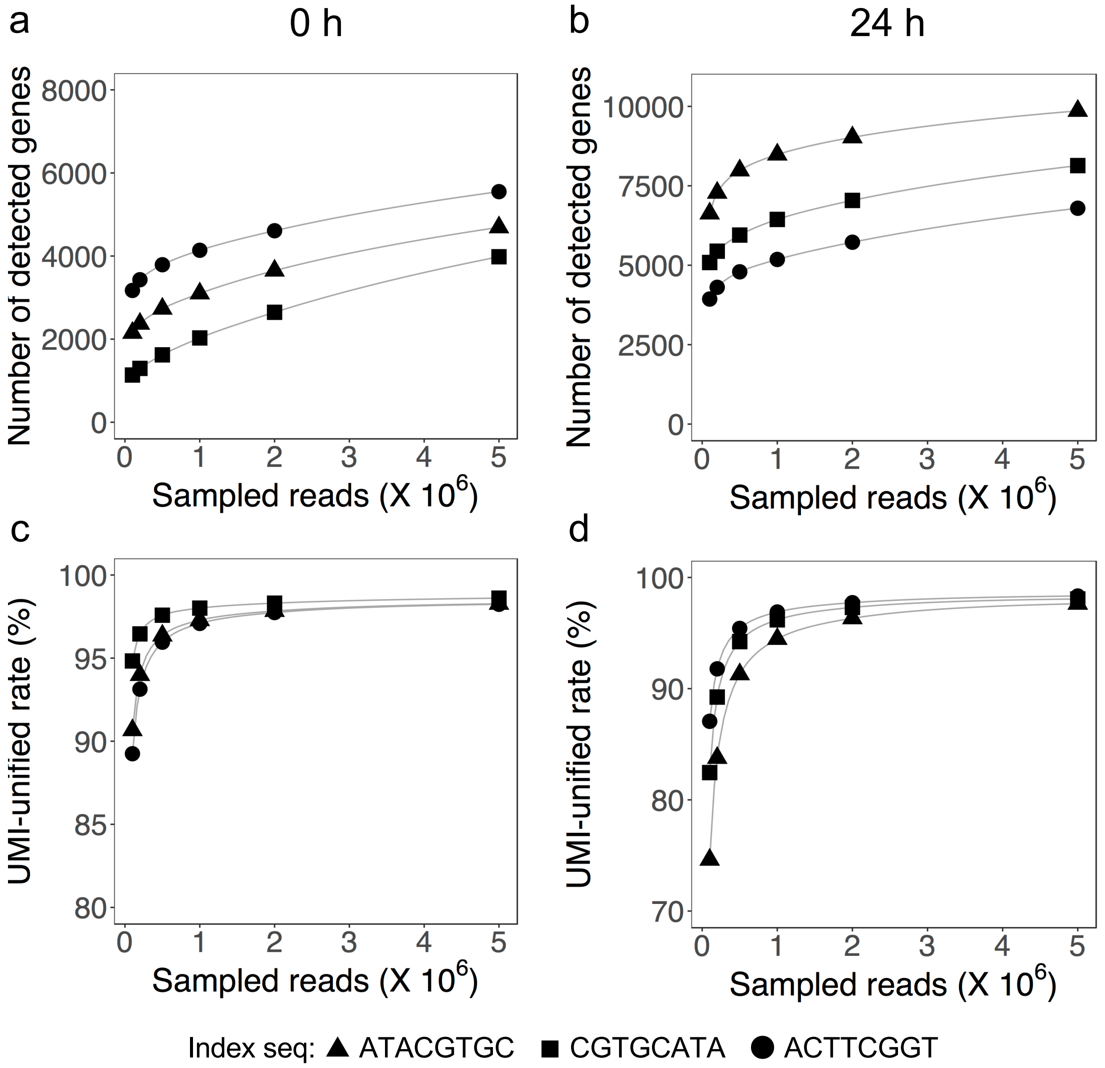

Supplementary Figure S7

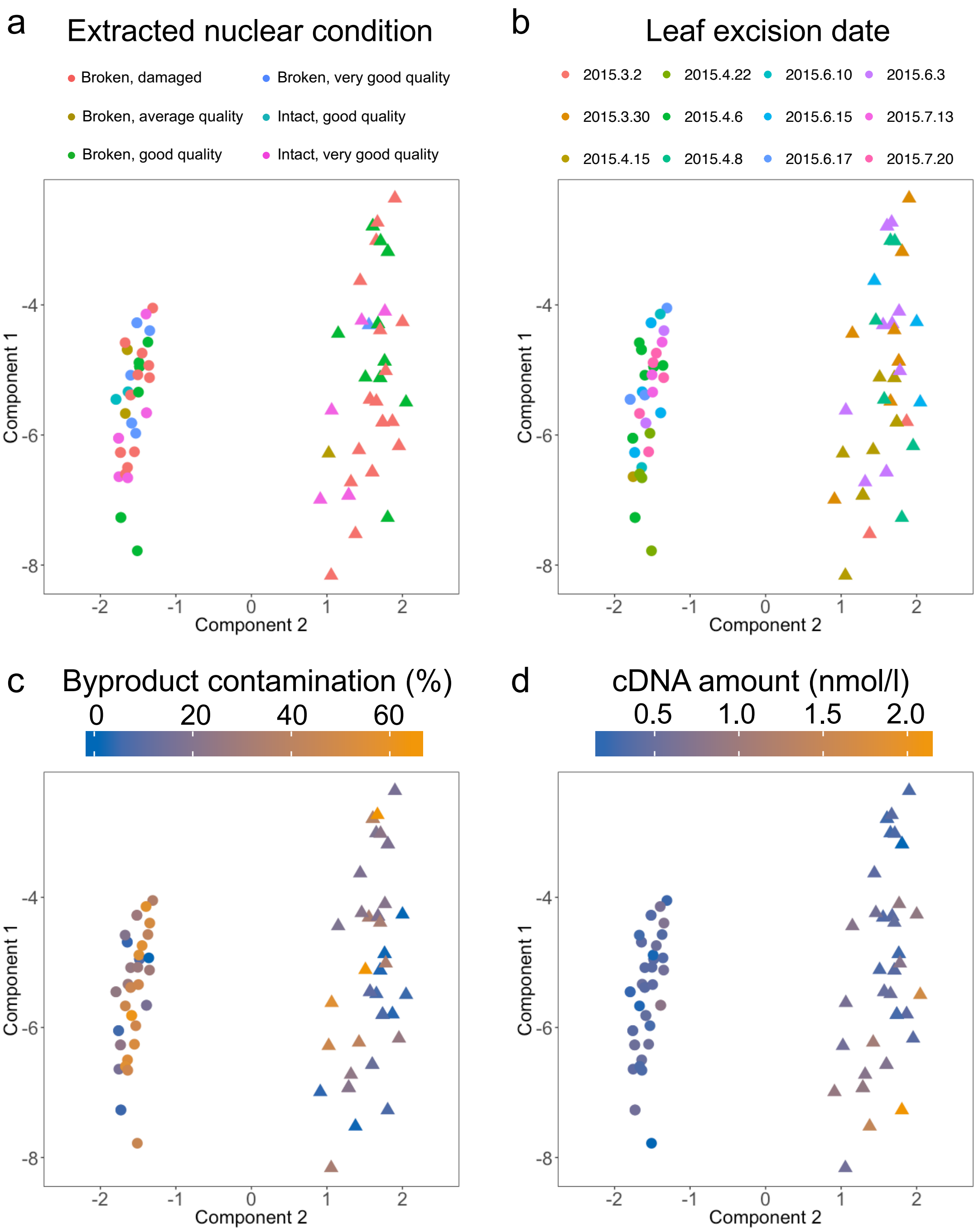

Supplementary Figure S8

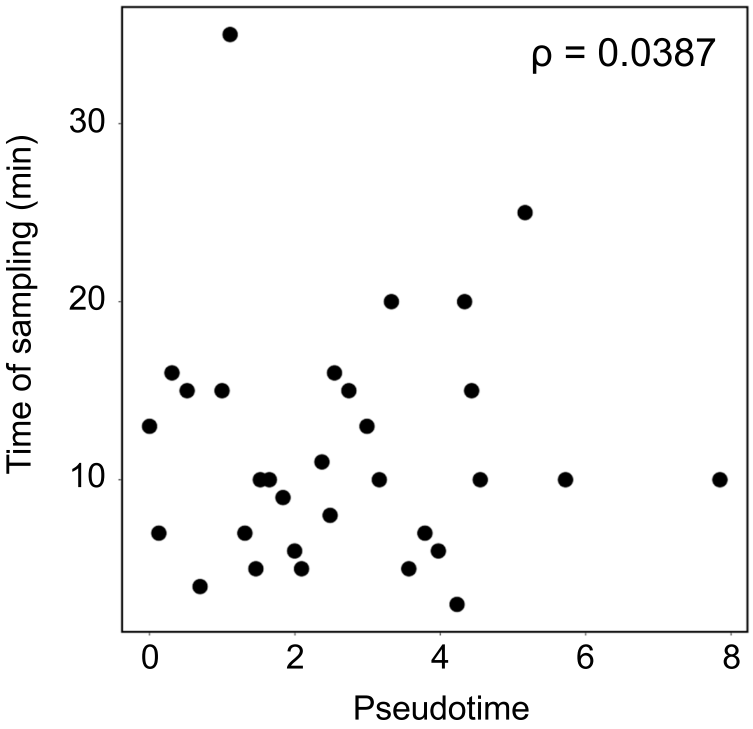

Supplementary Figure S9
